# Next-Generation Carbazole-Linked 1,2,4-Triazole-Thione Derivatives: Strategic Design, Synthesis, Molecular Docking, and Evaluation of Antidiabetic Potential

**DOI:** 10.1021/acsomega.4c07896

**Published:** 2024-12-25

**Authors:** İrfan Çapan, Mohammed Hawash, Mohammed T. Qaoud, Nidal Jaradat

**Affiliations:** † Department of Pharmaceutical Basic Sciences, Faculty of Pharmacy, Gazi University, 06330 Ankara, Turkey; ‡ Sente Kimya Research and Development Inc., 06200 Ankara, Turkey; § Department of Pharmacy, Faculty of Medicine and Health Sciences, An-Najah National University, 00433 Nablus, Palestine; ∥ Department of Pharmacy, Faculty of Pharmacy, Cyprus International University, Northern Cyprus, Mersin 10, 99258 Nicosia, Turkey

## Abstract

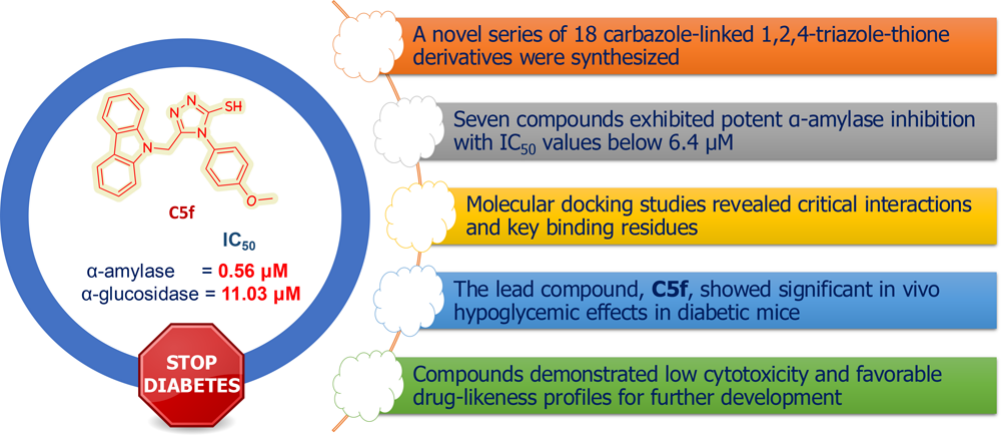

Currently, available therapies for diabetes cannot achieve normal sugar values in a high percentage of treated patients. This work synthesized a series of carbazole-triazole-thione derivatives, and their potential antidiabetic activity was investigated against the key diabetic enzymes α-amylase and glycosidase. Normal human hepatic stellate cells (LX-2) were employed to assess their cytotoxicity and safety, followed by in vivo testing to investigate the hypoglycemic effect of the most promising agent. As a result, a set of 18 carbazole-1,2,4-triazole-thione derivatives were synthesized. Seven structures demonstrated potential inhibitory activity against α-amylase enzyme, with IC_50_ lower than 6.4 μM. Among them, compounds **C5f**, **C5o**, and **C5r** exhibited the highest potency, with IC_50_ values of 0.56, 0.53, and 0.97 μM, respectively, compared to the well-known α-amylase inhibitor acarbose, which has an IC_50_ value of 5.31 μM. Exploring the inhibition potency of these series against α-glucosidase enzyme revealed that **C5f** and **C5r** compounds act as moderate inhibitors, with IC_50_ values of 11.03 and 13.76 μM, respectively. Moreover, at 100 μM concentration, most of the evaluated compounds showed negligible cytotoxic effect against LX-2 cell lines, particularly compounds **C5o** and **C5s**, that demonstrated lower cytotoxic activity by 3-fold compared to the positive control 5-Flururicle (cell viability 13.45%). Thus, the **C5f** compound was selected for in vivo evaluation, and after administering five doses of this compound (10 mg/kg) to group III of mice, a significant reduction in glucose concentration was observed, bringing it down from 290.54 to 216.15 mg/dL, in comparison with the control group which did not show a reduction in blood glucose level. These observed in vitro and in vivo results were upheld by performing a set of chemoinformatic studies that elucidated the binding interactions of the most active derivatives within the enzyme’s active site and highlighted the critical roles of both the 1,2,4-triazole-3-thione and carbazole scaffolds in these interactions. Finally, the drug-likeness profiles of our carbazole-triazole-thione derivatives suggest their potential as candidates for further in vivo studies and clinical trials.

## 1 Introduction

In 2014, the World Health Organization (WHO) identified a staggering 422 million individuals globally afflicted with diabetes. The International Diabetes Federation further elucidated that in 2021, the worldwide prevalence of diabetes among adults aged 20–79 was approximately 10.5%, encompassing roughly 536.6 million people. Projections indicate this prevalence will escalate to 12.2%, or 783.2 million individuals, by 2045.^1^ Diabetes represents a critical and prevalent metabolic disorder with significant implications for global health.^2^ Notably, insulin resistance, which affects approximately 95% of diabetes patients, precipitates dysregulation of blood glucose levels.^3^ This pathophysiological condition raises the risk of a range of diseases, including cardiovascular complications, cerebrovascular accidents, hypertension, and renal failure.^4^

Starch constitutes a primary source of carbohydrates, which are vital nutrients in the human diet. The enzyme α-amylase, composed of 1,4-linked polysaccharides such as glucose, plays a crucial role in hydrolyzing starch. This process, in conjunction with another intestinal glycosidase, facilitates the absorption of starch into the body.^5^ Meanwhile, α-glucosidase catalyzes the breakdown of polysaccharides and oligosaccharides into monosaccharides like fructose and glucose, thereby participating in the terminal phase of carbohydrate digestion.^6^ Therefore, inhibiting these enzymes is considered a rational and promising strategy to mitigate postprandial blood glucose spikes. This approach can aid in managing type 2 diabetes mellitus (T2DM) by regulating blood glucose levels, akin to the mechanism of action of the FDA-approved drug Acarbose.^7^ Several α-amylase inhibitors are recognized, including phenolic compounds, tannins, anthocyanins, and saponins.^8^ Although numerous antidiabetic medications are currently available, their usage is often accompanied by adverse effects and insufficient efficacy in preventing chronic complications, such as hepatotoxicity, diarrhea, flatulence, abdominal pain, and persistent hyperglycemia.^9^

**Figure 1:**
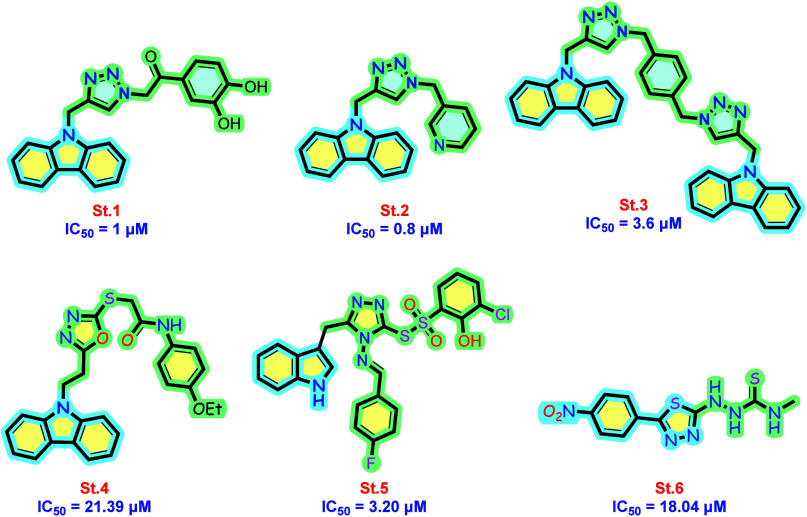
Some carbazole and azole derivatives with potential α-glucosidase inhibitory activity.

In recent decades, carbazole and triazole derivatives have garnered significant attention within medicinal chemistry due to their broad spectrum of biological and pharmacological activities. These compounds exhibit diverse effects, including antibacterial, antitumor, antioxidant, and anti-inflammatory properties, as well as potential antidiabetic activity, thereby highlighting their substantial potential for application across various therapeutic domains.^10^ Moreover, investigations on streptozotocin-induced diabetic rats have demonstrated the ability of carbazole derivatives to modulate the AKT-dependent signaling pathway in L6-GLUT-4 myc myotubes. This modulation results in the insulin-responsive glucose transporter (GLUT-4 receptor) translocation, indicating promising antidiabetic activity.^11^ These compounds effectively regulate carbohydrate metabolism by managing insulin resistance and diabetes, thereby underscoring their multifaceted impact on diabetic pathology.^12^ In medicinal chemistry, synthesizing novel drug candidates through molecular hybridization, where two or more distinct pharmacophores are combined, offers significant promise. This approach enables the creation of hybrid molecules that can potentially exhibit enhanced binding interactions and more effective receptor pocket accommodation, provided that the biological activities of the compounds are thoroughly evaluated.^13^ Following this strategy, as illustrated in Figure 1, Iqbal et al. designed a series of hybrid derivatives incorporating 1,2,3-triazole and carbazole scaffolds (**st.1–st.3**). This approach led to the discovery of novel carbazole-bearing triazole candidates that exhibited promising α-glucosidase inhibitory activity within the single-digit micromolar range, along with negligible cytotoxicity.^14^ In a recent study, a series of novel antidiabetic agents were synthesized by incorporating a heteroaromatic oxadiazole ring with a carbazole scaffold, with compound **st.4** emerging as the most promising candidate. This compound exhibited moderate α-glucosidase inhibitory activity, achieving an IC_50_ value of 21.39 μM. However, its efficacy against α-amylase was relatively weaker, with an IC_50_ of 60 μM. Notably, **st.4** demonstrated excellent cytotoxicity and pharmacokinetic profiles.^15^ As part of a novel coupling strategy, Shoaib Khan et al. synthesized a series of indole-based triazole-derived sulfonothioate derivatives that demonstrated promising inhibitory potency against both α-amylase and α-glucosidase enzymes. For instance, compound **St.5** exhibited an IC_50_ of approximately 3 μM against both enzymes, indicating its potential as an antidiabetic agent.^16^**St.6** represents a novel hybrid series featuring a 1,3,4-thiadiazole-bearing thiosemicarbazide moiety, demonstrating potent inhibitory activity against both α-glucosidase and α-amylase enzymes, with IC_50_ values of 18.04 μM and 21.02 μM, respectively. These promising results highlight the potential of hybridization strategies in discovering new, effective antidiabetic agents.^17^

Building on the aforementioned insights, we tackle the challenge of identifying innovative antidiabetic drug scaffolds that combine high efficacy with minimal toxicity. As the 1,2,4-triazole-3-thiones have recently emerged as highly effective pharmacophores with a broad spectrum of biological activities and are extensively characterized in pharmaceutical research, making them a preferred scaffold for developing new bioactive molecules,^18^ this study outlines the synthesis of a novel hybrid compound integrating a 1,2,4-triazole-3-thione moiety with a carbazole ring. The aim is to develop a more potent antidiabetic agent with reduced adverse effects. Furthermore, the study assessed the antidiabetic efficacy of the newly synthesized derivatives, including their inhibitory effects on α-amylase and α-glucosidase enzymes in vitro. Additionally, the cytotoxic effects of these compounds on human hepatic stellate cells (LX-2) were investigated, and an in vivo study was conducted on selected compounds to unveil the impact on blood glucose levels in STZ-induced diabetic mice. Subsequently, an array of chemoinformatic analyses, including detailed molecular docking simulations, were conducted to elucidate the interactions and identify the critical functional groups of the most promising derivatives within the active sites of α-amylase and α-glucosidase. Finally, the drug-likeness of the newly synthesized compounds was rigorously evaluated to determine their potential for advancement into clinical trials.

## 2 Results and Discussion

### 2.1 Chemistry

**Scheme 1:**
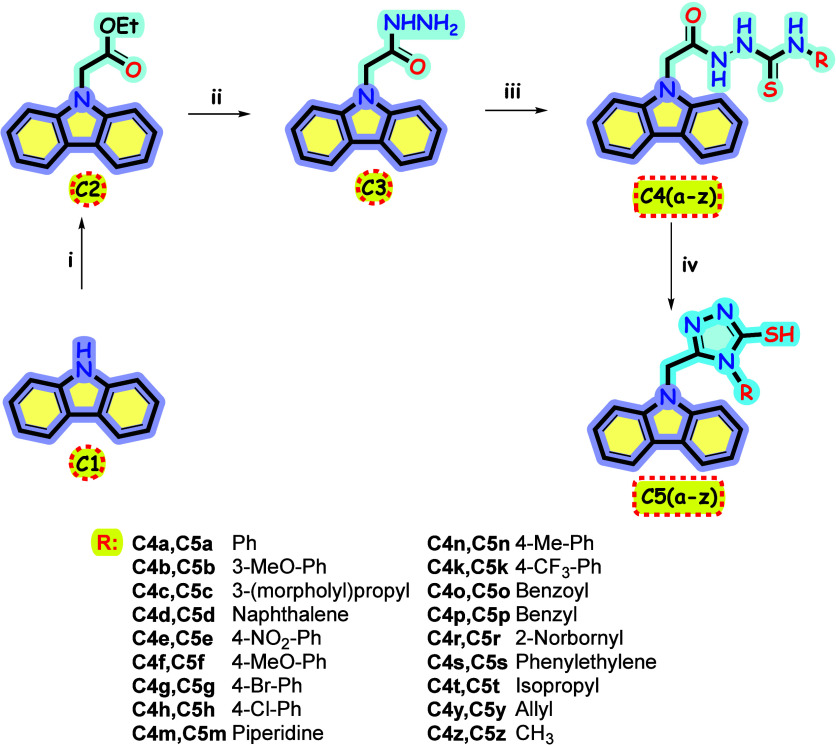
Reagents and Conditions: (i) Ethyl Bromoacetate, NaH, DMF, rt.; (ii) NH_2_NH_2_·H_2_O, Ethanol, Refluxed, 12 h.; (iii) Isothiocyanate Derivatives, Ethanol, Refluxed, 5 h; (iv) 2 N NaOH_(aq)_, 100 °C, 4 h

The reaction of carbazole, which is the target skeleton of the study, with NaH, a very strong base, in DMF solvent was initiated with the addition of ethyl bromacetate to produce ester compound **2**. From there, using hydrazine hydrate, we obtained hydrazide compound **3**. As a result of the addition reactions of different isothiocyanates with compound **3** in ethyl alcohol, carbothioamides (**C4a–z**) with carbazole skeleton were obtained. The prepared **C4a–z** compounds and all previous intermediates (**C1**, **C2**, and **C3**) were synthesized in our previous studies.^19^ In the last step, the targeted 1,2,4-triazole-thione ring system was synthesized in high yields by ring shutting reaction of compounds **4a**–**z** heated with 2N aqueous solution of NaOH. The general reaction scheme is given in Scheme 1.

### 2.2 Antidiabetic Activity

Several therapeutic methods can monitor hyperglycemia, one being the inhibition of α-amylase and α-glucosidase metabolic enzymes.^20^ The α-amylase is a responsible enzyme for the reactions of hydrolysis of starch into monosaccharides.^21^ The α-glucosidase enzyme can be found on the border of the small intestine and acts upon hydrolysis of α-(1,4) bonds between monosaccharide units.^22^

The antidiabetic potential of the synthesized compounds was estimated by assessing their α-amylase and α-glycosidase inhibitory effects. Acarbose, which is a commercial antidiabetic drug, was used as a positive control. The IC_50_ values were calculated and are shown in Table 1. Among the 19 newly synthesized compounds, **C5f** and **C5r** exhibited notable inhibitory activity against the key diabetic enzyme α-amylase, with potencies in the nanomolar range, though they showed lower efficacy against α-glucosidase. Conversely, compounds **C5g**, **C5h**, **C5m**, and **C5o** demonstrated exclusive inhibition potency against α-amylase, with IC_50_ values ranging from 0.53 to 6.4 μM. The remaining carbazole-linked 1,2,4-triazole-thione derivatives displayed moderate to weak activity against both enzymes, with IC_50_ values exceeding 23 μM.

**Table 1:** Activity of **C5a–z** Series against α-Amylase and α-Glucosidase Enzymes^a^

code	IC_50_ μM				
	α-amylase	α-glucosidase	code	α-amylase	α-glucosidase
C5a	55.25 ± 2.54	61.46 ± 3.01	C5n	>100	>100
C5b	66.23 ± 2.03	>100	**C5o**	0.53 ± 0.24	>100
C5c	23.29 ± 1.47	>100	C5p	>100	>100
C5d	24.54 ± 0.88	>100	**C5r**	**0.97 ± 0.47**	**13.76 ± 1.55**
C5e	32.94 ± 2.04	>100	C5s	>100	>100
**C5f**	**0.56 ± 0.24**	11.03 ± 1.03	C5t	>100	>100
C5k	>100	80.61 ± 3.17	C5y	>100	>100
C5g	4.83 ± 0.75	>100	C5z	NA	NA
C5h	6.40 ± 1.74	>100	acarbose	5.31 ± 2.40	130.45 ± 2.41
C5m	2.39 ± 0.45	>100			

a NA: not applicable due to the solubility.

### 2.3 In Vitro Cytotoxicity Evaluation in Normal Hepatic Cells (LX-2)

**Figure 2:**
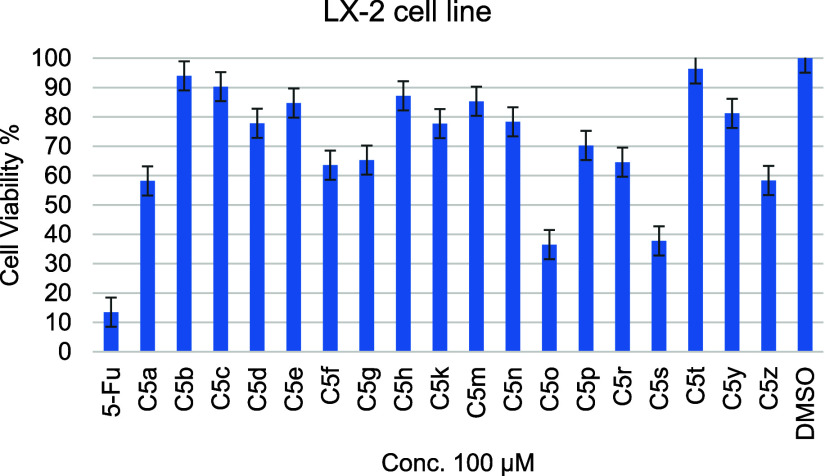
Cell viability percentage at a concentration of 100 μM of Carbazole derivatives, positive control (5-Fu), and negative control (DMSO) on LX-2 normal cell line.

MTS (3-(4,5-dimethylthiazol-2-yl)-5-(3-carboxymethoxyphenyl)-2-(4-sulfophenyl)-2H-tetrazolium) assay was used to determine the cytotoxicity effect of Carbazole-triazole-thione derivatives on LX-2 (normal hepatic cell). The LX-2 cell lines are usually utilized to evaluate the normal cell lines’ cytotoxicity compared with the target cancer cells or other biological targets like enzymes and receptors.^23^ The percentage of cell viability

on the LX-2 cell line of Carbazole derivatives, the positive control 5-Fluorouracil (5-Fu), and the negative control (DMSO) at a concentration of 100 μM was shown in Figure 2. 5-Fu was used as a positive control in this context due to its well-documented cytotoxic effects on various cell lines, including hepatic cells like LX-2.^24^ As a well-established chemotherapeutic agent, 5-Fu is a reliable benchmark for assessing the tested compounds’ cytotoxicity. The percentage was 36.46–96.37% for all evaluated compounds, compared with a positive control anticancer drug percentage value of 13.45%. A higher percentage of cell viability indicates a greater proportion of cells that remain alive after exposure to the tested agents, while lower viability percentages suggest increased cytotoxicity. Based on this, compounds **C5o** and **C5s** emerged as the most cytotoxic, with cell viability values of 36.46 and 37.75%, respectively, compared to the positive control 5-FU and the negative control DMSO. Most newly synthesized carbazole-linked 1,2,4-triazole-thione derivatives exhibited negligible cytotoxic effects on LX-2 cells. Specifically, compounds **C5f** and **C5r**, which demonstrated the most potent activity against both key enzymes, showed cytotoxicity percentages of 63.56 and 64.55%, respectively, at a concentration of 100 μM. Compounds selectively potent against α-amylase (**C5g**, **C5h**, and **C5m**) showed cell viability percentages ranging between 65.26 and 85.31% at the same concentration. This indicates that the newly designed series, at active concentrations of α-amylase, does not exhibit cytotoxic effects. Furthermore, their high selectivity ratio suggests that these compounds are safe for use.

#### 2.3.1 In Vivo Hypoglycemic Effect of Compound C5f on Mice with STZ-Induced Diabetes

**Figure 3:**
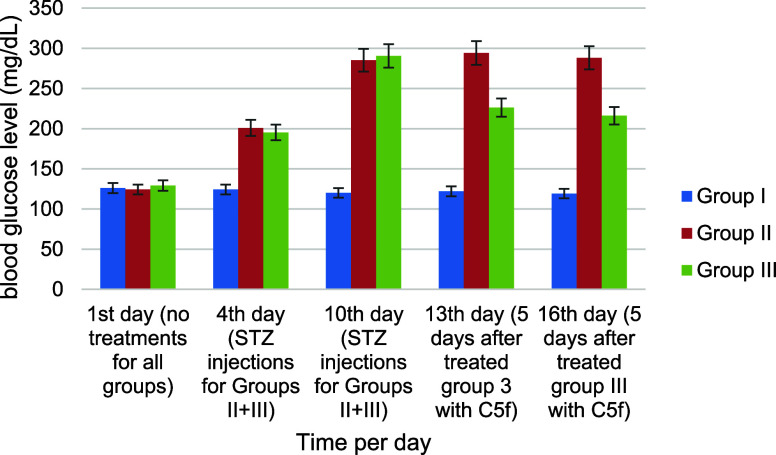
Fasting serum glucose concentration on the first day without any treatment, the fourth and 10th day with STZ for groups II & III, and the 13th and 16th day after treatment for group III with the compound **C5f**. Data are shown as mean ± SD (*n* = 7); *p*-value < 0.05 (group I: negative control mice without any treatment; group II: positive control, mice with STZ treatment only; group III: mice treated with STZ + C5f).

The Carbazole-triazole derivatives were tested in a laboratory setting to determine their ability to inhibit the enzyme α-amylase and α-glucosidase. Among the evaluated compounds, the **C5f** compound showed in vitro promising results with IC_50_ values of 0.56 and 11.03 μM against both α-amylase and α-glucosidase enzymes, respectively. Therefore, **C5f** was selected for further investigation of its ability to lower blood sugar levels in an in vivo study. This investigation sought to reveal the effect of **C5f** on blood glucose levels in mice with diabetes caused by Streptozotocin (STZ). This compound has a significant hypoglycemic effect in STZ-diabetic mice, similar to the effect reported in the control group (mice that were not treated), as shown in Figure 3. The mice were divided into three groups: group I: negative control mice without any treatment; Group II: positive control mice with STZ treatment only; and Group III: mice treated with STZ and then with our compound **C5f**.

The findings indicate that the initial fasting blood glucose levels exhibited no significant differences across all groups. However, after intraperitoneal administration of STZ (40 mg/kg) to groups II and III, the blood glucose level was increased compared to group I, as illustrated in Figure 3. On the fourth day after STZ injections for groups II and III, the average blood glucose levels reached 198.14 ± 4.07 mg/dL (*p*-value < 0.05) compared with untreated group I 124.25 ± 3.08 mg/dL. Similar elevation was recorded on the 10th day for groups II and III, and blood glucose levels average reached 287.88 ± 3.77 mg/dL (*p*-value < 0.05) compared with the untreated group I 120.14 ± 3.02 mg/dL. Upon administering five doses of **C5f** (10 mg/kg) to group III, a significant reduction in blood glucose levels was observed, bringing it down from 290.54 ± 2.15 to 216.15 ± 3.45 mg/dL. This decrease was particularly pronounced in comparison with untreated diabetes group II, which displayed a glucose concentration of 288.13 ± 3.75 mg/dL.

### 2.4 Chemoinformatics Studies

#### 2.4.1 Molecular Docking

Molecular docking simulations are a powerful computational technique widely used in drug discovery. Beyond simply predicting the interactions between small molecule therapeutic compounds and their anticipated protein targets, docking serves as a tool for understanding the modes of action at the molecular level. This method explores the binding affinity, conformations, and orientations of the molecules within the binding pocket and provides critical insights into the molecular mechanisms of interaction. Elucidating these interactions helps identify key binding residues and potential structural optimizations, thus facilitating the rational design of more potent and selective therapeutic agents.^25^

The crystallographic structures with PDB ID codes 4W93 (for α-amylase) and 2ZE0 (for α-glucosidase) were selected for the molecular docking simulations. These protein structures were chosen due to their high-resolution data, with 4W93 having a resolution of 1.35 Å and 2ZE0 at 2 Å, ensuring precision and reliability in the docking analysis. Additionally, to further validate the choice of these structures, acarbose, a well-established inhibitor, was docked as a positive control for both proteins. The docking results for acarbose exhibited ideal interaction profiles and docked poses that aligned closely with previously published data,^26,27^ confirming the suitability of these protein models. Moreover, the positive correlation between the docking scores obtained for the newly designed agents and their experimentally reported inhibition potencies reinforces the validity of the docking results.

**Figure 4:**
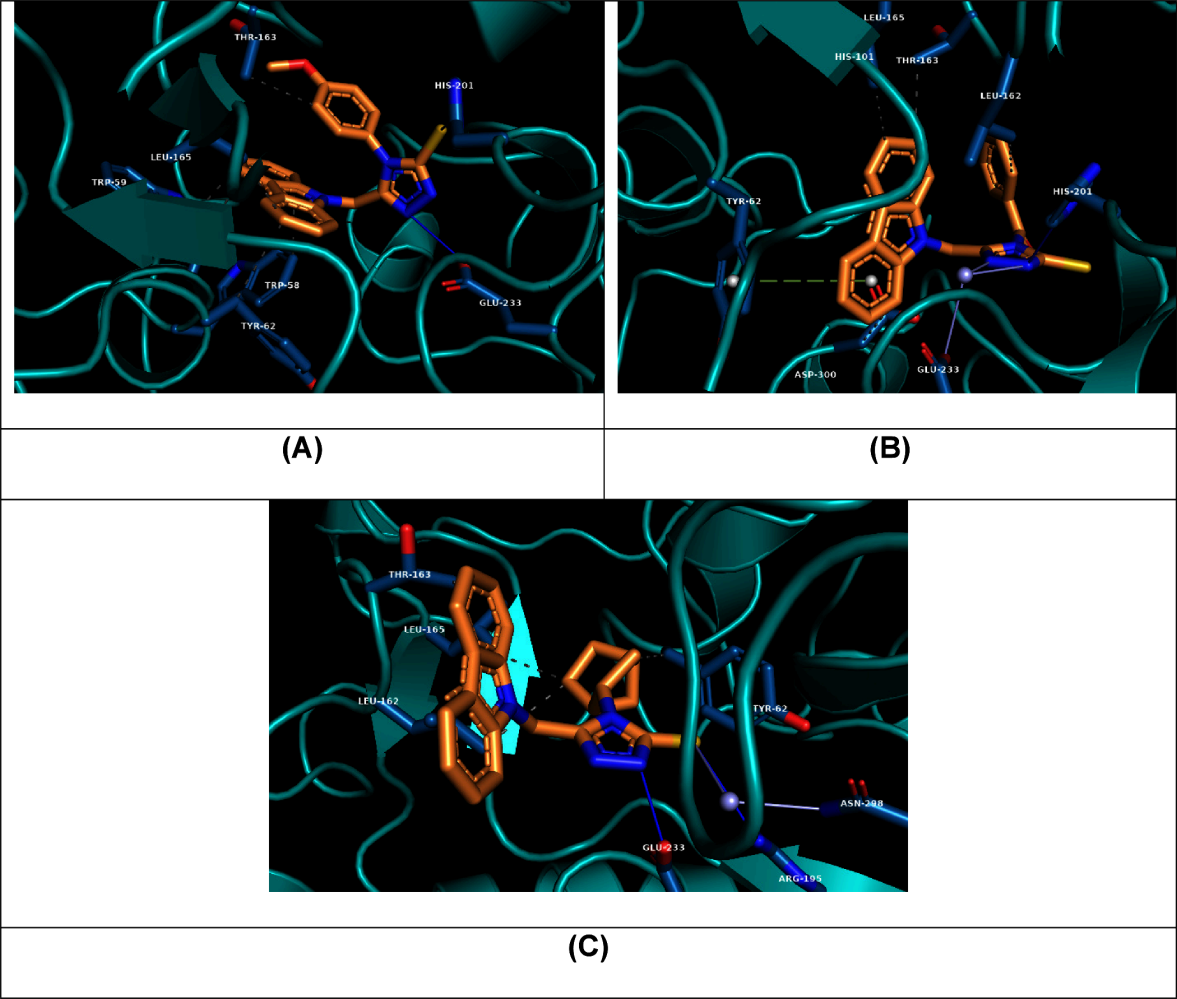
Molecular docking simulations of **C5f** (A), **C5o** (B), and **C5r** (C) structures within the binding site of the human pancreatic α-amylase protein (PDB ID: 4W93).

Given the remarkable inhibitory activity exhibited by the **C5f**, **C5o**, and **C5r** molecules against α-amylase, a molecular docking study was conducted. The highest-ranked poses for these molecules were extracted and are illustrated in Figure 4. Additionally, the interaction profiles, XP-docking scores, and predicted binding affinities are summarized in Table 2.

**Table 2:** Interaction Profiles, Docking Scores, and Binding Energies of the Tested Chemical Structures within the Binding Pocket of the Human Pancreatic α-Amylase (PDB ID: 4W93) and α-Glucosidase (PDB ID: 2ZE0) Enzymes

target	name	H. Bs	water bridges	HPHO	π-cationic	π–π stacking	docking Score (kcal/mol)	MM-GBSA (Δ*G*)
pancreatic α-amylase	**C5f**	His201, Glu233		Trp58, Trp59, Tyr62, Thr163, Leu165			–4.50	–28.7
	**C5o**	His201	Glu233	Leu162, Thr163, Leu165, Asp300	Tyr62		–4.67	–31.5
	**C5r**	Arg195, Glu233	Asn298	Tyr62, Leu162, Thr163, Leu165			–4.00	–18.6
α-glucosidase	**C5f**	Trp288, Arg300		Trp288, Arg290		Trp288	–3.7	–34.6
	**C5r**	Arg300		Leu287, Arg290, Asp297		Trp288	–2.5	–29.05

Their close docking scores and binding affinity values reflect the comparable inhibition potencies of **C5f** and **C5o**. Both molecules form shared hydrogen bonds with His201 and hydrophobic interactions with Tyr62, Thr163, and Leu165. Notably, the substitution of the methoxyphenyl moiety in **C5f** with a benzoyl moiety in **C5o** altered the structural pose, resulting in the loss of the hydrogen bond between the thione group in **C5f** and Glu233 and the formation of a new significant π-cationic interaction between the carbazole ring in **C5o** and the Tyr262 residue.

The docking simulation of the **C5r** molecule revealed the formation of two hydrogen bonds with Arg195 and Glu233 residues and multiple hydrophobic interactions primarily involving the bicyclic moiety. The larger size of the bicyclic ring in **C5r**, compared to the methoxyphenyl and benzoyl groups in **C5f** and **C5o**, respectively, is anticipated to be the primary reason for its lower affinity, as it could not be optimally accommodated within the binding site. The observed interaction profiles for our newly synthesized ligands are on par with those reported for well-known α-amylase inhibitors, such as Acarbose, curcumin, berberine,^27^ and hexadecenoic acid.^28^ Specifically, key residues Thr163 and Glu233 were consistently involved in interactions across all three docked agents, with additional interactions noted at Trp59 for **C5f**, Asp300 for **C5o**, and Leu162 for **C5r**.^27^

**Figure 5:**
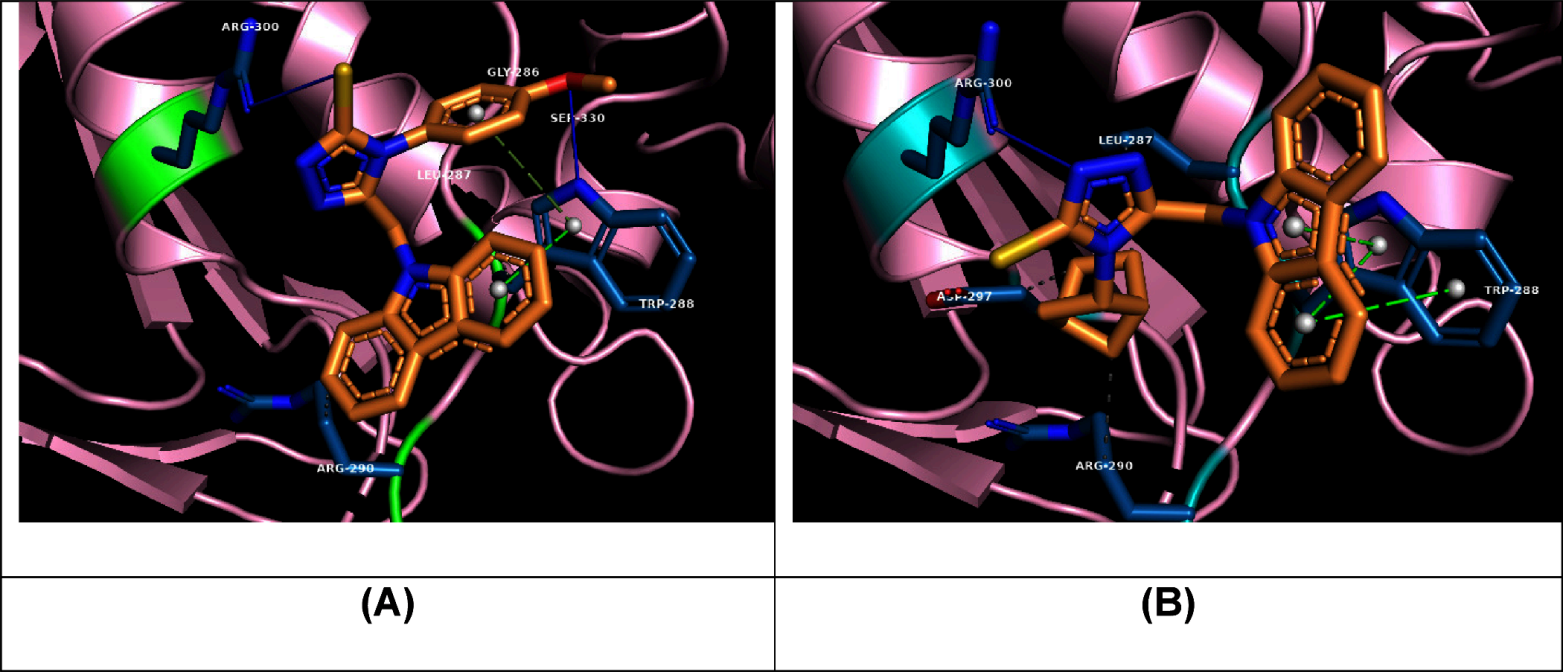
Molecular docking simulations of **C5f** (A) and **C5r** (B) structures within the binding site of the α-glucosidase (PDB ID: 2ZE0) enzyme.

A molecular docking simulation explored the medium inhibition capacity of the **C5f** and **C5r** structures against α-glucosidase enzyme, given their high inhibition potency within the newly designed C5 series. The highest-ranked poses are depicted in Figure 5, and the relevant parameters, along with the participating residues, are summarized in Table 2. Both molecules exhibited comparable interaction profiles, characterized by hydrogen bond formation with Arg300 and multiple significant hydrophobic interactions with Trp288 and Arg290. Additionally, both compounds formed a π–π stacking interaction with Trp288. The superior inhibition potency of the **C5f** molecule is likely due to the formation of an additional hydrogen bond with Trp288, which is lost when the methoxyphenyl group is replaced with a bicyclic moiety in **C5r**. The observed inhibition potency strongly correlates with the recorded docking scores and binding energy values. Furthermore, docking simulations for agents **C5f** and **C5r** revealed interactions involving multiple physical forces with key residues such as Trp287 and Leu288, consistent with findings reported in the literature.^29^

The investigation into the Structure–Activity Relationship (SAR) of the newly synthesized Carbazole-triazole-thione derivatives has elucidated several critical factors influencing their hypoglycemic efficacy. The strategic integration of the 1,2,4-triazole-3-thione ring with the carbazole moiety demonstrated significant potential in enhancing inhibitory activity against both α-amylase and α-glucosidase enzymes. Incorporating additional functional groups into the triazole ring further augmented the inhibitory potency.

Crucially, the size of the ring system plays a pivotal role in modulating the activity of the derivatives. For instance, the phenyl ring size in **C5f** has been identified as optimal, facilitating optimal accommodation within the binding site of both enzymes. Conversely, alterations in ring size, either through enlargement (e.g., **C5d**) or reduction (e.g., **C5t**), resulted in diminished enzymatic inhibition. Similarly, the introduction of bridging groups, such as methyl (**C5p**), ethyl (**C5s**), or −S–CH_2_– (**C5q**), led to a reduction in inhibitory activity, except the carbonyl bridge (**C5o**), which exhibited superior α-amylase inhibition but was less effective against α-glucosidase.

Substitution patterns on the phenyl ring also markedly influenced activity. The presence of an electron-donating group at the para position, as exemplified by **C5f**, enhanced resonance transfer to the triazole moiety, resulting in the thione functional group existing in its thiol form and thereby boosting activity. In contrast, substitutions with electron-withdrawing groups such as −CF_3_ (**C5k**), −NO_2_ (**C5e**), or −Br (**C5g**) led to a pronounced decrease in enzymatic inhibition.

Overall, these findings underscore the importance of both the size and electronic properties of the substituents in optimizing the hypoglycemic efficacy of Carbazole-triazole-thione derivatives.

#### 2.4.2 Drug-Likeness Analysis

In the early stages of drug discovery, the drug-likeness concept filters out undesirable molecules. This concept is based on the structures and characteristics of existing medications and drug candidates. By applying drug-likeness filters that consider physicochemical properties, the drug development process is significantly accelerated, allowing for the efficient identification and exclusion of compounds that are unlikely to be viable as drugs.

**Figure 6:**
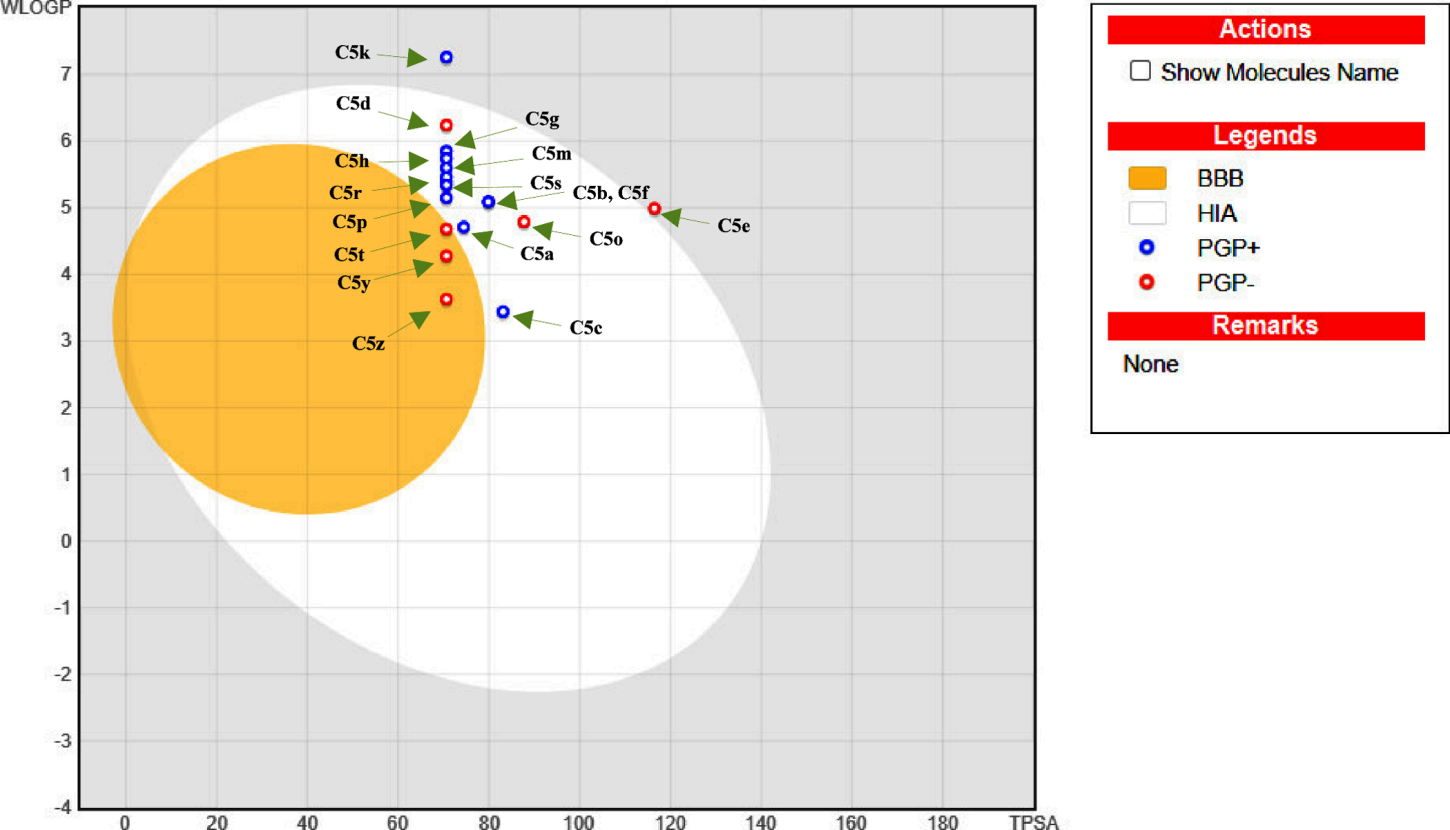
Boiled-egg plots of the **C5a**–**z** series.

To streamline the drug-likeness profiling of **C5a–z** candidates, a Boiled-egg plot was generated, illustrating the relationship between the water partition coefficient (WLOGP), also known as Wildman and Crippen LogP, and the Topological Polar Surface Area (TPSA) of the tested compounds. This analysis mainly aims to predict gastrointestinal absorption and brain penetration potential. The WLOGP parameter predicts the compound’s partitioning behavior between aqueous and lipid environments, while TPSA quantifies the polar surface area, primarily contributed by oxygen and nitrogen atoms.^30^ The plot features three distinct colors: white, yellow, and gray. The yellow zone (yolk) indicates a high likelihood of BBB (blood-brain barrier) permeability, while the white region suggests a high probability of gastrointestinal (GI) absorption. The outer gray zone Alan represents molecules with minimal absorption and no brain penetration.^31^ As shown in Figure 6, as observed, all the newly synthesized structures, except for **C5e** and **C5k**, fall within the white region, indicating that these compounds are predicted to be highly absorbed through the GI tract by passive diffusion. However, they do not possess the ability to cross the BBB, except for **C5t**, **C5y**, and **C5z**, which are outside the plot’s range and could potentially reach the brain. Furthermore, the Boiled-egg plot shows that candidates depicted as red spots (**C5d**, **C5e**, **C5o**, **C5t**, **C5y**, and **C5z**) are not P-glycoprotein (PGP) substrates, which is a positive indicator for good bioavailability, as it suggests these compounds can avoid the reflux effect.

Lipinski’s rule of five is a widely accepted guideline for assessing drug-likeness and determining whether a compound with specific biological and pharmacological properties would be orally active in humans.^32^ As shown in Table S1, we examined a broad range of physicochemical and pharmacokinetic parameters. Moreover, according to the recorded values, the physicochemical characteristics of the newly synthesized molecules adhere to Lipinski’s guidelines and remain within the specified boundaries. This study confirms that all evaluated candidates align with the properties of FDA-approved drugs, suggesting they possess potential drug-like properties with low toxicity. Consequently, the newly discovered carbazole-linked triazole-thione derivatives are considered promising candidates for drug-likeness, particularly compound **C5o**, demonstrating potential antidiabetic activity and favorable pharmacokinetic profiles. Notably, **C5o** exhibits α-amylase inhibition potency in the nanomolar range and possesses ideal pharmacokinetic characteristics, including limited penetration of the BBB and a lack of substrate activity for PGP.

## 3 Conclusions

This research significantly contributes to advancing diabetes therapeutics by introducing a series of novel carbazole-triazole-thione derivatives as prospective antidiabetic agents. Our study presents a compelling alternative by synthesizing innovative derivatives and extensively evaluating their efficacy against α-amylase and α-glucosidase enzymes, alongside their cytotoxicity against LX-2 hepatic normal cell lines.

As observed, the newly found series exhibited profound inhibitory effects on α-amylase, with seven compounds demonstrating IC_50_ values below 6.4 μM. Compounds **C5f**, **C5o**, and **C5r** emerged as exceptionally potent inhibitors. In addition, **C5f** and **C5r** exhibited moderate inhibition against α-glucosidase. The majority of evaluated compounds did not exhibit cytotoxicity against LX-2 cell lines. The assessment of compound **C5f** in a diabetes model in vivo offers useful insights into its significant antidiabetic effect. The decrease in blood glucose levels that was found approves the potential of this substance as an antidiabetic medication, which justifies further investigation. SAR analysis underscored the critical influence of the ring size fused to the 1,2,4-triazole-3-thione scaffold on biological activity, more so than its aromaticity. Compounds featuring electron-donating functional groups demonstrated enhanced efficacy against both α-amylase and α-glucosidase. Chemoinformatic analyses further clarified the binding interactions of the most active derivatives within the enzyme’s active sites, highlighting the potential of these agents to simulate the well-known hypoglycemic agents. Investigating the drug-likeness revealed that all tested compounds adhered to optimum pharmacokinetic profiles. In conclusion, the robust enzyme inhibition data and favorable drug-likeness profiles reported for these series underscore their therapeutic promise and pave the way for future clinical evaluations, offering a novel avenue for diabetes drug development.

## 4 Experimental Section

### 4.1 Chemistry

The chemical reagents and solvents utilized in this study were procured from commercial sources. The solvents used were of analytical grade. The melting points of the synthesized final products were determined using an SMP50 Automatic Melting Point apparatus, and the values were recorded without correction. During the synthesis, aluminum plates coated with silica gel were employed to monitor the reaction and control the purity of the synthesized 60 F_254_ (VWR) wed compounds. Hexane/ethyl acetate solvent systems were used in thin-layer chromatography (TLC). Camag UV lamp (254 and 366 nm) was used to observe the locations of the compounds on the TLC plates. The synthesis of the compounds was followed by purification using the Buchi Pure C-815 Automatic Flash Chromatography System, having a UV detector. Buchi EcoFlex and FlashPure silica gel columns (12, 24, 40 g) compatible with this device were used as the stationary phase, while the hexane/ethyl acetate gradient solvent system was used as the mobile phase. UPLC/MS-TOF analyses checked the compounds’ purity and provided information on the molecular weights of the target products. ^1^H- and ^13^C_APT_- NMR spectra were recorded on a Bruker DPX-400 (400 MHz) in DMSO-*d*_6_ with tetramethyl silane (TMS) as the internal standard. Chemical shifts were reported in *d* values (ppm, parts per million), and coupling constants (*J*) values were expressed in Hertz (Hz). HRMS spectra of the compounds were obtained from their solutions in methanol by positive ion (ESI+) electrospray ionization techniques using Waters LCT Premier XE UPLC/MSTOF. The stationary phase was comprised of an Aquity BEH C18 column (2.1 × 100 mm, 1.7 μM, flow rate: 0.3 mL/min), while the mobile phase consisted of a CH_3_CN/H_2_O (1–90%) gradient solvent system containing 0.1% formic acid. The ChemDraw23 software was employed for generating molecular structures, while the MestReNova 12 software was utilized for the processing of NMR FID and HRMS data, as well as the presentation of resulting spectra. In addition, NMR and HRMS spectra of all final compounds are available in the supporting data.

#### 4.1.1 Synthetic Procedure for Compounds **C5a–z**

Based on the ester (**2**) and hydrazide (**3**) compounds used as starting compounds, **C4a–z** compounds with a thiosemicarbazide functional group were synthesized as in our previous studies recorded in the literature.^33^ The appropriate thiosemicarbazide derivative (**C4a–z**) (500 mg) was stirred in 25 mL of 2N NaOH solution at 100 °C for 4 h. After this time, the mixture was transferred to 150 mL of water and brought to pH 3–4 with concentrated HCl. The precipitate that occurred was filtered and washed with water. The crude product was purified by automatic flash chromatography.

##### 4.1.1.1 5-((9H-Carbazol-9-yl)methyl)-4-phenyl-4H-1,2,4-triazole-3-thiol (**C5a**)

Purified by flash column chromatography (0% → 60% EtOAc in *n*-Hexane). Yield 93%; mp 210–212 °C. ^1^H NMR (400 MHz, DMSO-*d*_6_) δ 8.09 (d, *J* = 7.6 Hz, 2H), 7.40–7.26 (m, 7H), 7.26–7.12 (m, 4H), 5.55 (s, 2H). ^13^C NMR (101 MHz, DMSO-*d*_6_) δ 169.16, 148.86, 140.32, 133.41, 129.88, 129.74, 128.28, 126.11, 122.80, 120.54, 119.77, 109.71, 38.85. HRMS (*m*/*z*) [M + H]^+^ calcd for C_21_H_17_N_4_S: 357.1174, found: 357.1178.

##### 4.1.1.2 5-((9H-Carbazol-9-yl)methyl)-4-(3-methoxyphenyl)-2,4-dihydro-3H-1,2,4-triazole-3-thione (**C5b**)

Purified by flash column chromatography (0% → 40% EtOAc in *n*-Hexane). Yield 95%; mp 200–202 °C. ^1^H NMR (400 MHz, DMSO-*d*_6_) δ 13.94 (s, 1H), 8.08 (dt, *J* = 7.7, 1.0 Hz, 2H), 7.37–7.25 (m, 4H), 7.25–7.13 (m, 3H), 6.84 (ddd, *J* = 8.5, 2.6, 0.9 Hz, 1H), 6.73 (ddd, *J* = 7.8, 1.9, 0.9 Hz, 1H), 6.63 (t, *J* = 2.2 Hz, 1H), 5.58 (s, 2H), 3.59 (s, 3H). ^13^C NMR (101 MHz, DMSO-*d*_6_) δ 169.10, 160.06, 148.97, 140.25, 134.24, 130.43, 126.06, 122.77, 120.49, 120.18, 119.74, 115.70, 113.71, 109.65, 55.62, 38.83. HRMS (*m*/*z*) [M + H]^+^ calcd for C_22_H_19_N_4_OS: 387.1274, found: 387.1260.

##### 4.1.1.3 5-((9H-Carbazol-9-yl)methyl)-4-(3-morpholinopropyl)-2,4-dihydro-3H-1,2,4-triazole-3-thione (**C5c**)

Purified by flash column chromatography (0% → 40% EtOAc in *n*-Hexane). Yield 90%; mp 243–245 °C. ^1^H NMR (400 MHz, DMSO-*d*_6_) δ 13.78 (s, 1H), 8.20 (d, *J* = 7.7 Hz, 2H), 7.68 (d, *J* = 8.2 Hz, 2H), 7.48 (ddd, *J* = 8.3, 7.1, 1.2 Hz, 2H), 7.26 (t, *J* = 7.5 Hz, 2H), 5.86 (s, 2H), 3.93–3.79 (m, 2H), 3.46 (t, *J* = 4.6 Hz, 4H), 2.06 (s, 4H), 1.75 (t, *J* = 6.8 Hz, 2H), 1.20–1.08 (m, 2H). ^13^C NMR (101 MHz, DMSO-*d*_6_) δ 167.81, 148.96, 140.43, 126.52, 122.99, 120.98, 120.09, 109.93, 66.52, 55.01, 53.34, 42.62, 38.70, 24.13. HRMS (*m*/*z*) [M + H]^+^ calcd for C_22_H_26_N_5_OS: 408.1853, found: 408.1846.

##### 4.1.1.4 5-((9H-Carbazol-9-yl)methyl)-4-(naphthalen-1-yl)-2,4-dihydro-3H-1,2,4-triazole-3-thione (**C5d**)

Purified by flash column chromatography (0% → 60% EtOAc in *n*-Hexane). Yield 88%; mp 277–279 °C. ^1^H NMR (400 MHz, DMSO-*d*_6_) δ 14.13 (s, 1H), 7.92–7.70 (m, 4H), 7.45–7.33 (m, 3H), 7.23–7.13 (m, 2H), 7.07 (td, *J* = 7.2, 3.2 Hz, 3H), 7.00 (d, *J* = 8.1 Hz, 2H), 6.72 (d, *J* = 8.5 Hz, 1H), 5.58–5.38 (m, 2H). ^13^C NMR (101 MHz, DMSO-*d*_6_) δ 169.83, 149.69, 139.89, 134.00, 130.46, 129.57, 129.30, 128.45, 127.16, 127.03, 126.67, 125.85, 125.72, 122.62, 121.45, 120.25, 119.53, 109.06, 38.68. HRMS (*m*/*z*) [M + H]^+^ calcd for C_25_H_19_N_4_S: 407.1325, found: 407.1327.

##### 4.1.1.5 5-((9H-Carbazol-9-yl)methyl)-4-(4-nitrophenyl)-2,4-dihydro-3H-1,2,4-triazole-3-thione (**C5e**)

Purified by flash column chromatography (0% → 40% EtOAc in *n*-Hexane). Yield 92%; mp 288–290 °C. ^1^H NMR (400 MHz, DMSO-*d*_6_) δ 14.10 (s, 1H), 8.05 (d, *J* = 7.7 Hz, 2H), 8.00–7.88 (m, 2H), 7.38–7.26 (m, 4H), 7.26–7.20 (m, 2H), 7.15 (ddd, *J* = 8.0, 6.5, 1.7 Hz, 2H), 5.68 (s, 2H). ^13^C NMR (101 MHz, DMSO-*d*_6_) δ 168.96, 148.88, 147.69, 140.00, 138.54, 129.44, 126.05, 124.71, 122.77, 120.59, 119.87, 109.71, 38.95. HRMS (*m*/*z*) [M + H]^+^ calcd for C_21_H_16_N_5_O_2_S: 402.1019, found: 402.0691.

##### 4.1.1.6 5-((9H-Carbazol-9-yl)methyl)-4-(4-methoxyphenyl)-2,4-dihydro-3H-1,2,4-triazole-3-thione (**C5f**)

Purified by flash column chromatography (0% → 60% EtOAc in *n*-Hexane). Yield 93%; mp 198–200 °C. ^1^H NMR (400 MHz, DMSO-*d*_6_) δ 13.86 (s, 1H), 8.10 (d, *J* = 7.7 Hz, 2H), 7.31 (dt, *J* = 17.1, 7.9 Hz, 4H), 7.18 (t, *J* = 7.3 Hz, 2H), 7.04 (d, *J* = 8.8 Hz, 2H), 6.89–6.75 (m, 2H), 5.54 (s, 2H), 3.73 (s, 3H). ^13^C NMR (101 MHz, DMSO-*d*_6_) δ 169.39, 160.24, 149.10, 140.29, 129.39, 126.06, 125.82, 122.79, 120.54, 119.71, 114.90, 109.73, 55.84, 38.81. HRMS (*m*/*z*) [M + H]^+^ calcd for C_22_H_19_N_4_OS: 387.1274, found: 387.1272.

##### 4.1.1.7 5-((9H-Carbazol-9-yl)methyl)-4-(4-bromophenyl)-2,4-dihydro-3H-1,2,4-triazole-3-thione (**C5g**)

Purified by flash column chromatography (0% → 60% EtOAc in *n*-Hexane). Yield 83%; mp 177–178 °C. ^1^H NMR (400 MHz, DMSO-*d*_6_) δ 14.99–13.16 (m, 1H), 8.09 (d, *J* = 7.7 Hz, 2H), 7.52–7.38 (m, 2H), 7.38–7.24 (m, 4H), 7.18 (ddd, *J* = 7.9, 6.8, 1.3 Hz, 2H), 7.07–6.97 (m, 2H), 5.58 (s, 2H). ^13^C NMR (101 MHz, DMSO-*d*_6_) δ 168.86, 148.67, 140.19, 132.84, 132.60, 130.28, 126.03, 123.16, 122.74, 120.56, 119.75, 109.74, 38.88. HRMS (*m*/*z*) [M + H]^+^ calcd for C_22_H_26_N_5_OS: 435.0274, found: 435.0254.

##### 4.1.1.8 5-((9H-Carbazol-9-yl)methyl)-4-(4-chlorophenyl)-2,4-dihydro-3H-1,2,4-triazole-3-thione (**C5h**)

Purified by flash column chromatography (0% → 60% EtOAc in *n*-Hexane). Yield 87%; mp 210–211 °C. ^1^H NMR (400 MHz, DMSO-*d*_6_) δ 8.07 (d, *J* = 7.7 Hz, 2H), 7.37–7.22 (m, 6H), 7.18 (ddd, *J* = 7.9, 6.8, 1.2 Hz, 2H), 7.07–6.99 (m, 2H), 5.56 (s, 2H). ^13^C NMR (101 MHz, DMSO-*d*_6_) δ 168.73, 148.62, 140.21, 134.33, 132.71, 130.01, 129.97, 129.57, 129.53, 126.00, 125.98, 122.73, 120.53, 119.70, 119.68, 109.78, 109.74, 40.59, 40.38, 40.18, 39.97, 39.76, 39.55, 39.34, 38.95. HRMS (*m*/*z*) [M + H]^+^ calcd for C_21_H_16_ClN_4_S: 359.1063, found: 359.1058.

##### 4.1.1.9 5-((9H-Carbazol-9-yl)methyl)-4-(4-(trifluoromethyl)phenyl)-2,4-dihydro-3H-1,2,4-triazole-3-thione (**C5k**)

Purified by flash column chromatography (0% → 60% EtOAc in *n*-Hexane). Yield 79%; mp 177–179 °C. ^1^H NMR (400 MHz, DMSO) δ 10.62 (s, 1H), 8.25–8.13 (m, 2H), 7.82–7.70 (m, 4H), 7.70–7.60 (m, 2H), 7.56–7.46 (m, 2H), 7.32–7.21 (m, 2H), 6.02 (s, 2H). ^13^C NMR (101 MHz, DMSO) δ 164.55, 157.24, 144.01, 140.12, 126.88, 126.84, 126.49, 123.11, 124.75 (q, ^1^*J*_CF_ = 270.9 Hz), 122.16 (q, ^2^*J*_CF_ = 32.1 Hz), 120.94, 120.14, 117.62, 110.10. ^19^F NMR (471 MHz, DMSO) δ −61.26. HRMS (*m*/*z*) [M + H]^+^ calcd for C_22_H_15_F_3_N_4_S: 425.1042, found: 425.1066.

##### 4.1.1.10 5-((9H-Carbazol-9-yl)methyl)-4-cyclohexyl-2,4-dihydro-3H-1,2,4-triazole-3-thione (**C5m**)

Purified by flash column chromatography (0% → 40% EtOAc in *n*-Hexane). Yield 92%; mp 222–223 °C. ^1^H NMR (400 MHz, DMSO-*d*_6_) δ 13.51 (s, 1H), 8.18 (d, *J* = 7.7 Hz, 2H), 7.63 (d, *J* = 8.3 Hz, 2H), 7.47 (t, *J* = 7.7 Hz, 2H), 7.25 (t, *J* = 7.5 Hz, 2H), 5.89 (s, 2H), 4.24 (s, 1H), 2.44 (d, *J* = 13.2 Hz, 2H), 1.63 (d, *J* = 12.7 Hz, 2H), 1.57–1.22 (m, 4H), 1.14–0.79 (m, 4H). ^13^C NMR (101 MHz, DMSO-*d*_6_) δ 167.09, 148.99, 140.49, 126.37, 122.96, 120.89, 119.97, 110.07, 56.76, 40.59, 40.39, 40.18, 39.97, 39.76, 39.55, 39.34, 39.18, 28.71, 25.53, 24.78. HRMS (*m*/*z*) [M + H]^+^ calcd for C_21_H_23_N_4_S: 363.1638, found: 363.1674.

##### 4.1.1.11 5-((9H-Carbazol-9-yl)methyl)-4-(p-tolyl)-2,4-dihydro-3H-1,2,4-triazole-3-thione (**C5n**)

Purified by flash column chromatography (0% → 40% EtOAc in *n*-Hexane). Yield 95%; mp 214–216 °C. ^1^H NMR (400 MHz, DMSO-*d*_6_) δ 13.81 (s, 1H), 8.08 (d, *J* = 7.7 Hz, 2H), 7.33 (t, *J* = 7.6 Hz, 2H), 7.25 (d, *J* = 8.2 Hz, 2H), 7.18 (t, *J* = 7.4 Hz, 2H), 7.09 (d, *J* = 7.9 Hz, 2H), 6.99 (d, *J* = 7.9 Hz, 2H), 5.53 (s, 2H), 2.27 (s, 3H). ^13^C NMR (101 MHz, DMSO-*d*_6_) δ 169.16, 148.92, 140.26, 139.66, 130.73, 130.22, 127.90, 126.04, 122.80, 120.53, 119.71, 109.74, 38.79, 21.20. HRMS (*m*/*z*) [M + H]^+^ calcd for C_22_H_19_N_4_S: 371.1325, found: 371.1357.

##### 4.1.1.12 (3-((9H-Carbazol-9-yl)methyl)-5-thioxo-1,5-dihydro-4H-1,2,4-triazol-4-yl)(phenyl)methanone (**C5o**)

Purified by flash column chromatography (0% → 40% EtOAc in *n*-Hexane). Yield 89%; mp 255–257 °C ^1^H NMR (400 MHz, DMSO-*d*_6_) δ 13.00 (s, 1H), 8.20 (d, J = 7.7 Hz, 2H), 8.07–7.95 (m, 2H), 7.79 (d, J = 8.2 Hz, 2H), 7.69–7.57 (m, 1H), 7.52 (t, J = 7.8 Hz, 4H), 7.28 (t, J = 7.5 Hz, 2H), 6.11 (s, 2H). ^13^C NMR (101 MHz, DMSO-*d*_6_) δ 175.19, 160.95, 140.08, 133.47, 129.09, 128.78, 126.54, 123.07, 120.95, 120.13, 110.07, 41.36. HRMS (*m*/*z*) [M + H]^+^ calcd for C_22_H_17_N_4_OS: 385.1176, found: 385.1110.

##### 4.1.1.13 5-((9H-Carbazol-9-yl)methyl)-4-benzyl-2,4-dihydro-3H-1,2,4-triazole-3-thione (**C5p**)

Purified by flash column chromatography (0% → 40% EtOAc in *n*-Hexane). Yield 82%; mp 270–271 °C. ^1^H NMR (400 MHz, DMSO-*d*_6_) δ 8.06 (d, *J* = 7.7 Hz, 2H), 7.36 (t, *J* = 7.7 Hz, 2H), 7.29–7.12 (m, 7H), 7.02 (d, *J* = 8.0 Hz, 2H), 5.62 (d, *J* = 5.3 Hz, 2H), 5.35 (s, 2H). ^13^C NMR (101 MHz, DMSO-*d*_6_) δ 168.64, 149.25, 140.44, 135.24, 128.89, 127.97, 126.82, 126.22, 122.91, 120.73, 119.80, 109.38, 46.43, 38.71. HRMS (*m*/*z*) [M + H]^+^ calcd for C_22_H_19_N_4_S: 371.1325, found: 371.1369.

##### 4.1.1.14 5-((9H-Carbazol-9-yl)methyl)-4-(bicyclo[2.2.1]heptan-2-yl)-2,4-dihydro-3H-1,2,4-triazole-3-thione (**C5r**)

Purified by flash column chromatography (0% → 40% EtOAc in *n*-Hexane). Yield 86%; mp 206–208 °C. ^1^H NMR (400 MHz, DMSO-*d*_6_) δ 13.49 (s, 1H), 8.19 (dt, *J* = 7.7, 1.0 Hz, 2H), 7.62 (d, *J* = 8.2 Hz, 2H), 7.45 (ddd, *J* = 8.3, 7.2, 1.2 Hz, 2H), 7.24 (td, *J* = 7.5, 0.9 Hz, 2H), 5.87 (s, 2H), 4.31 (dd, *J* = 8.7, 5.8 Hz, 1H), 2.68–2.59 (m, 1H), 2.45 (d, *J* = 4.0 Hz, 1H), 2.30 (s, 1H), 1.56 (ddd, *J* = 11.5, 8.7, 2.1 Hz, 1H), 1.35 (ddt, *J* = 12.3, 7.9, 3.8 Hz, 2H), 1.20 (d, *J* = 9.8 Hz, 2H), 1.00 (td, *J* = 10.0, 9.4, 2.7 Hz, 2H). ^13^C NMR (101 MHz, DMSO-*d*_6_) δ 167.63, 149.20, 140.74, 126.32, 122.95, 120.80, 119.88, 109.99, 61.42, 41.74, 38.18, 36.24, 36.21, 28.90, 27.78. HRMS (*m*/*z*) [M + H]^+^ calcd for C_22_H_23_N_4_S: 375.1638, found: 375.1633.

##### 4.1.1.15 5-((9H-Carbazol-9-yl)methyl)-4-phenethyl-2,4-dihydro-3H-1,2,4-triazole-3-thione (**C5s**)

Purified by flash column chromatography (0% → 40% EtOAc in *n*-Hexane). Yield 92%; mp 243–245 °C. ^1^H NMR (400 MHz, DMSO-*d*_6_) δ 13.71 (s, 1H), 8.20 (d, *J* = 7.8 Hz, 2H), 7.47 (dt, *J* = 15.1, 8.0 Hz, 4H), 7.34–7.20 (m, 5H), 6.81 (dd, *J* = 6.6, 2.8 Hz, 2H), 5.60 (s, 2H), 4.15–3.96 (m, 2H), 2.50–2.44 (m, 2H). ^13^C NMR (101 MHz, DMSO-*d*_6_) δ 167.81, 148.88, 140.48, 137.78, 129.20, 129.03, 127.20, 126.56, 122.92, 121.01, 120.08, 109.86, 45.82, 40.35, 40.14, 39.93, 39.73, 39.52, 38.59, 33.17. HRMS (*m*/*z*) [M + H]^+^ calcd for C_23_H_21_N_4_S: 385.1481, found: 385.1529.

##### 4.1.1.16 5-((9H-Carbazol-9-yl)methyl)-4-isopropyl-2,4-dihydro-3H-1,2,4-triazole-3-thione (**C5t**)

Purified by flash column chromatography (0% → 80% EtOAc in *n*-Hexane). Yield 87%; mp 188–190 °C. ^1^H NMR (400 MHz, DMSO-*d*_6_) δ 13.44 (s, 1H), 8.24–8.13 (m, 2H), 7.58 (d, *J* = 8.2 Hz, 2H), 7.45 (ddd, *J* = 8.3, 7.1, 1.2 Hz, 2H), 7.31–7.18 (m, 2H), 5.87 (s, 2H), 4.91 (p, *J* = 6.9 Hz, 1H), 1.53 (d, *J* = 7.0 Hz, 6H). ^13^C NMR (101 MHz, DMSO-*d*_6_) δ 167.16, 148.78, 140.72, 126.33, 122.94, 120.78, 119.87, 109.98, 48.86, 39.34, 39.22, 19.96. HRMS (*m*/*z*) [M + H]^+^ calcd for C_18_H_19_N_4_S: 323.1325, found: 323.1391.

##### 4.1.1.17 5-((9H-Carbazol-9-yl)methyl)-4-allyl-2,4-dihydro-3H-1,2,4-triazole-3-thione (**C5y**)

Purified by flash column chromatography (0% → 80% EtOAc in *n*-Hexane). Yield 93%; mp 185–186 °C. ^1^H NMR (400 MHz, DMSO-*d*_6_) δ 13.65 (s, 1H), 8.17 (d, *J* = 7.7 Hz, 2H), 7.57 (d, *J* = 8.2 Hz, 2H), 7.46 (ddd, *J* = 8.3, 7.1, 1.2 Hz, 2H), 7.25 (t, *J* = 7.4 Hz, 2H), 5.75 (s, 2H), 5.61 (ddt, *J* = 17.3, 10.3, 5.1 Hz, 1H), 4.97 (dd, *J* = 10.4, 1.5 Hz, 1H), 4.86–4.75 (m, 1H), 4.67 (dt, *J* = 5.4, 1.8 Hz, 2H). ^13^C NMR (101 MHz, DMSO-*d*_6_) δ 168.12, 148.99, 140.63, 131.25, 126.38, 123.00, 120.85, 119.95, 117.64, 109.77, 45.60, 38.67. HRMS (*m*/*z*) [M + H]^+^ calcd for C_18_H_17_N_4_S: 321.1168, found: 321.1219.

##### 4.1.1.18 5-((9H-Carbazol-9-yl)methyl)-4-methyl-2,4-dihydro-3H-1,2,4-triazole-3-thione (**C5z**)

Purified by flash column chromatography (0%→80% EtOAc in *n*-Hexane). Yield 89%; mp 203–205 °C. ^1^H NMR (400 MHz, DMSO-*d*_6_) δ 13.52 (s, 1H), 8.18 (d, *J* = 7.7 Hz, 2H), 7.66 (d, *J* = 8.2 Hz, 2H), 7.46 (t, *J* = 7.6 Hz, 2H), 7.25 (t, *J* = 7.4 Hz, 2H), 5.81 (s, 2H), 3.44 (s, 3H). ^13^C NMR (101 MHz, DMSO-*d*_6_) δ 168.02, 149.28, 140.74, 126.36, 122.90, 120.82, 119.93, 110.05, 38.72, 30.46. HRMS (*m*/*z*) [M + H]^+^ calcd for C_16_H_14_N_4_S: 295.1012, found: 295.1082.

### 4.2 Biological Methods

#### 4.2.1 α-Amylase Inhibitory Activity

The α-amylase inhibitory investigations were established following the Wickramaratne et al. procedure^34^ with minor changes. The used solutions with concentrations of 200, 100, 50, 10, 1, and 0.5 μM were produced from our synthesized compounds, diluting them with a buffer of Na_2_HPO_4_/NaH_2_PO_4_ (0.02 M), NaCl (0.006 M) at pH 6.9 and then brined up to 10 mL using a 10 mL volumetric flask. The acarbose antidiabetic agent was considered a positive control and was established following the same previous steps utilized for the synthesized compounds. As percent inhibition, the enzyme inhibitory potential was expressed, and the following equation was utilized to estimate the IC_50_ dose for the samples.^35^
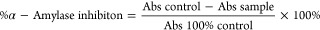


#### 4.2.2 α-Glucosidase Activity

The inhibition of α-glucosidase activity by the synthesized molecules was measured. The α-glucosidase activity was measured using 200, 100, 50, 10, and 1 μM of the synthesized compounds. Each concentration was recorded using 1, 3, 6, 9, and 12 mm PNPG. The inhibitory pattern was assessed using a Lineweaver–Burk plot.^36^ The α-glucosidase inhibitory activity is expressed as percentages of inhibition, which were calculated using the following formula:
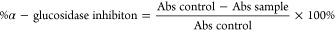


#### 4.2.3 Cell Culture Cytotoxicity Assay

Human hepatic stellate (LX-2) cells obtained from ATCC in Rockville, MD, USA were grown in RPMI-1640 media. The media was supplemented with a 1% combination of Streptomycin and Penicillin, 1% l-glutamine, and 10% fetal bovine serum. The cells were cultivated at a temperature of 37 °C in a controlled environment with a humidity level and an atmosphere containing 5% carbon dioxide (CO_2_). The cells were distributed at a density of 2.6 × 10^4^ cells per well in a 96-well plate. Following a 48-h period, cells were incubated with different doses (300, 100, and 50 μM) of each chemical for 24 h. 5-Fluorouracil (5-Fu) was employed as a positive control at an equivalent concentration. Cell viability was evaluated using the CellTilter 96 Aqueous One Solution Cell Proliferation (MTS) Assay, following the directions provided by the manufacturer (Promega Corporation, Madison, WI). After the treatment, 20 μL of MTS solution was added to each well, along with 100 μL of medium. The mixture was then incubated at a temperature of 37 °C for a duration of 2 h. The absorbance measurement was conducted at a wavelength of 490 nm.^37^

#### 4.2.4 In Vivo Evaluation of the Antidiabetic Effect

##### 4.2.4.1 Animal

The investigations conducted on mice adhered strictly to the protocols established by the Association for Assessment and Accreditation of Laboratory Animal Care International. The Institutional Review Board of the Animal House and Use Committee at An-Najah National University granted full ethical approval for the study (approval number: Med. September. 2022/2A). The study included 21 healthy adult mice with a weight range of 20.5–26 g and an 8–10-week age range. The mice were housed in groups of four in the animal facility. During the preliminary phase, the mice were allowed to adjust to the regulated temperature conditions for a period of 7 days, ensuring that the temperature remained steady at 25 ± 2 °C.^38^

##### 4.2.4.2 Animal Groups

The experimental groups comprised a control group (Group I) that did not receive a streptozotocin (STZ) injection and two diabetic groups (Groups II and III) that were given STZ injections. Group II functioned as the control group for diabetes, and Group III was the diabetes group treated with Compound **C5f** through intraperitoneal injection.

#### 4.2.5 Induction of Experimental Diabetes and Drug Administration

The experiment entailed inducing diabetes intraperitoneally by administering streptozotocin (STZ) (i.p.). The STZ compound was dissolved in a cold citrate buffer with a concentration of 0.10 M and a pH value of 4.5. Every mouse was administered an injection of STZ 40 mg/kg. To determine the development of diabetes, the mice’s fasting blood glucose levels were evaluated 4 days following the injection of STZ. An elevation in the fasting blood glucose level at this stage was utilized to indicate the successful initiation of the DM disease. On the 10th day, after receiving seven injections of STZ, all mice were deprived of food for 6 h. Subsequently, a blood glucose test was conducted using a blood sample from the tail vein.^38^ At the end of the diabetes induction period, the mice in groups II and III that were diagnosed with diabetes, then group III were treated daily with an i.p. injection of compound **C5f** at a dose of 10 mg/kg for 5 subsequent days. Meanwhile, the remaining groups, including Group I (nondiabetic) and Group II (diabetic), were maintained as control groups.

### 4.3 Chemoinformatics Studies

#### 4.3.1 Molecular Docking Studies

The molecular docking studies were rigorously conducted to determine the binding orientation of carbazole-triazole-thione derivatives within the active sites of target proteins, thereby clarifying the biological mechanisms through detailed ligand–receptor interaction analysis. This research sequence began with a methodical protocol that included the ligand’s specification and optimization, the receptor’s preparation, and the construction of the computational grid. Following these steps, XP-Glide docking simulations were performed.

#### 4.3.2 Ligand Drawing and Preparation

The initial phase involved the graphical modeling of the selected ligands through the Maestro Graphical User Interface (version 13.7, Schrödinger Suite). Subsequently, a comprehensive preparatory procedure was conducted using LigPrep, wherein the ligands were subjected to optimization in accordance with the OPLS2005 force field parameters. Additionally, the ligands were meticulously adjusted to their relevant protonation states at a physiological pH of 7.0 ± 2.0, ensuring an accurate representation of their biological conditions.^33,39^

#### 4.3.3 Protein Preparation and Grid Generation

To investigate the antidiabetic potential of the newly designed carbazole-triazole-thione derivatives, the crystalline structures of Human pancreatic α-amylase complexed with montbretin A (PDB ID: 4W93, Resolution: 1.35 Å) and α-glucosidase GSJ (PDB ID: 2ZE0, Resolution: 2.00 Å) were selected to represent the respective enzymes. The structures were obtained from the Protein Data Bank (https://www.rcsb.org/) and meticulously prepared using the Schrödinger Maestro Protein Preparation Wizard (version 13.7). This preparatory phase encompassed several critical steps: the incorporation of hydrogen atoms, reconstruction of absent side chains utilizing Prime, precise assignment of bond orders and charge states, and the elimination of water molecules situated more than 5 Å from heteroatoms. The protein structures underwent energy minimization following these adjustments, employing the OPLS_2005 force field. The OPLS2005 force field was employed to optimize our newly designed carbazole-linked 1,2,4-triazole-thione derivatives due to its reliability in modeling organic and biological molecules and its validated performance against experimental data. Moreover, the docking scores obtained correlate well with the observed biological activity, highlighting the precision and potential of this minimization tool.^40^ Subsequently, receptor grids were meticulously generated with dimensions set to 20 Å × 20 Å × 20 Å for both crystallographic structures to facilitate accurate docking simulations. For the 4W93 structure, the active site was determined based on its native ligand, montbrentin A. In contrast, since the 2ZE0 structure does not have a native ligand, the active site was identified by selecting the residues that constitute the binding site, as outlined in the literature: specifically, Ala200, Leu287, Trp288, Arg300, and Arg415.^41^

#### 4.3.4 Glide Extraprecision (XP) Ligand Docking

Utilizing the sophisticated capabilities of Schrödinger-Maestro’s Glide tool (version 13.7), an exhaustive XP Glide docking procedure was conducted with precision. Particular emphasis was placed on fine-tuning the partial charge cutoffs and van der Waals scaling factors, meticulously calibrated to 0.15 and 0.80 for the ligand atoms, respectively.^42^ The final scoring was executed upon achieving energy minimization, leveraging the Glide score for an immediate assessment. The conformation with the most favorable Glide score was designated as the optimal binding pose for each docked ligand. These preferred configurations were subsequently subjected to a detailed analysis of their binding profiles via the PLIP server. Additionally, the finalized results were visually rendered using the PyMOL application (version 2.5.2) to facilitate comprehensive structural interpretation.^43^

#### 4.3.5 Free Energy Calculations Using Prime MM-GBSA

The binding energies of the docked ligands within the target proteins’ binding sites were computed using the Prime Molecular Mechanics—Generalized Born Surface Area (Prime MM-GBSA) module, integrated into the Maestro (version 13.7) interface. This computation employed the XP-Glide pose viewer files obtained for the docked ligands, with the calculations conducted using the VSGB 2.0 solvent model (2021) and the OPLS4 force field. The total free energies for each ligand–receptor complex were systematically recorded. The variation in free energy was subsequently determined through the application of the relevant thermodynamic equations:
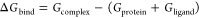
1In this equation, Δ*G*_bind_ signifies the binding energy of the ligand, with *G*_complex_, *G*_protein_, and *G*_ligand_ representing the minimized energies of the protein–ligand complex, the unbound protein, and the unbound ligand, respectively.^44^

#### 4.3.6 Drug-Likeness Prediction

To evaluate the drug-likeness of the newly synthesized carbazole-triazole-thione derivatives, a detailed analysis of ADME (absorption, distribution, metabolism, excretion) properties was performed using both the SwissADME online platform^45^ and the QikProp module integrated within the Schrödinger Maestro suite.^46^ This evaluation elucidated a range of critical parameters pertinent to the pharmacokinetic profiles of the tested compounds. The ADME-T analysis aimed to rigorously assess the extent to which these novel derivatives conform to the pharmacokinetic and safety profiles typical of FDA-approved drugs, thereby offering a robust prediction of their potential therapeutic viability.

### 4.4 Statistical Analysis

All data were reported as mean values with standard deviations (SD), and statistical significance was established with a *p*-value threshold of less than 0.05. The analysis was performed using an unpaired *t* test to evaluate the differences between groups and determine the significance of the findings.

## Supporting Information Available

^1^H- and ^13^C_APT_-NMR spectra and the synthesized compounds’ HRMS spectra and provides the ADME-T analysis of the **C5a–z** series (PDF)
