# Supplementary material for: Next-Generation Carbazole-Linked 1,2,4-Triazole-Thione Derivatives: Strategic Design, Synthesis, Molecular Docking, and Evaluation of Antidiabetic Potential
Source: ACS Omega. 2024 Dec 25;10(1):848–61. doi: 10.1021/acsomega.4c07896 (PMC11739978; doi:10.1021/acsomega.4c07896)
Supplement: Supplementary file 1 — ao4c07896_si_001.pdf [file ao4c07896_si_001.pdf]

Supporting Information

Next-Generation Carbazole-Linked 1,2,4-Triazole-Thione Derivatives: Strategic Design, Synthesis, Molecular Docking, and Evaluation of Antidiabetic Potential

İrfan Çapan [1,2]*, Mohammed Hawash [3], Mohammed T. Qaoud [4], Nidal Jaradat [3]

[1] Department of Pharmaceutical Basic Sciences, Faculty of Pharmacy, Gazi University, 06330 Ankara, Türkiye.

[2] Sente Kimya Research and Development Inc., 06200 Ankara, Türkiye.

[3] Department of Pharmacy, Faculty of Medicine and Health Sciences, An-Najah National University, Nablus, Palestine.

[4] Department of Pharmacy, Faculty of Pharmacy, Cyprus International University, Northern Cyprus, Mersin 10, 99258 Nicosia, Türkiye.

***Corresponding author:**

İrfan Çapan; Gazi University, Faculty of Pharmacy, Department of Pharmaceutical Basic Sciences, 06560 Ankara Turkey, Phone: +90 312 202 3027 Fax: +90 312 202 80 43,

E-mail: irfancapan@gazi.edu.tr

Spectrums of Compound C5a

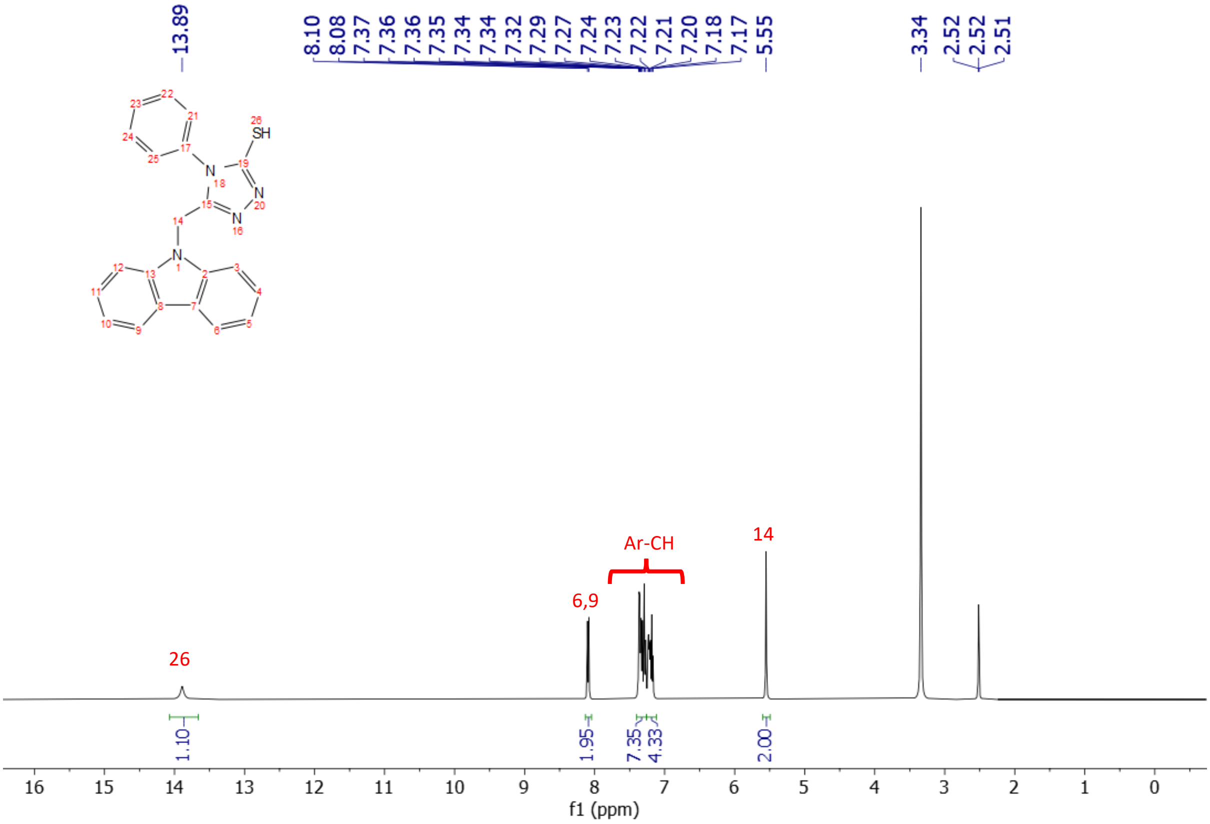

Figure S1: ^{1}H-NMR spectrum of Compound C5a

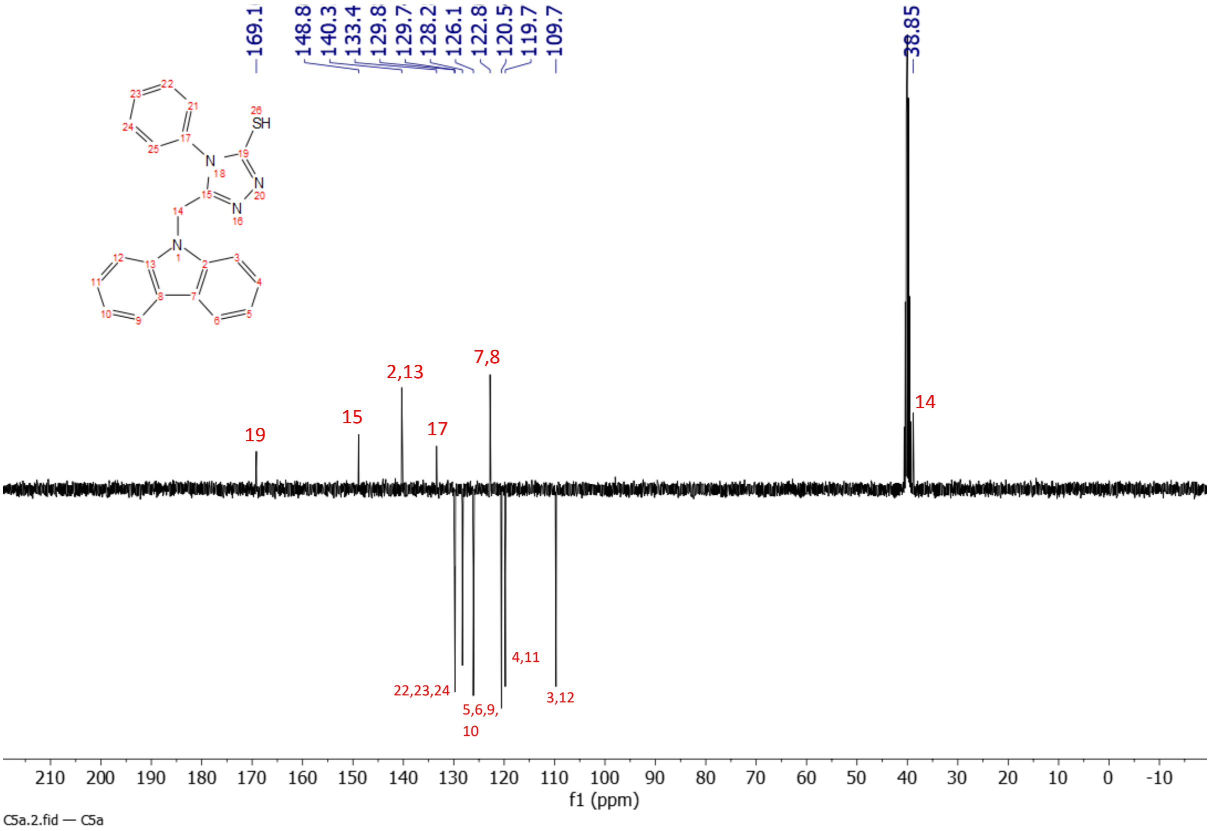

Figure S2: $^{13}C_{APT}$-NMR spectrum of Compound C5a

Spectrums of Compound C5b

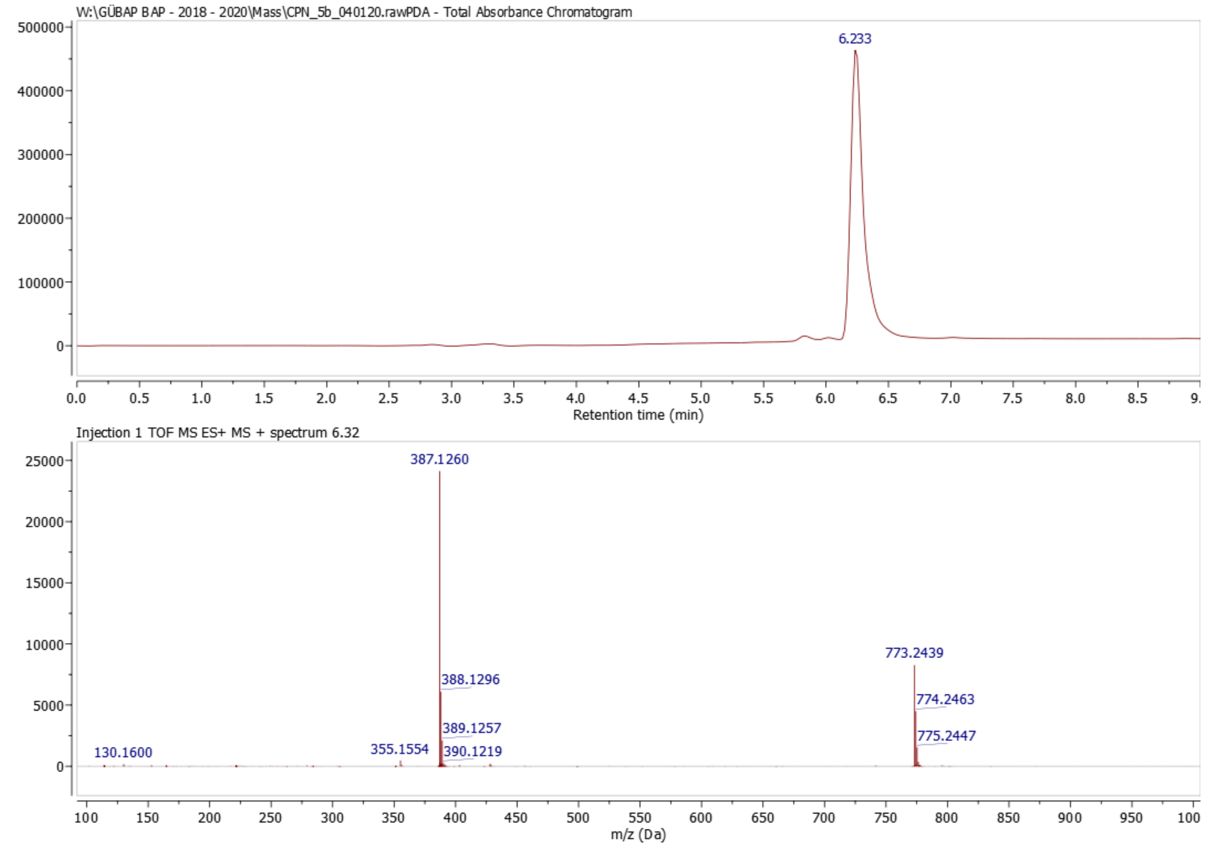

Figure S3: HRMS Spectrum of Compound C5b

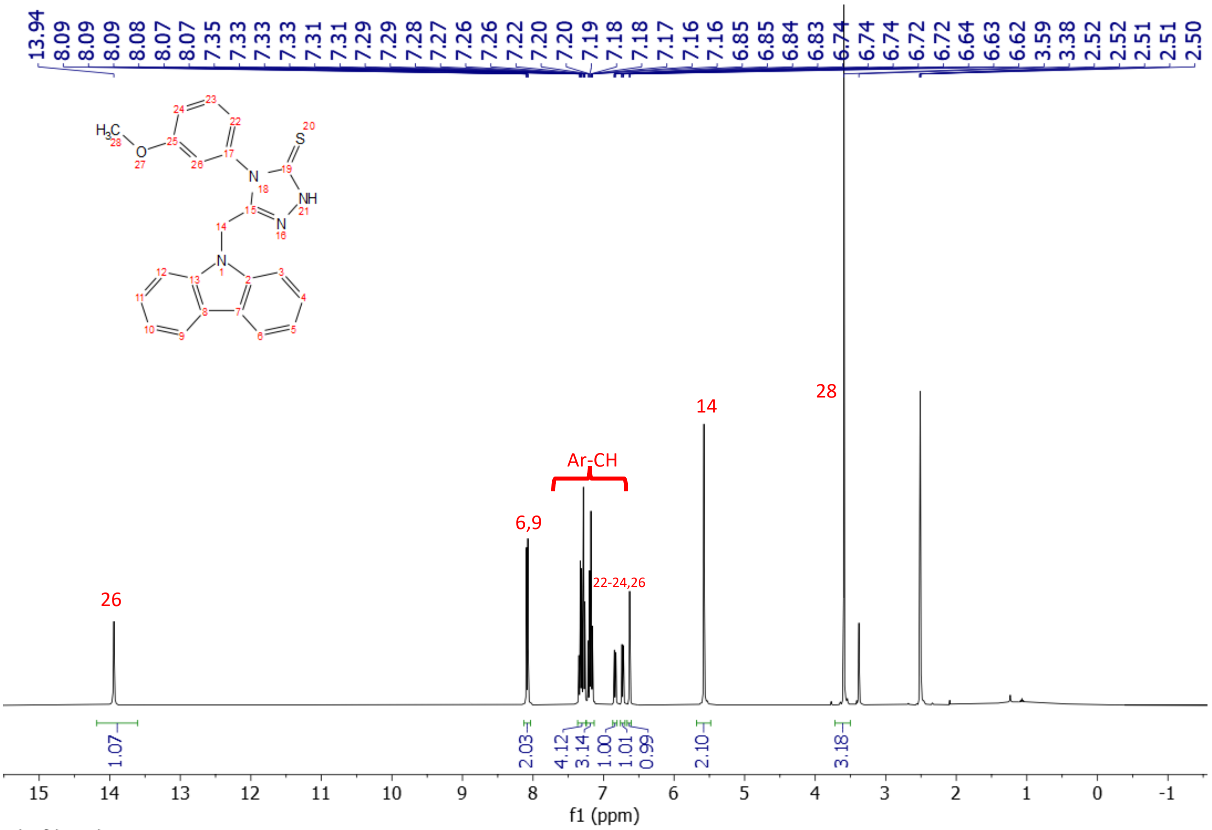

Figure S4: ¹H-NMR spectrum of Compound C5b

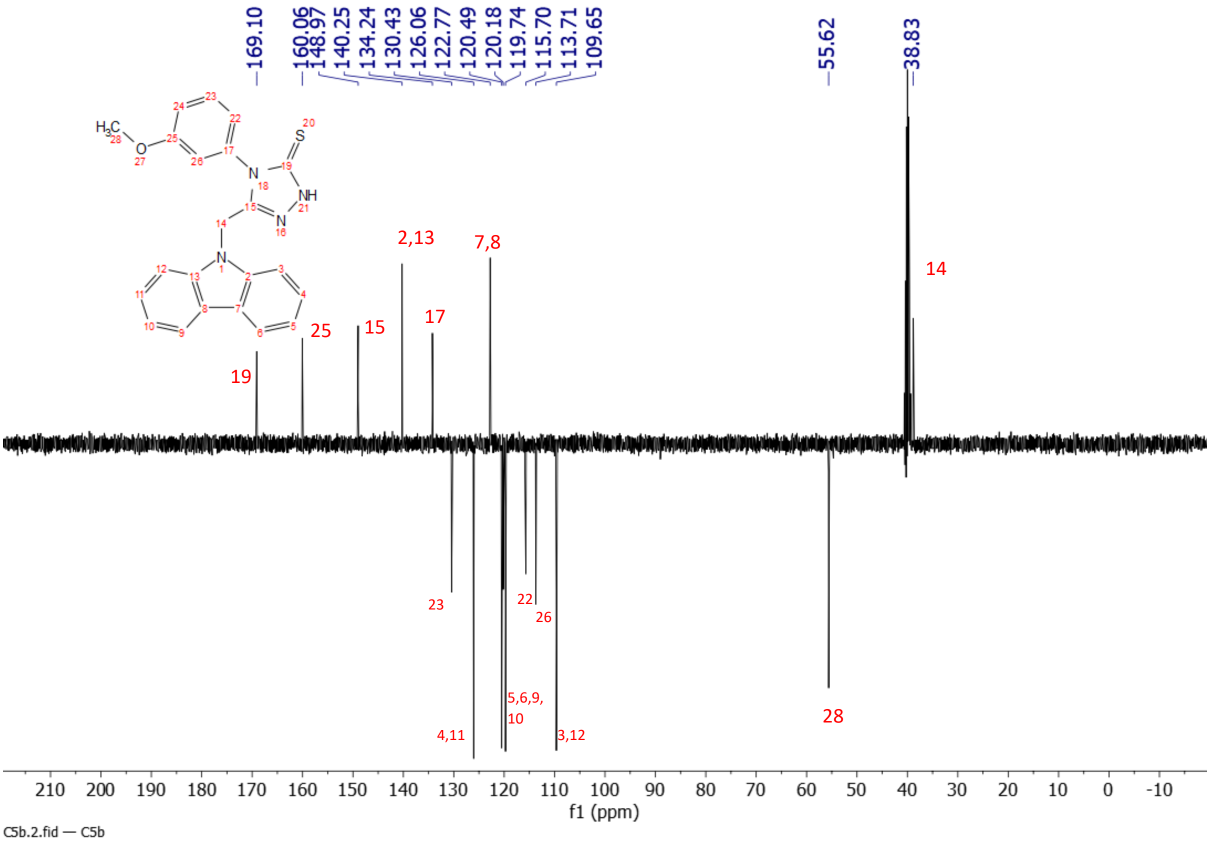

Figure S5: $^{13}C_{APT}$-NMR spectrum of Compound C5b

Spectrums of Compound C5c

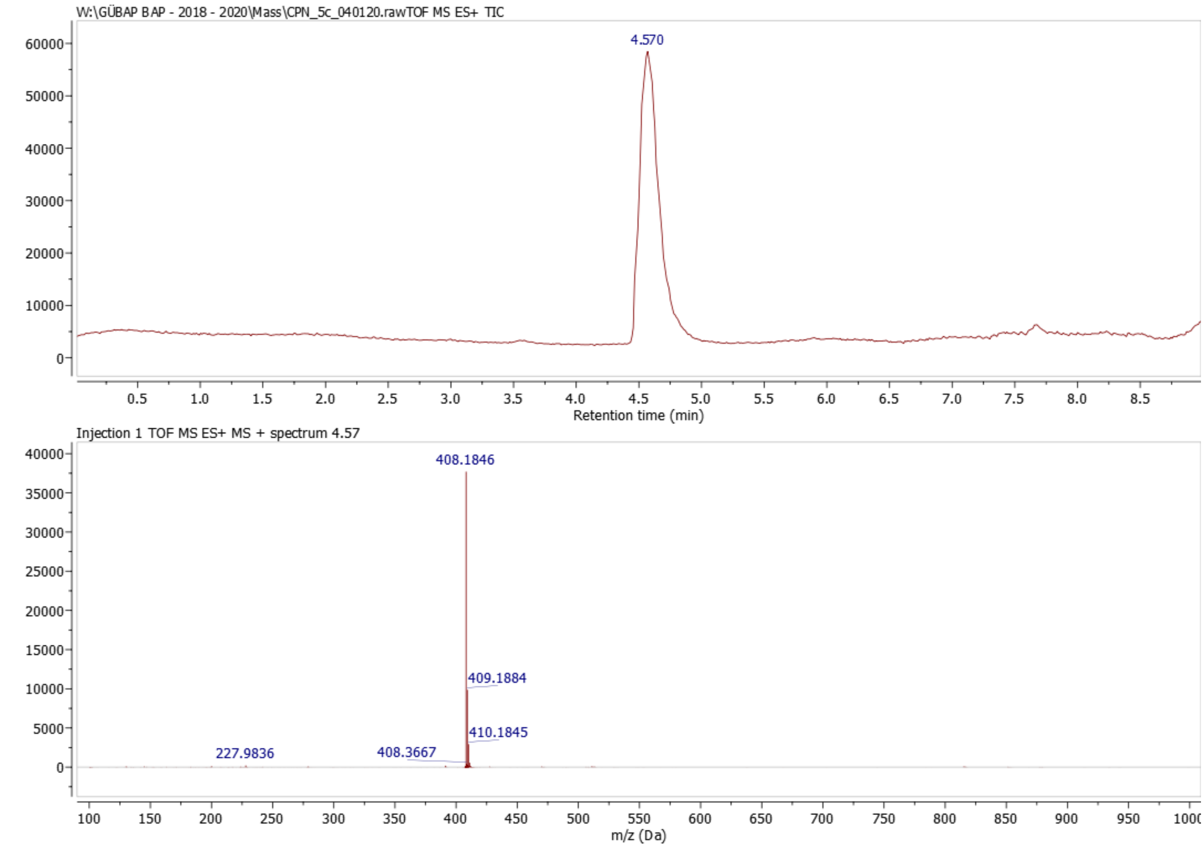

Figure S6: HRMS Spectrum of Compound C5c

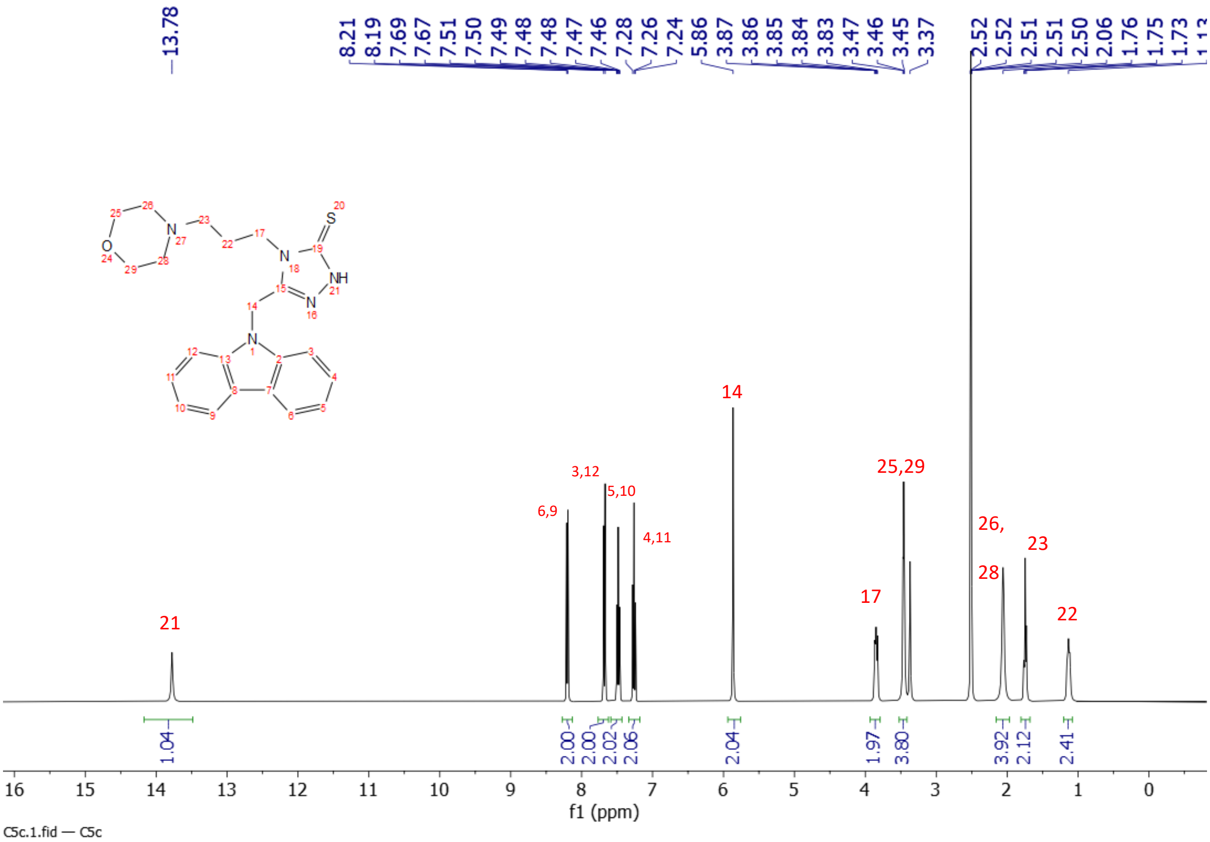

Figure S7: ^{1}H-NMR spectrum of Compound C5c

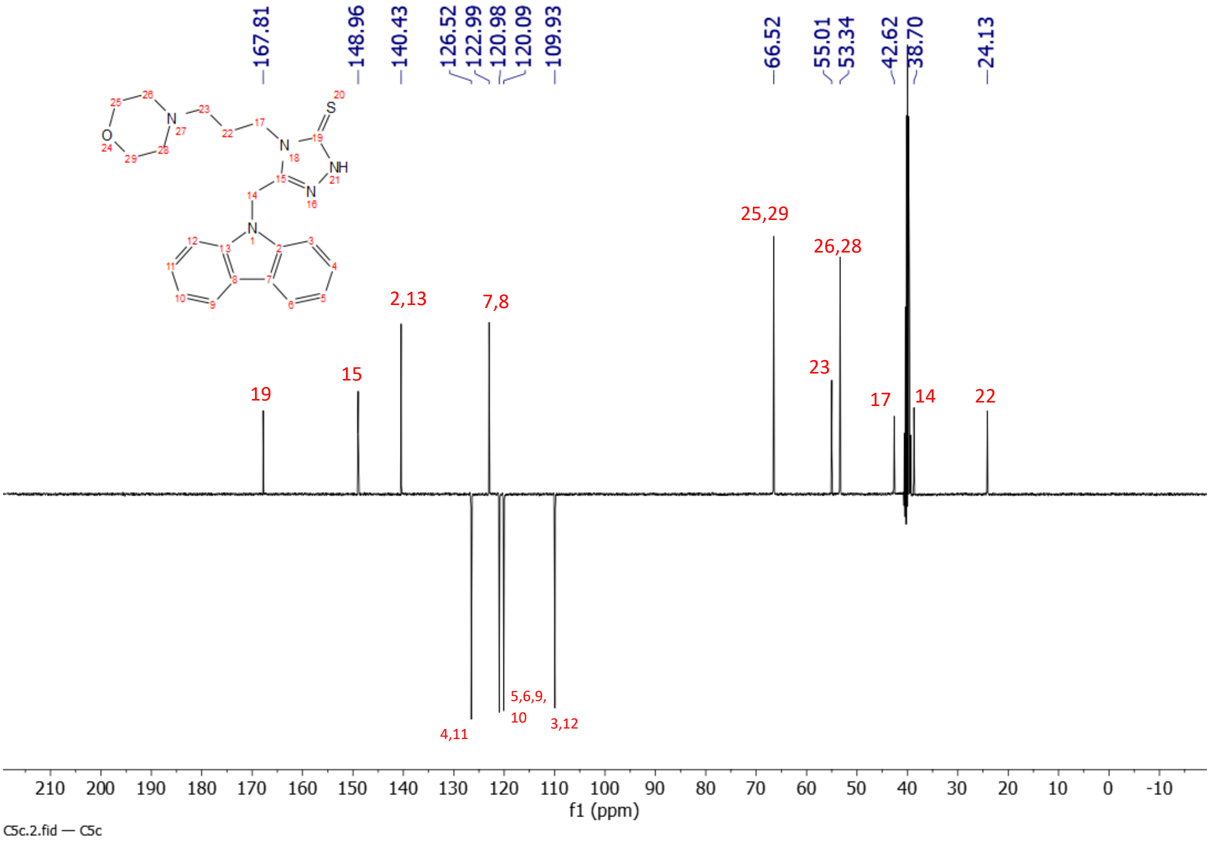

Figure S8: $^{13}C_{APT}$-NMR spectrum of Compound C5c

Spectrums of Compound C5d

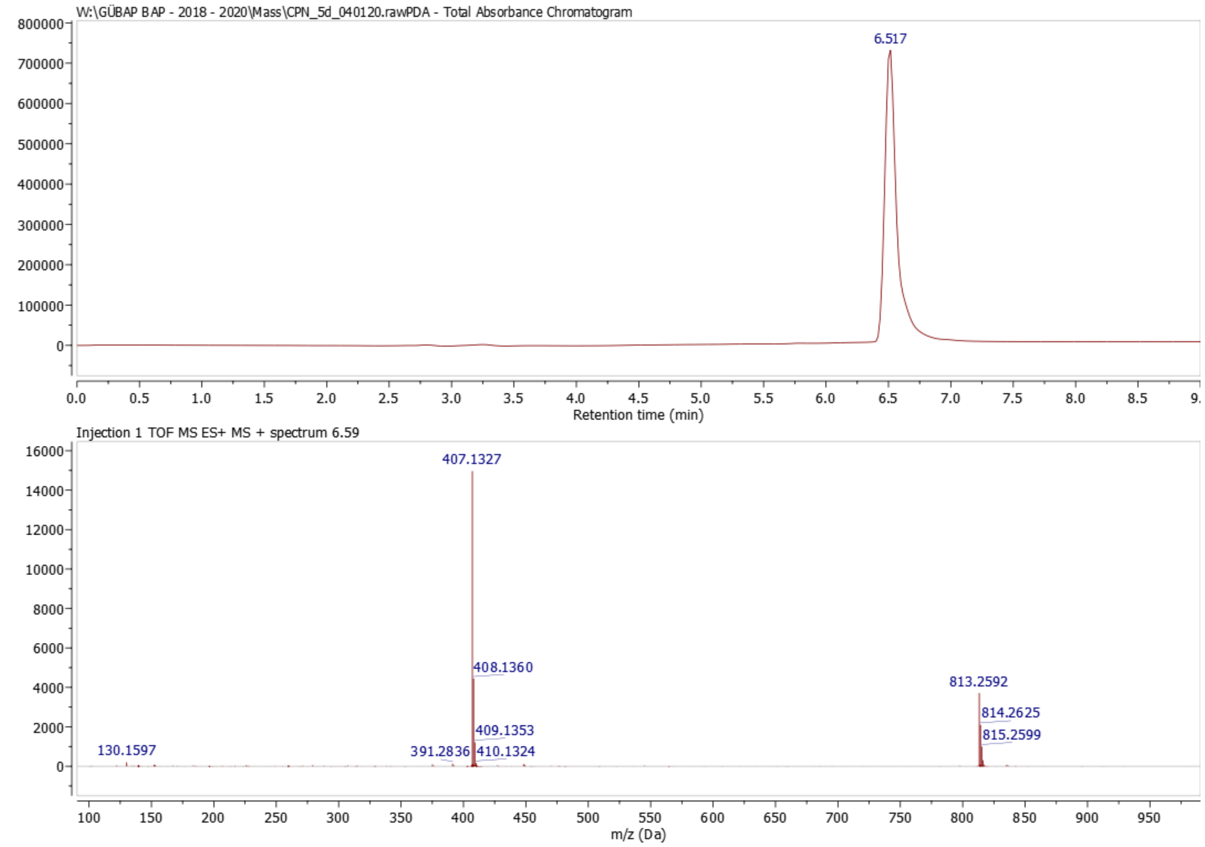

Figure S9: HRMS Spectrum of Compound C5d

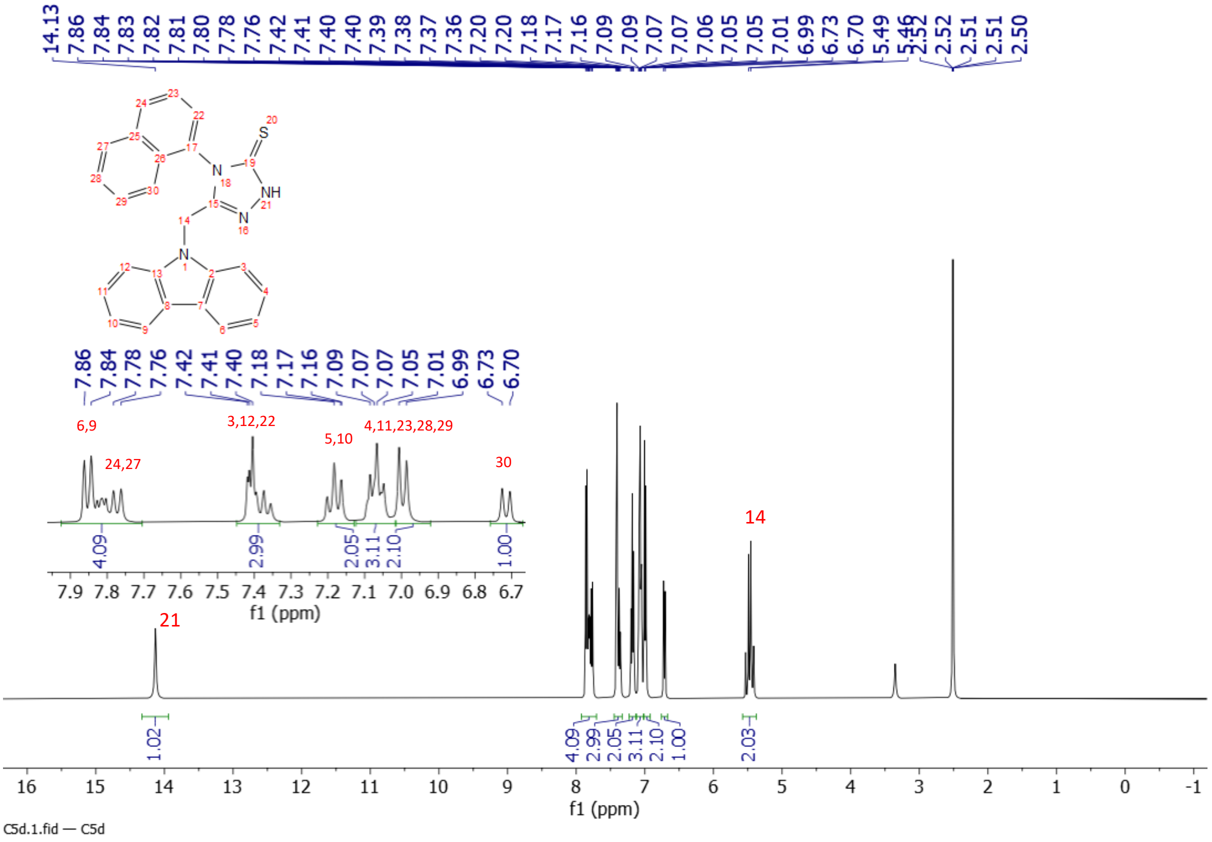

Figure S10: ^{1}H-NMR spectrum of Compound C5d

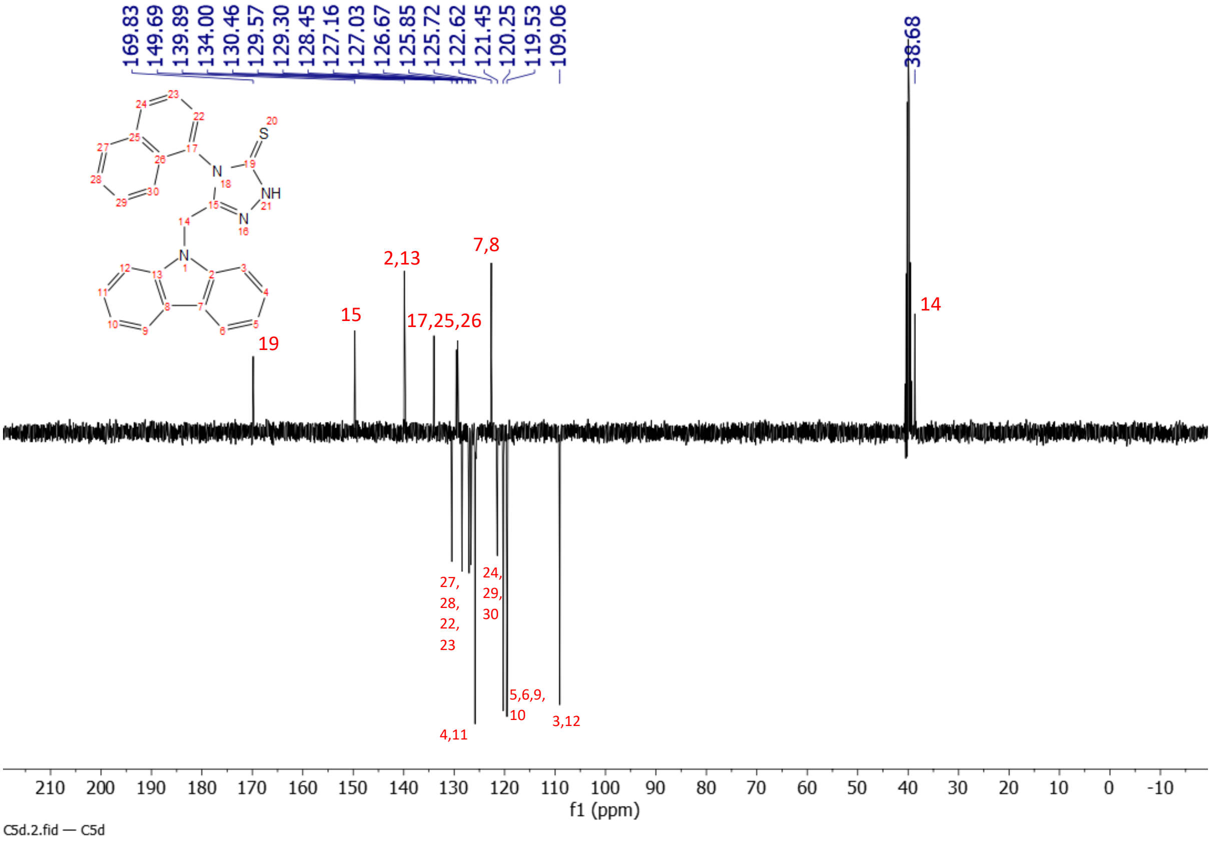

Figure S11: $^{13}C_{APT}$-NMR spectrum of Compound C5d

Spectrums of Compound C5e

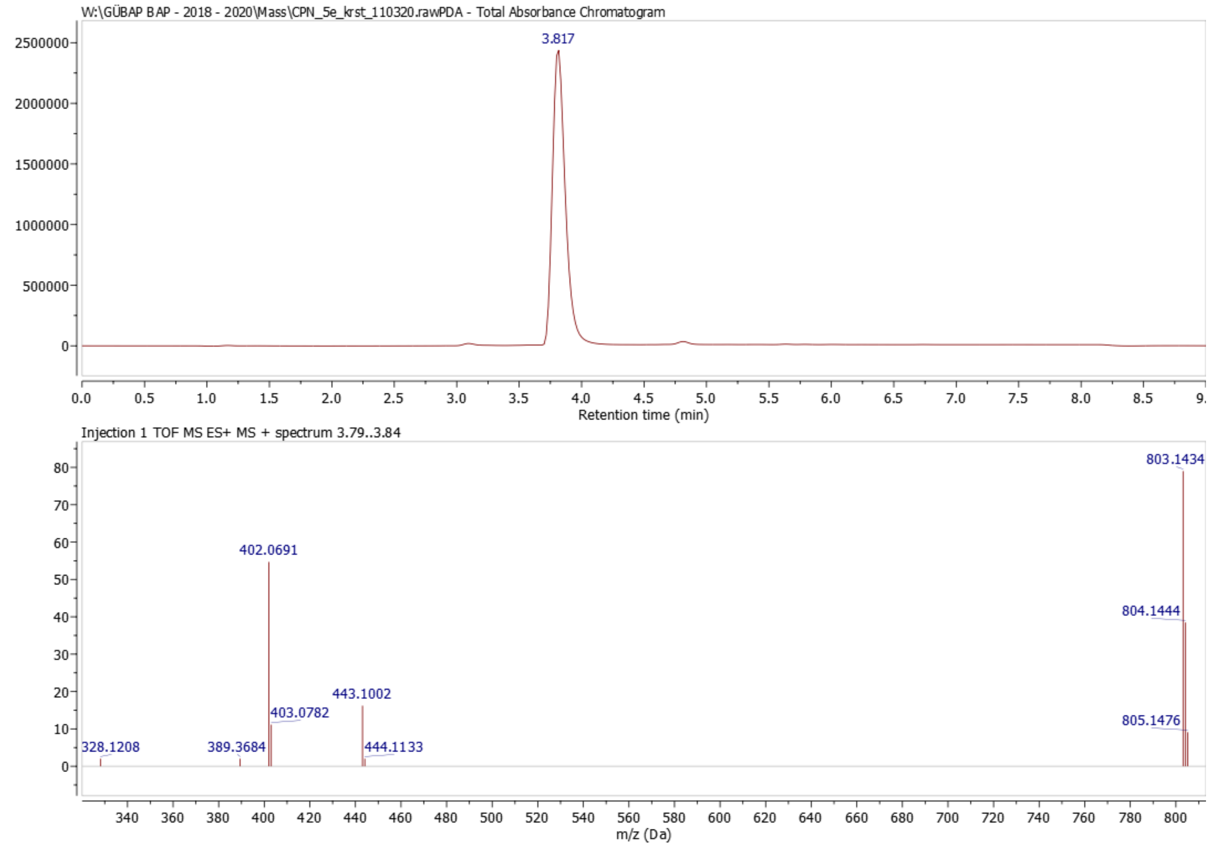

Figure S12: HRMS Spectrum of Compound C5e

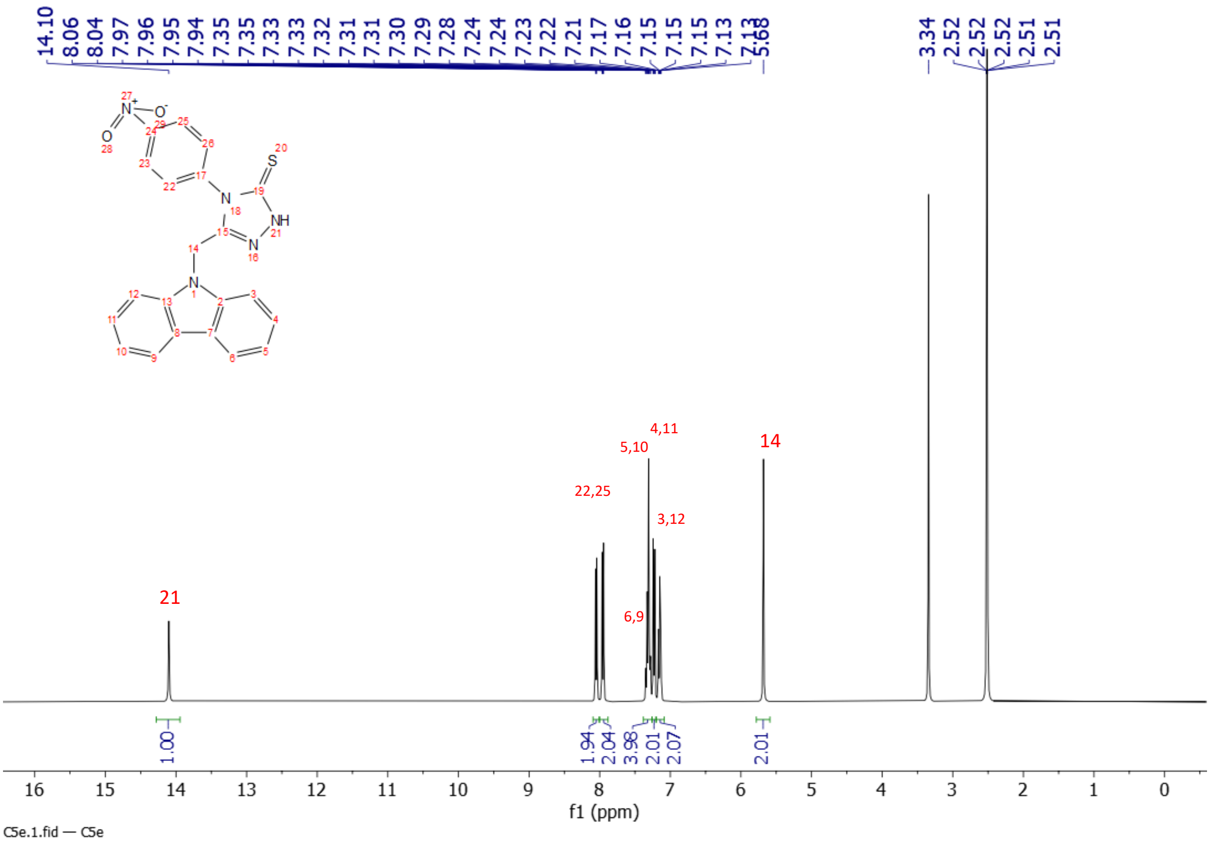

Figure S13: ^{1}H-NMR spectrum of Compound C5e

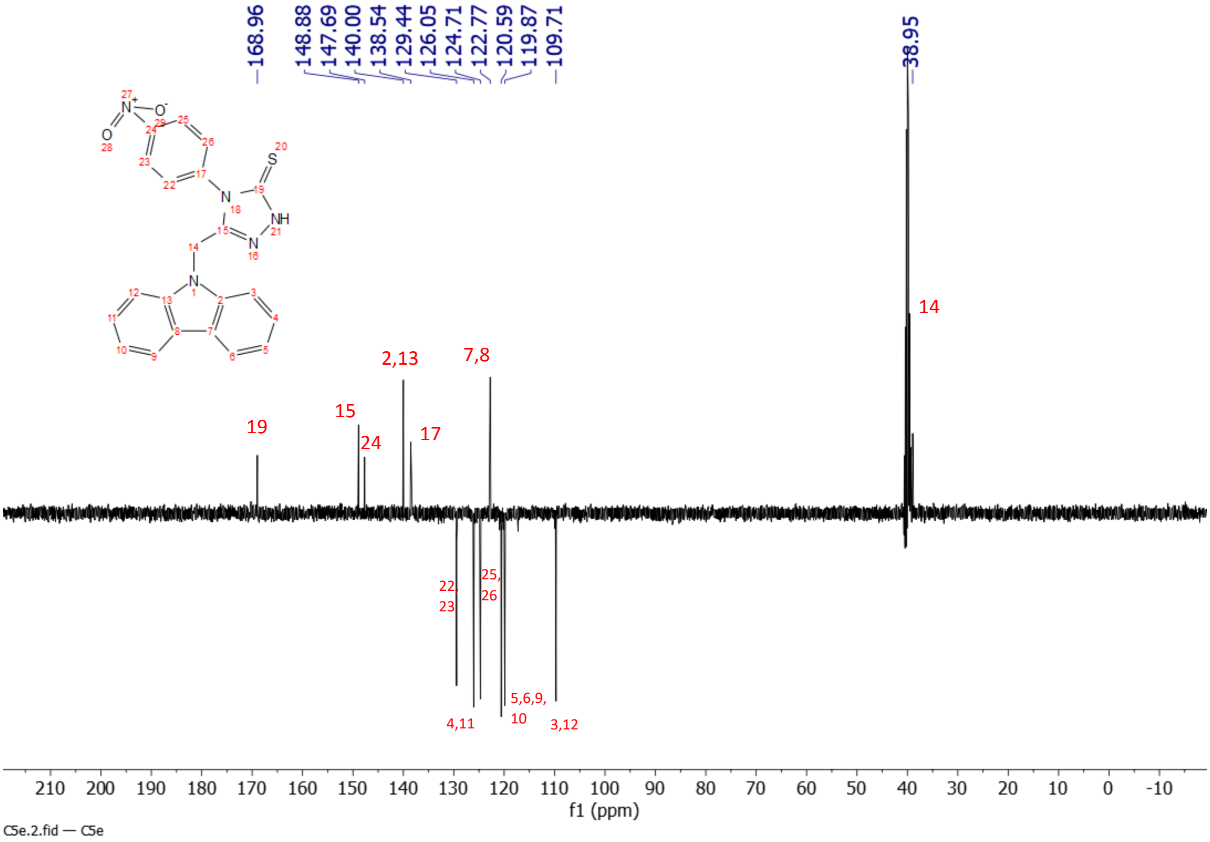

Figure S14: $^{13}C_{APT}$-NMR spectrum of Compound C5e

Spectrums of Compound C5f

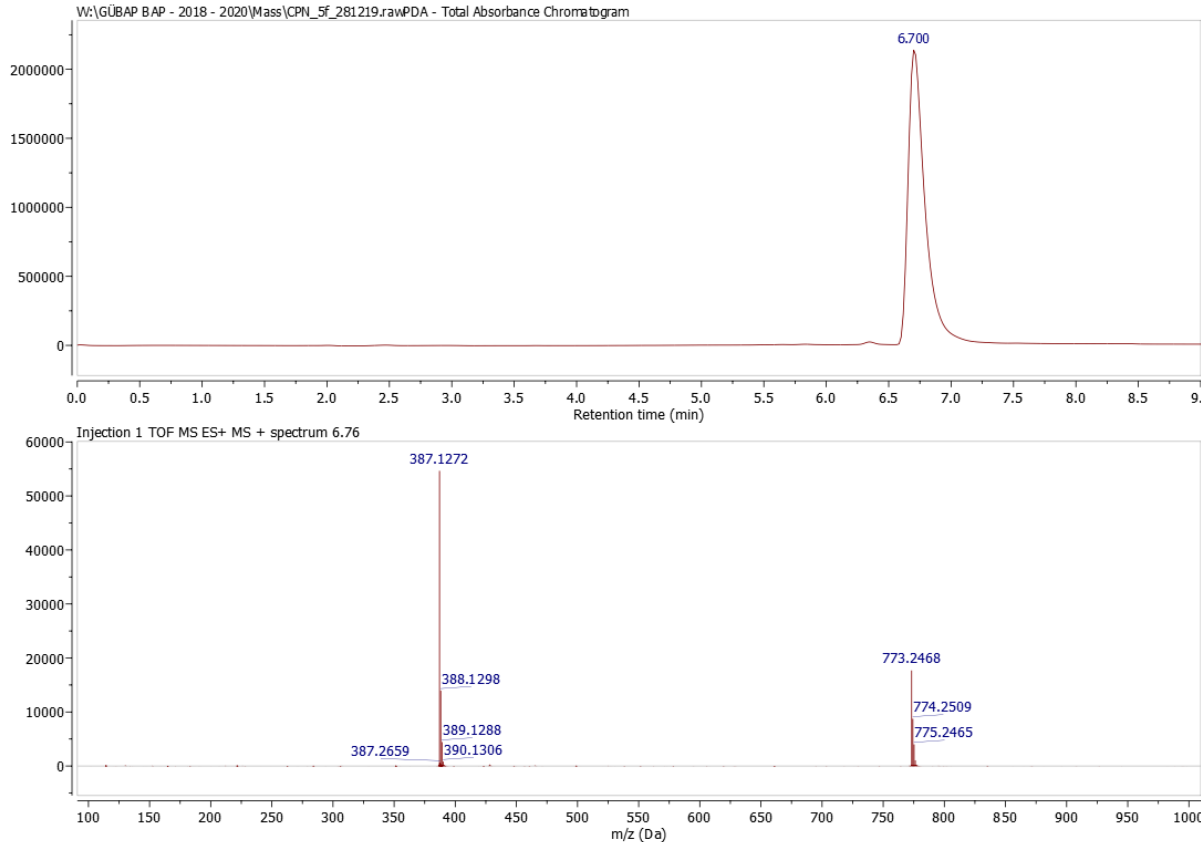

Figure S15: HRMS Spectrum of Compound C5f

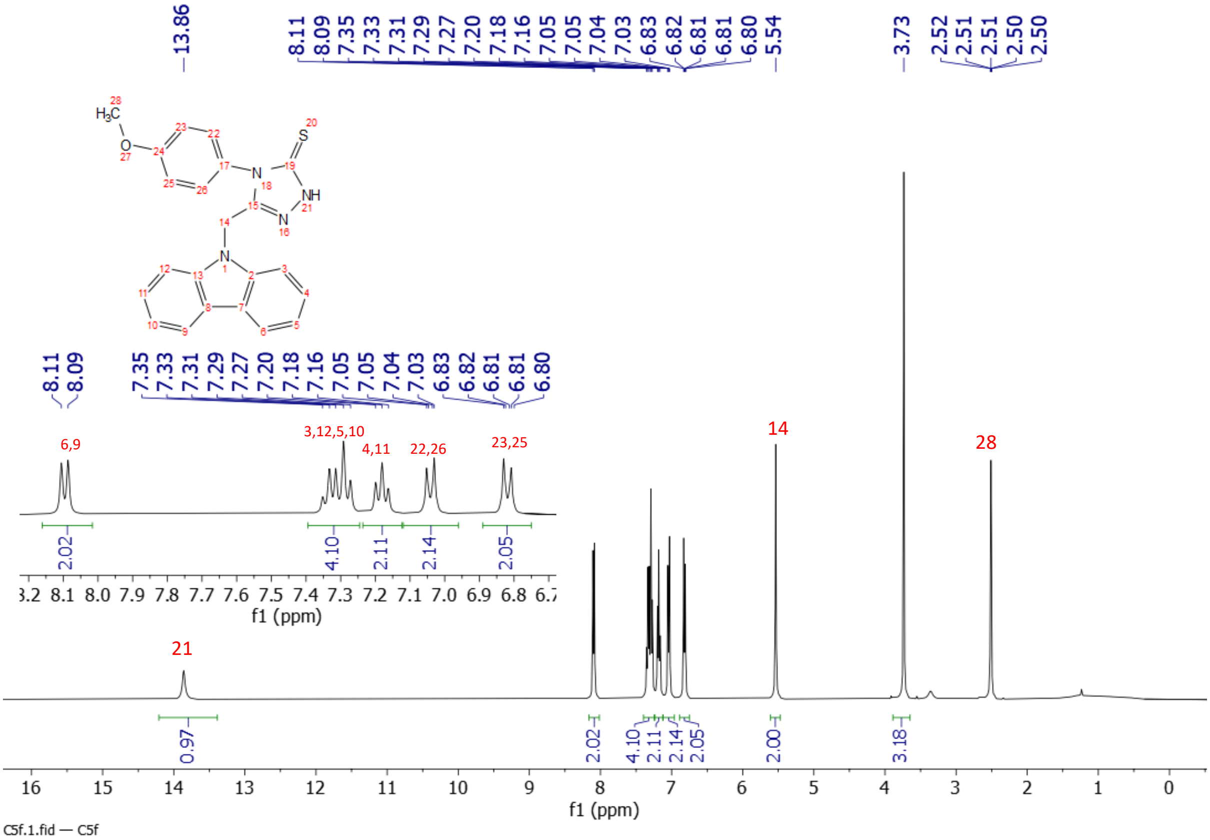

Figure S16: ¹H-NMR spectrum of Compound C5f

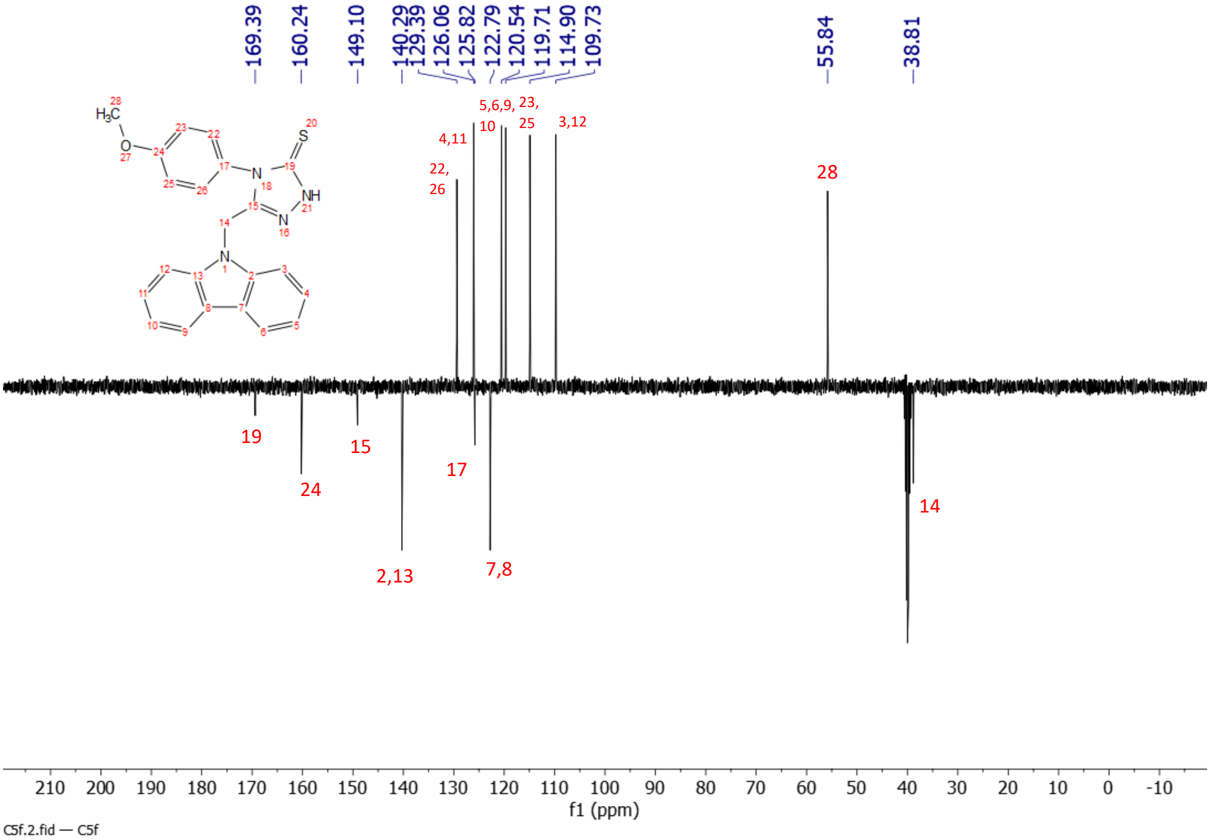

Figure S17: $^{13}C_{APT}$-NMR spectrum of Compound C5f

Spectrums of Compound C5g

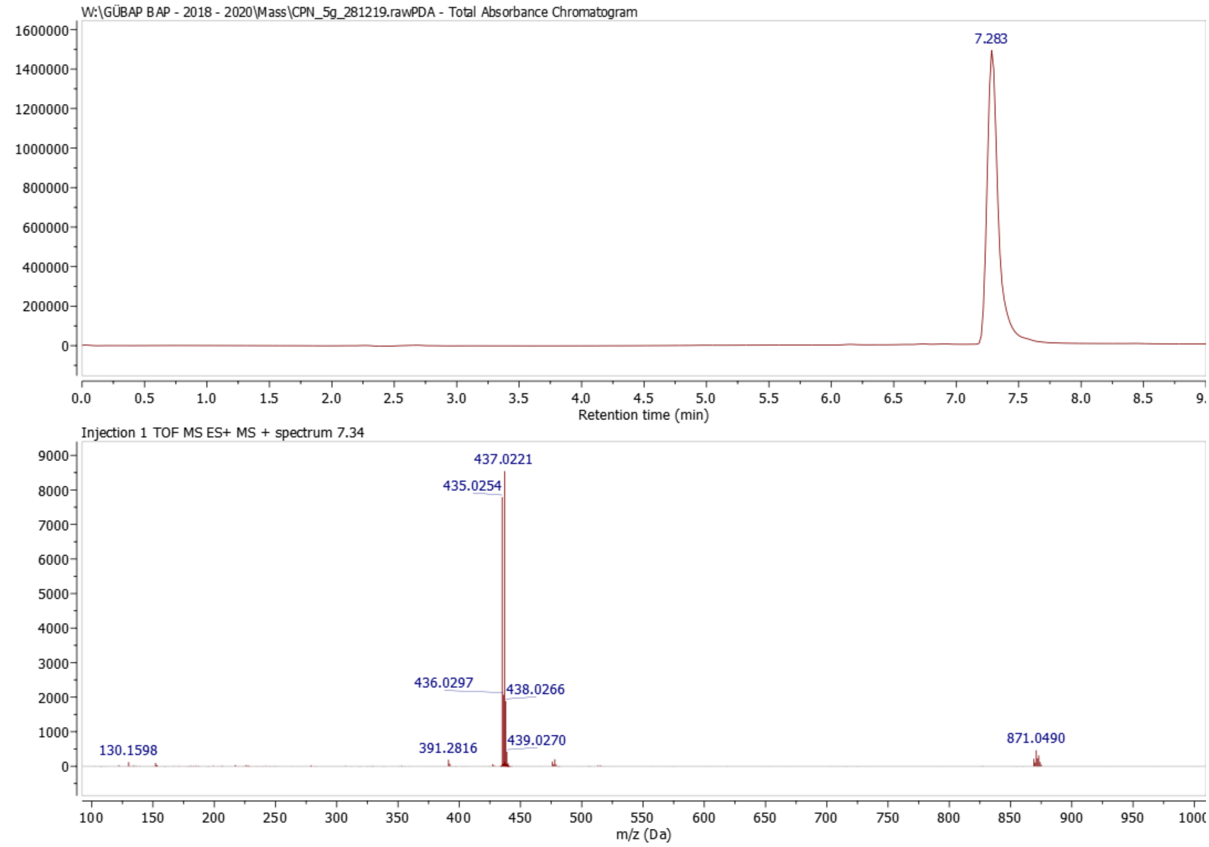

Figure S18: HRMS Spectrum of Compound C5g

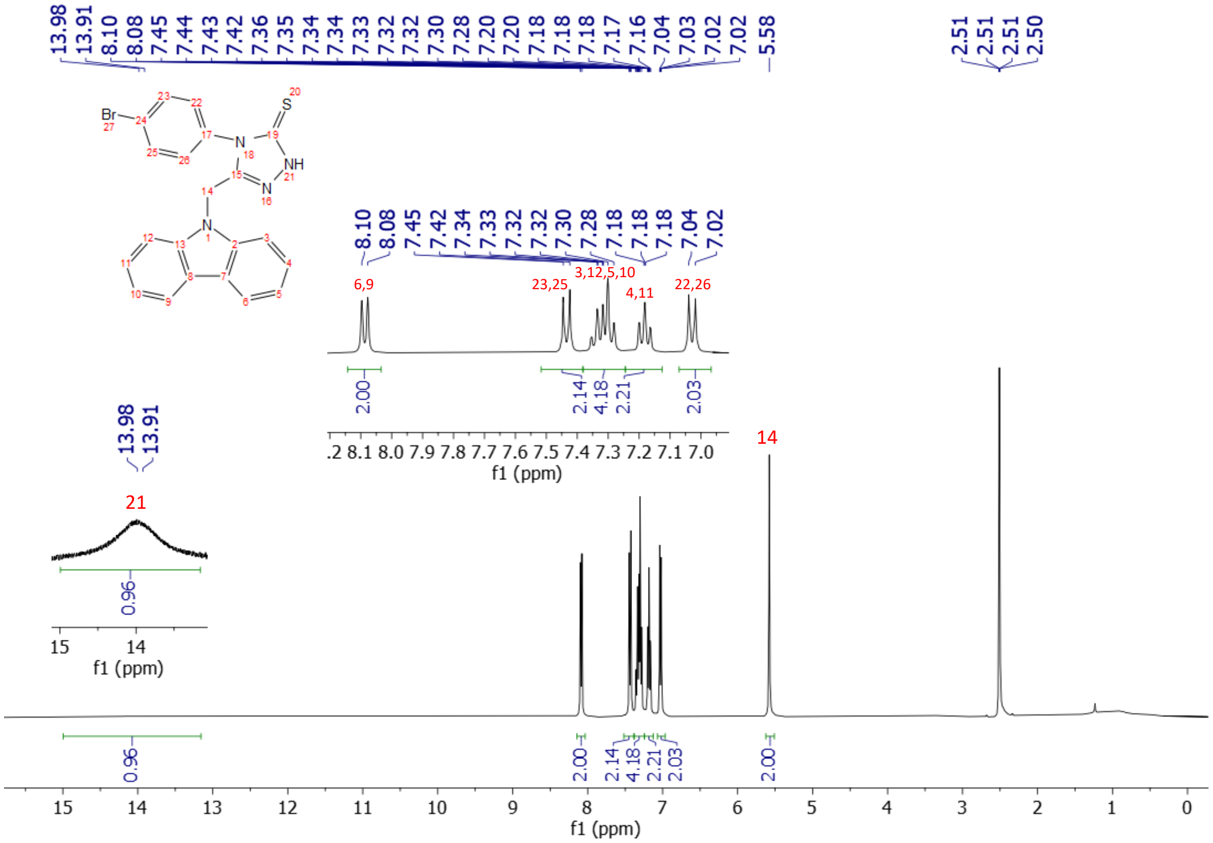

Figure S19: ¹H-NMR spectrum of Compound C5g

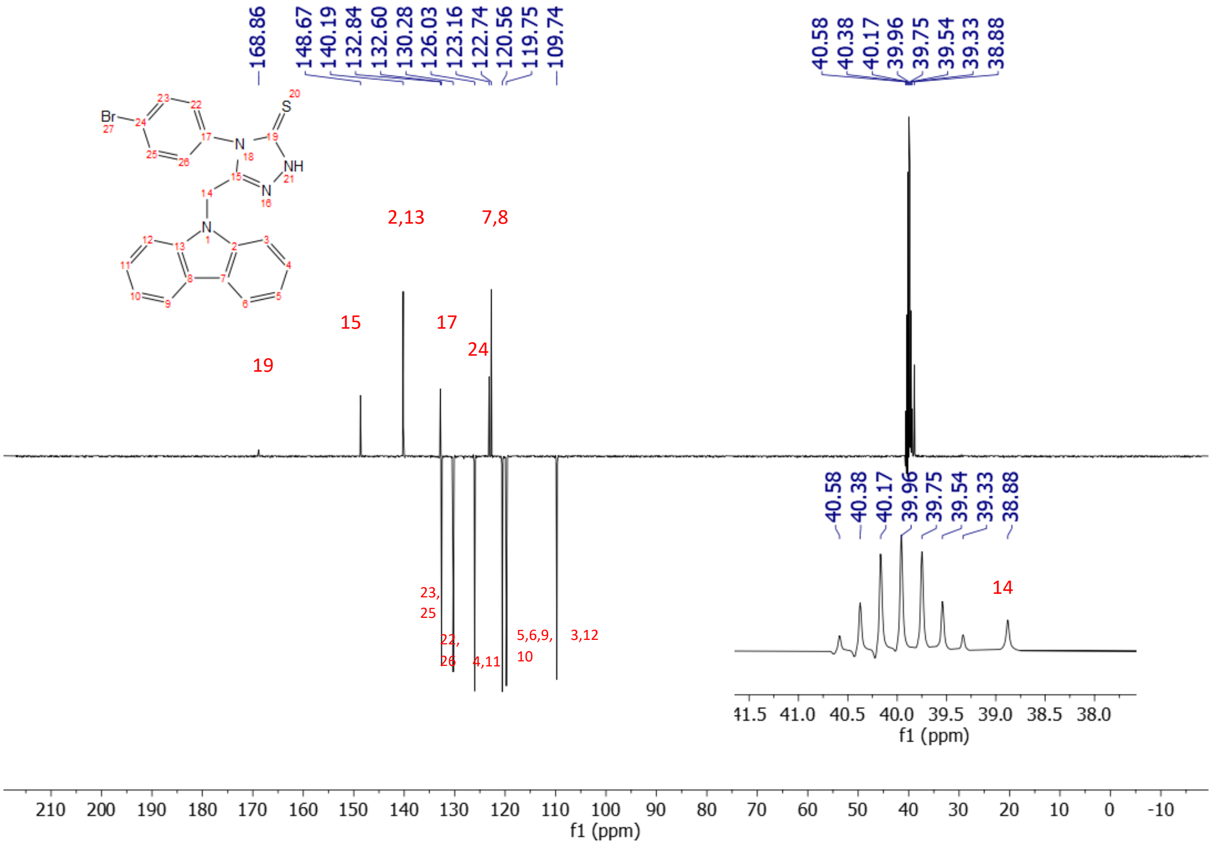

Figure S20: $^{13}C_{APT}$-NMR spectrum of Compound C5g

Spectrums of Compound C5h

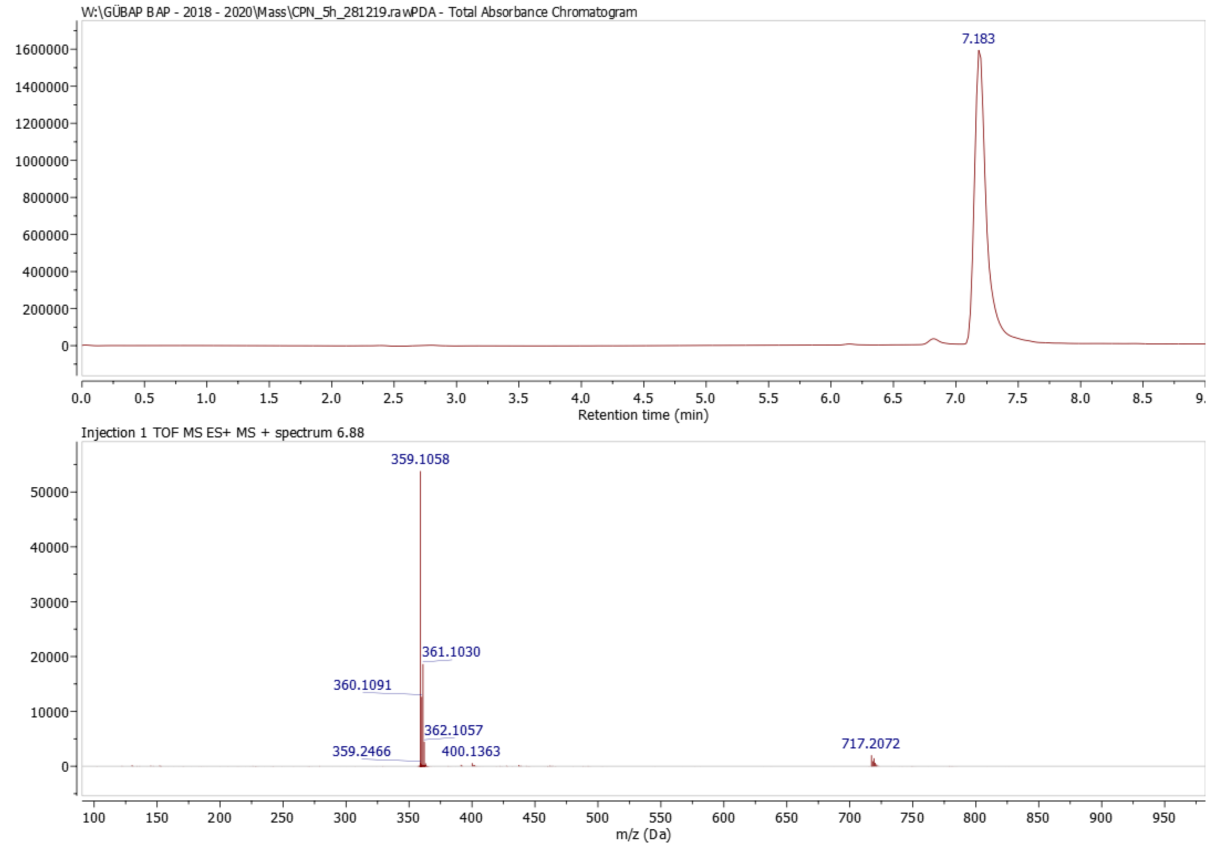

Figure S21: HRMS Spectrum of Compound C5h

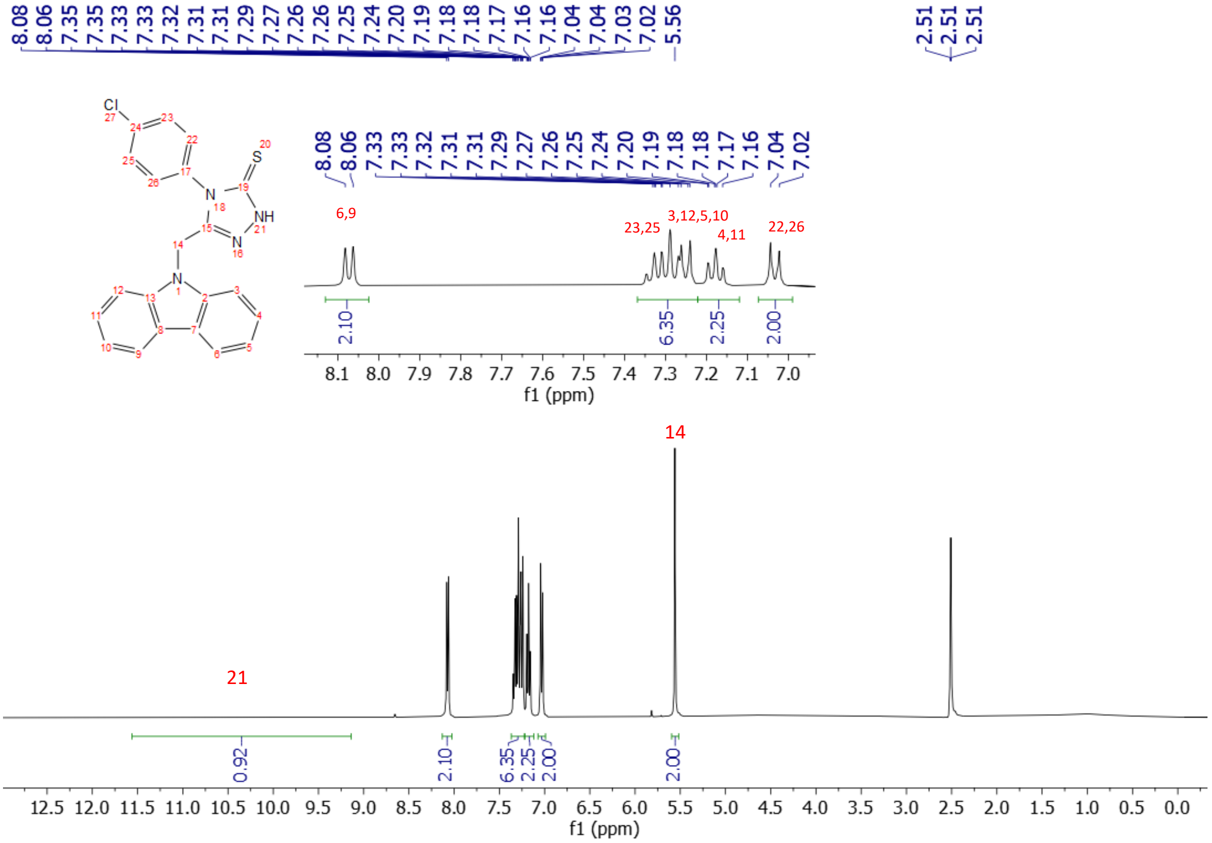

Figure S22: ^{1}H-NMR spectrum of Compound C5h

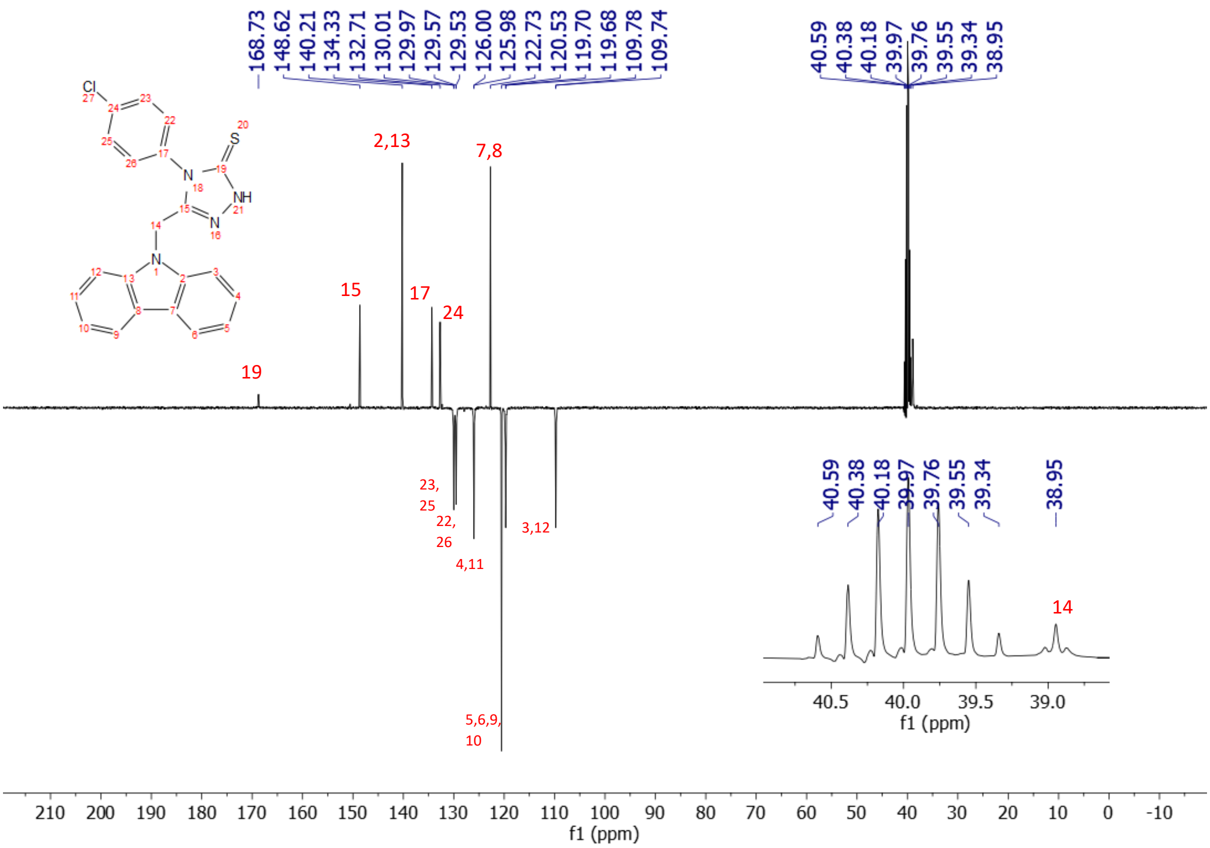

Figure S23: $^{13}C_{APT}$-NMR spectrum of Compound C5h

Spectrums of Compound C5k

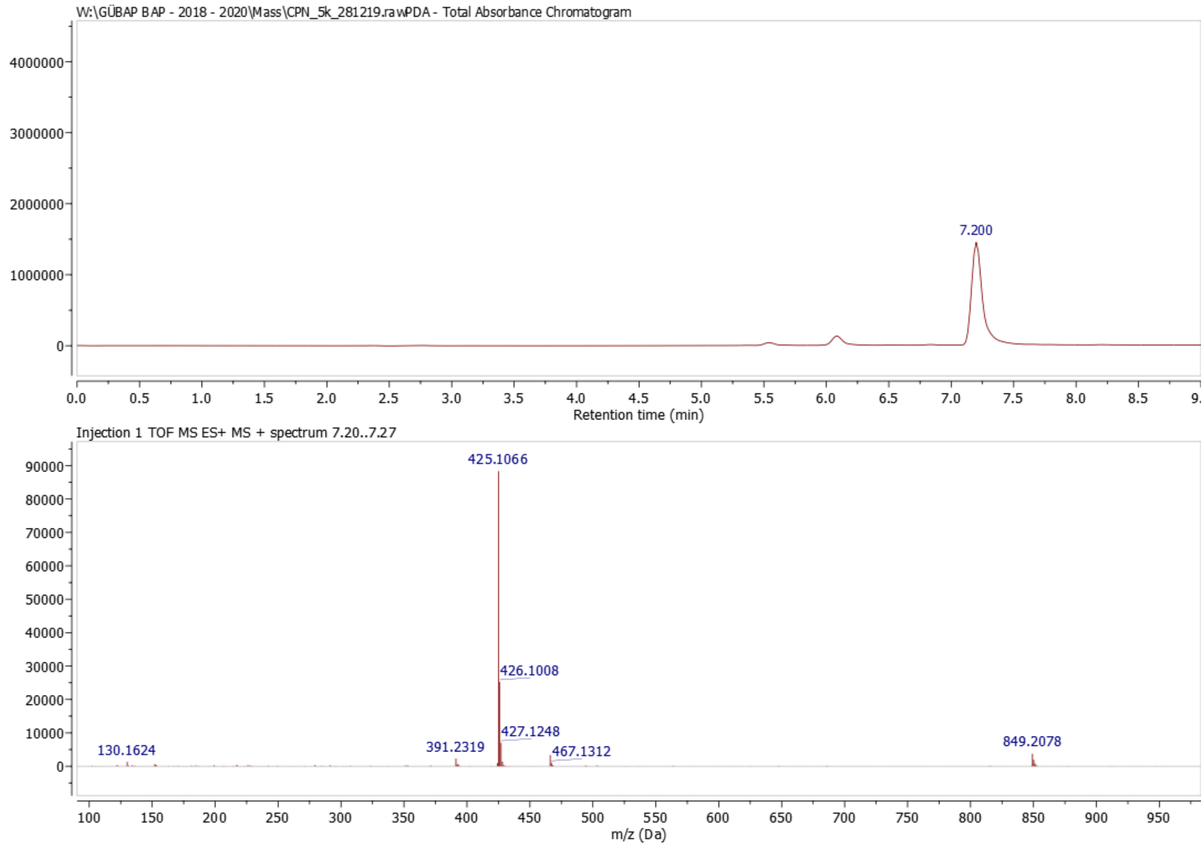

Figure S24: HRMS Spectrum of Compound C5k

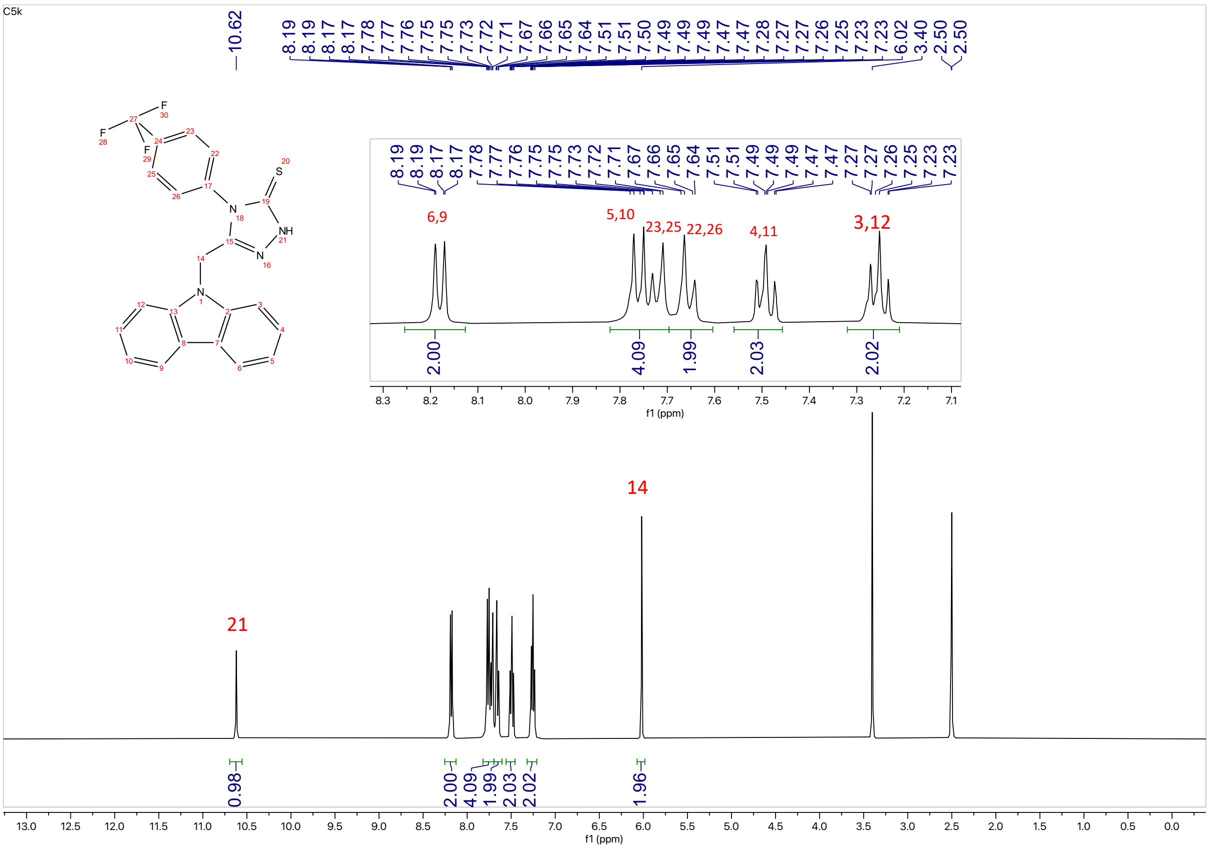

Figure S25: ^{1}H-NMR spectrum of Compound C5k

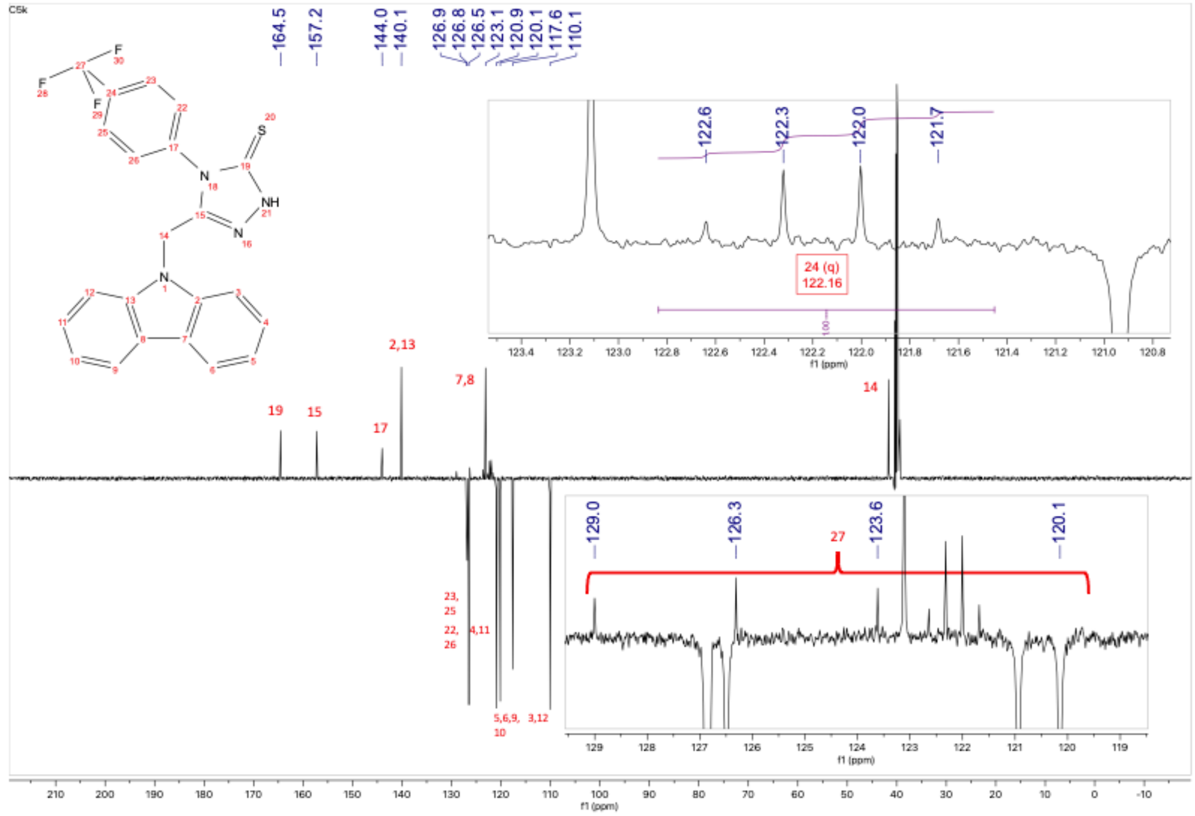

Figure S26: $^{13}C_{APT}$-NMR spectrum of Compound C5k

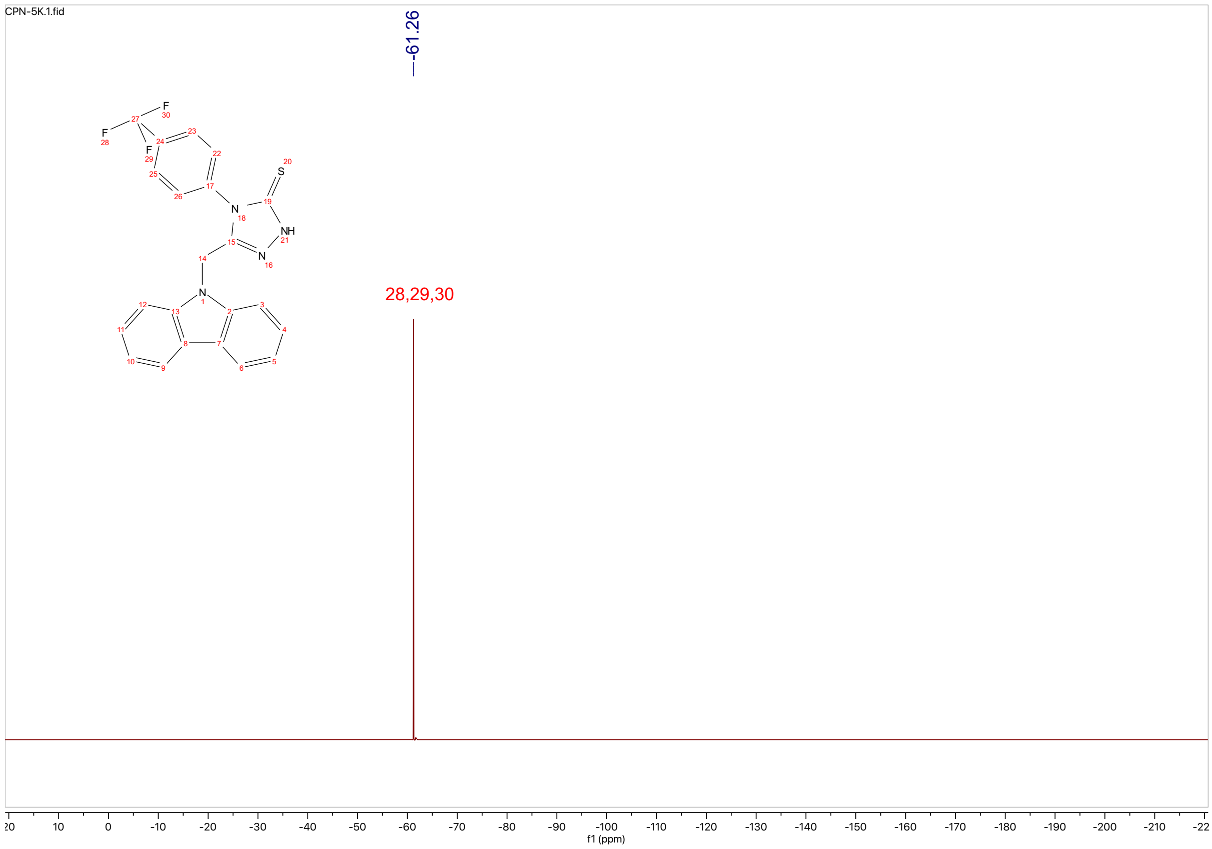

Figure S27: ^{19}F-NMR spectrum of Compound C5k

Spectrums of Compound C5m

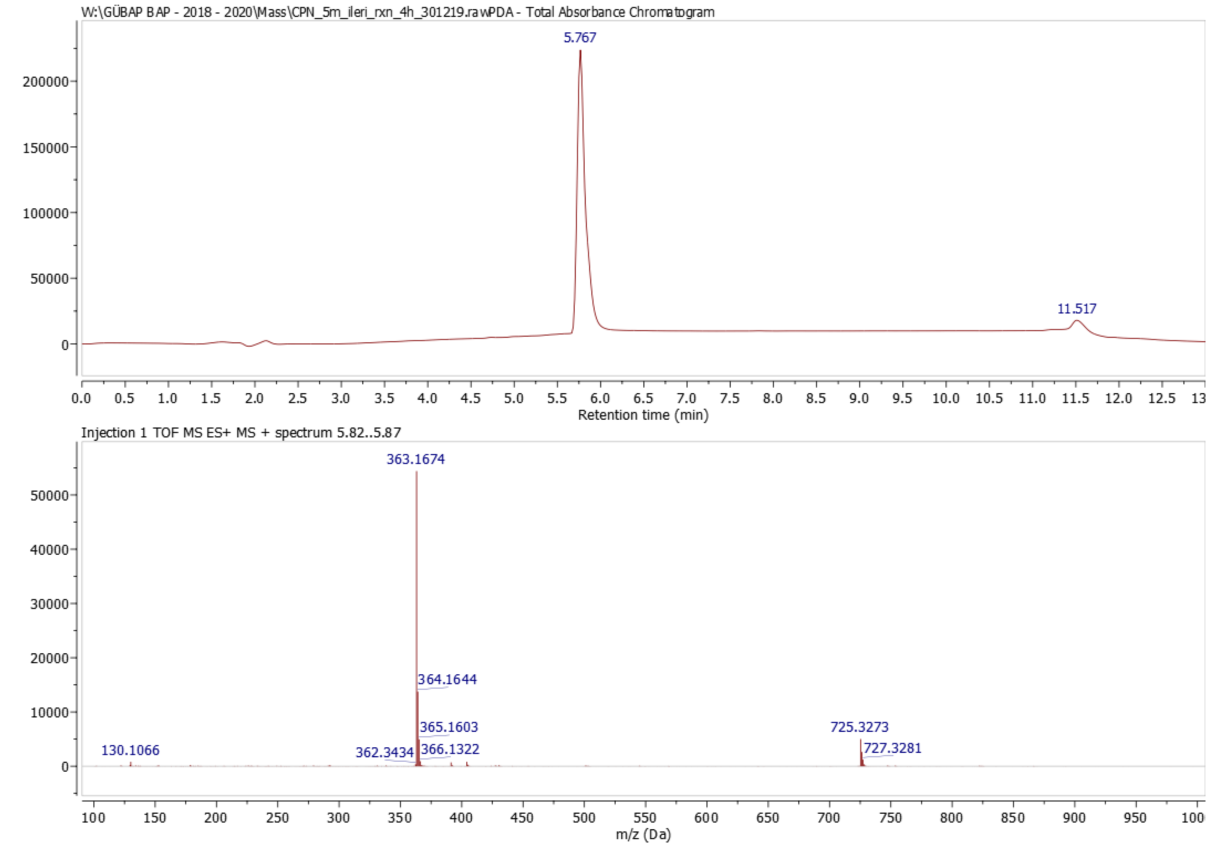

Figure S28: HRMS Spectrum of Compound C5m

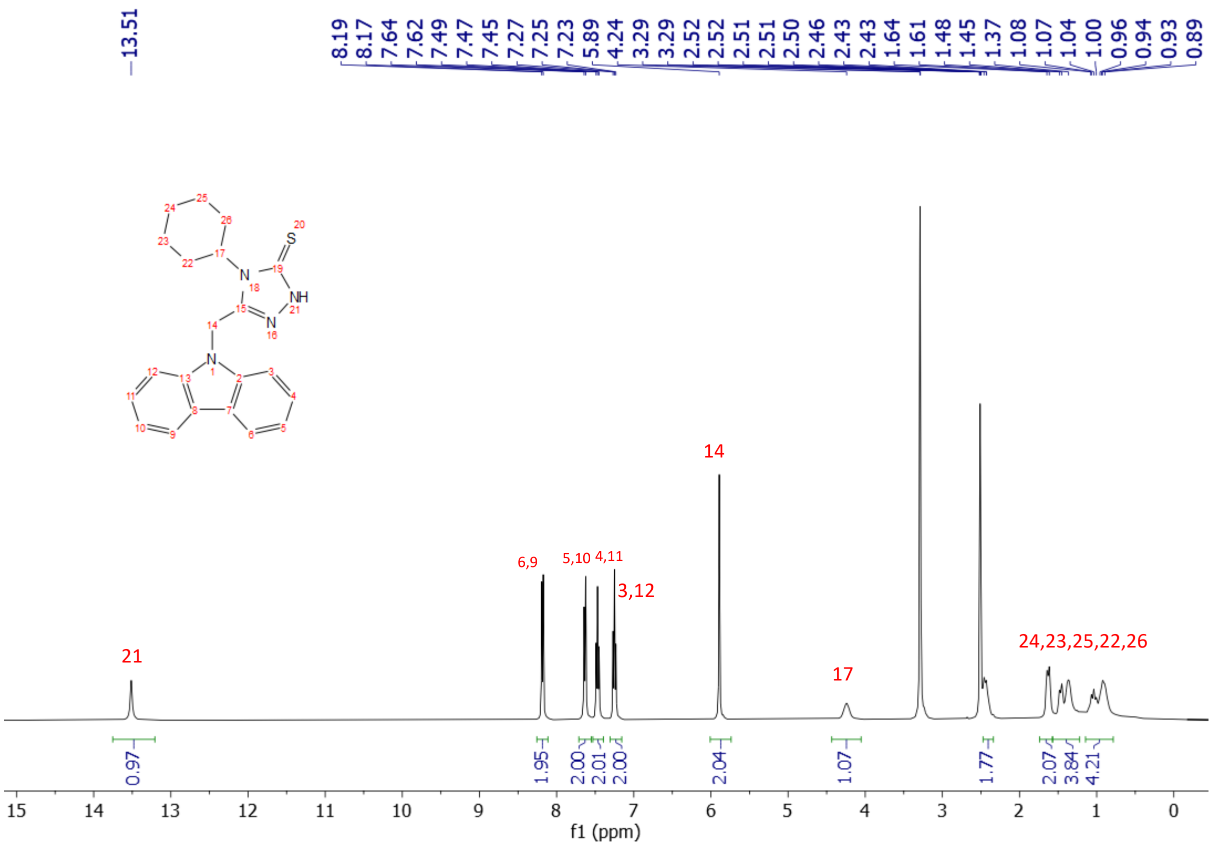

Figure S29: ¹H-NMR spectrum of Compound C5m

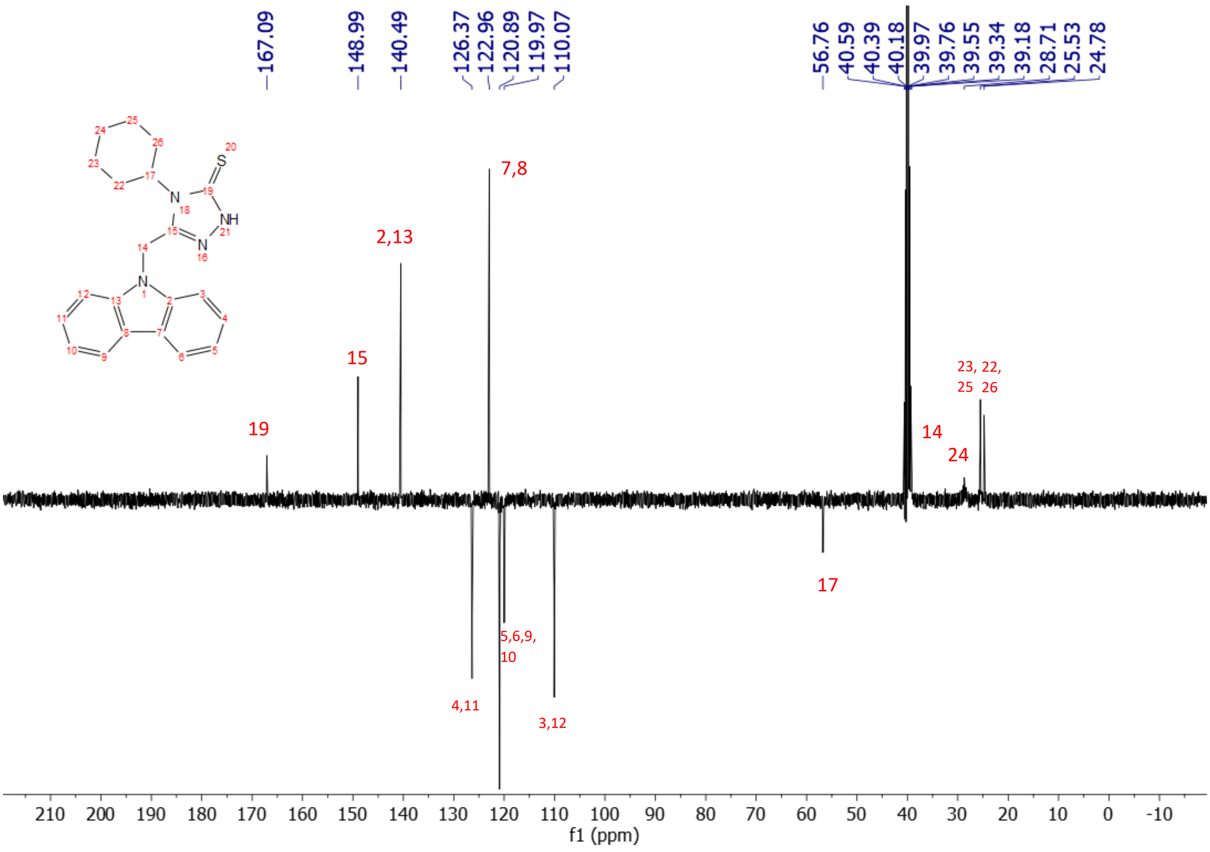

Figure S30: $^{13}C_{APT}$-NMR spectrum of Compound C5m

Spectrums of Compound C5n

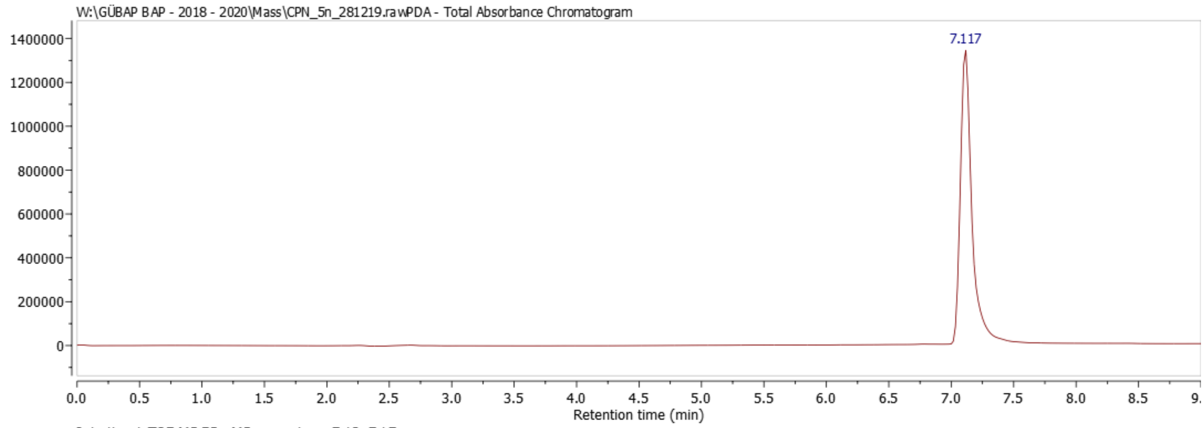

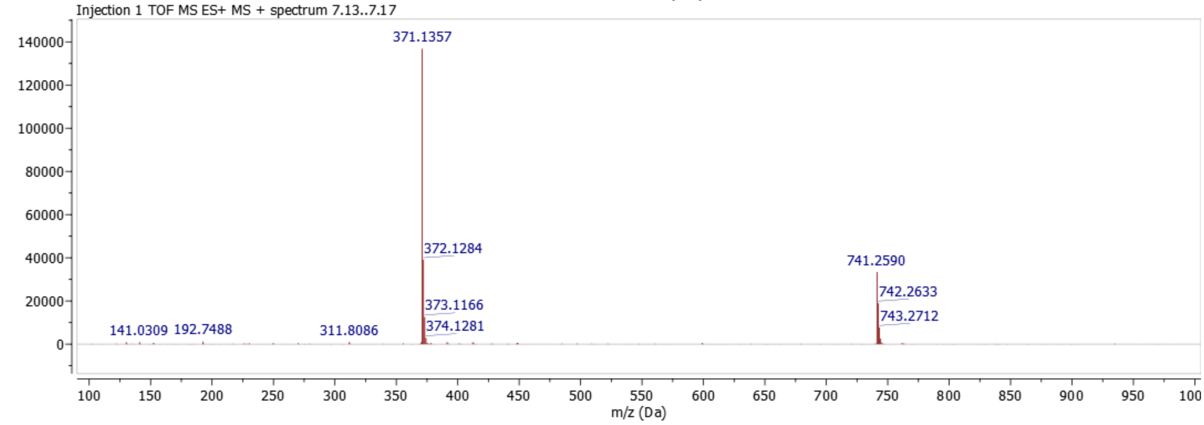

Figure S31: HRMS Spectrum of Compound C5n

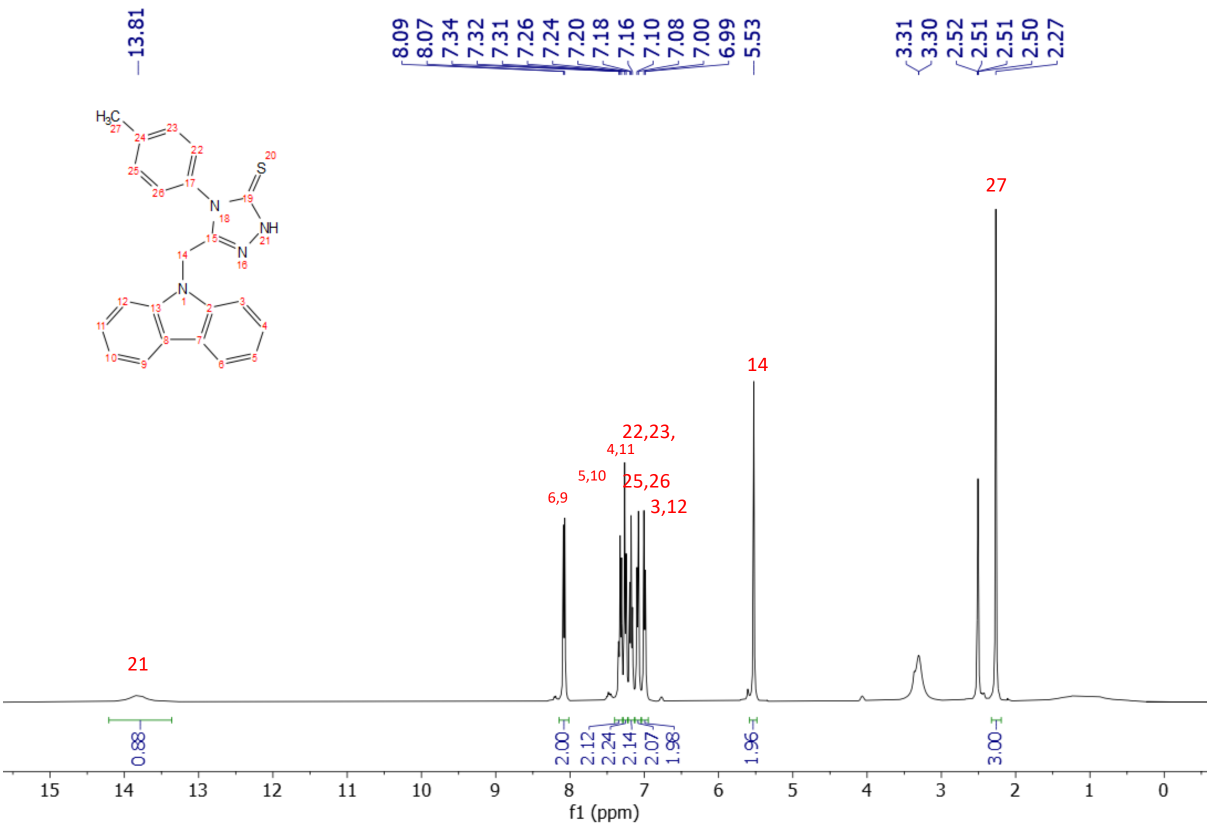

Figure S32: ¹H-NMR spectrum of Compound C5n

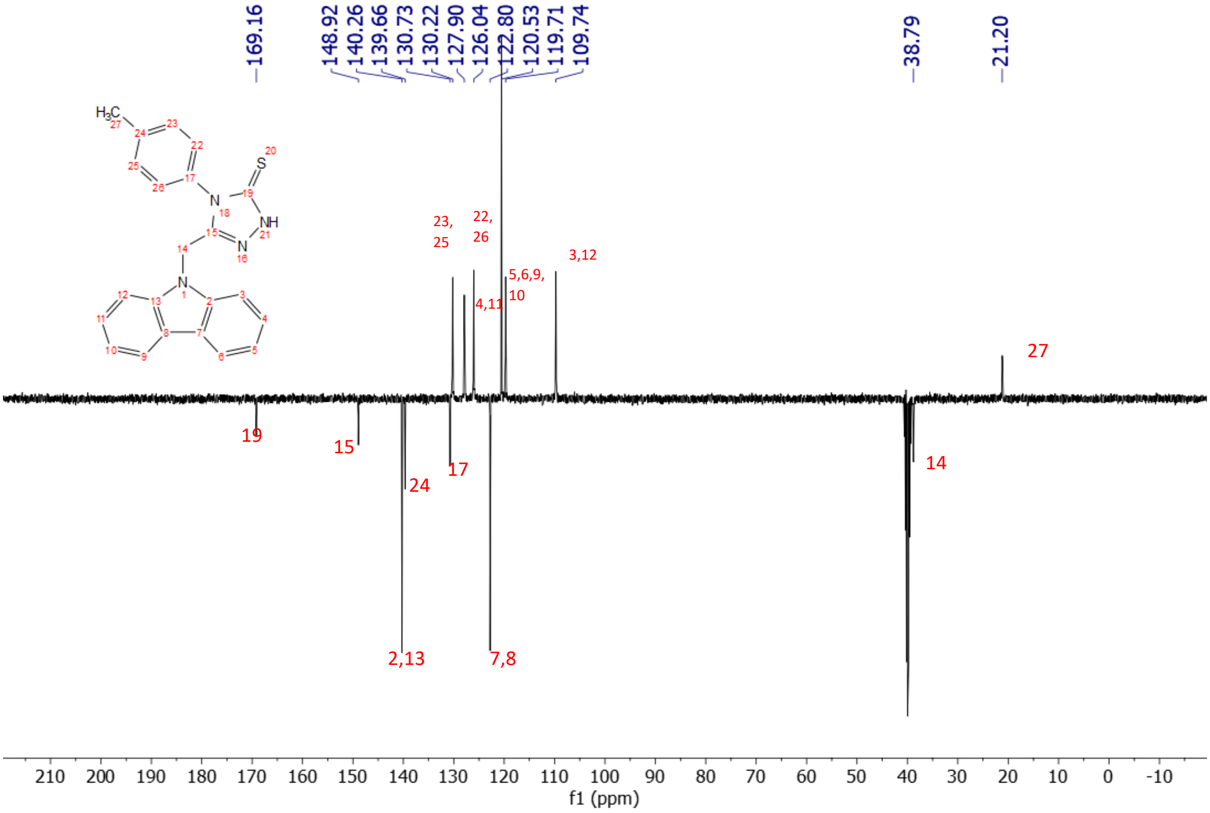

Figure S33: $^{13}C_{APT}$-NMR spectrum of Compound C5n

Spectrums of Compound C5o

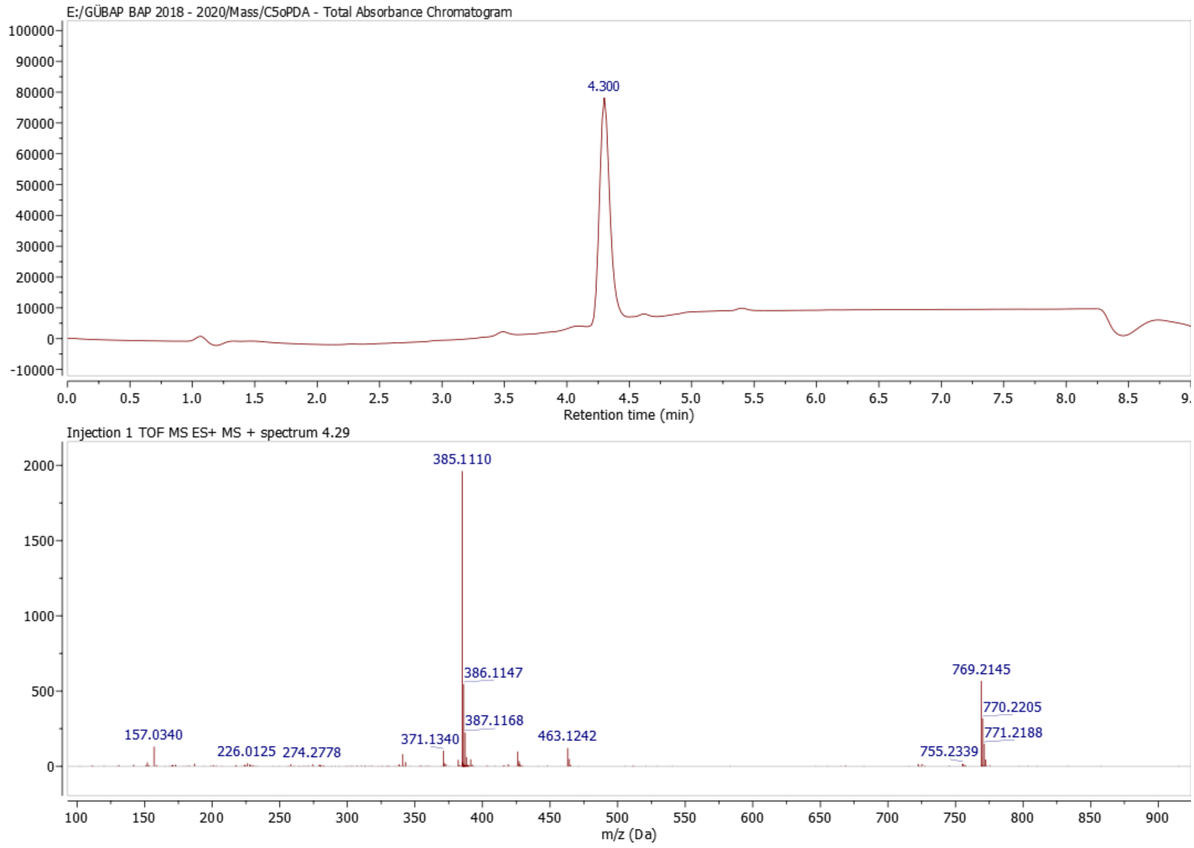

Figure S34: HRMS Spectrum of Compound C5o

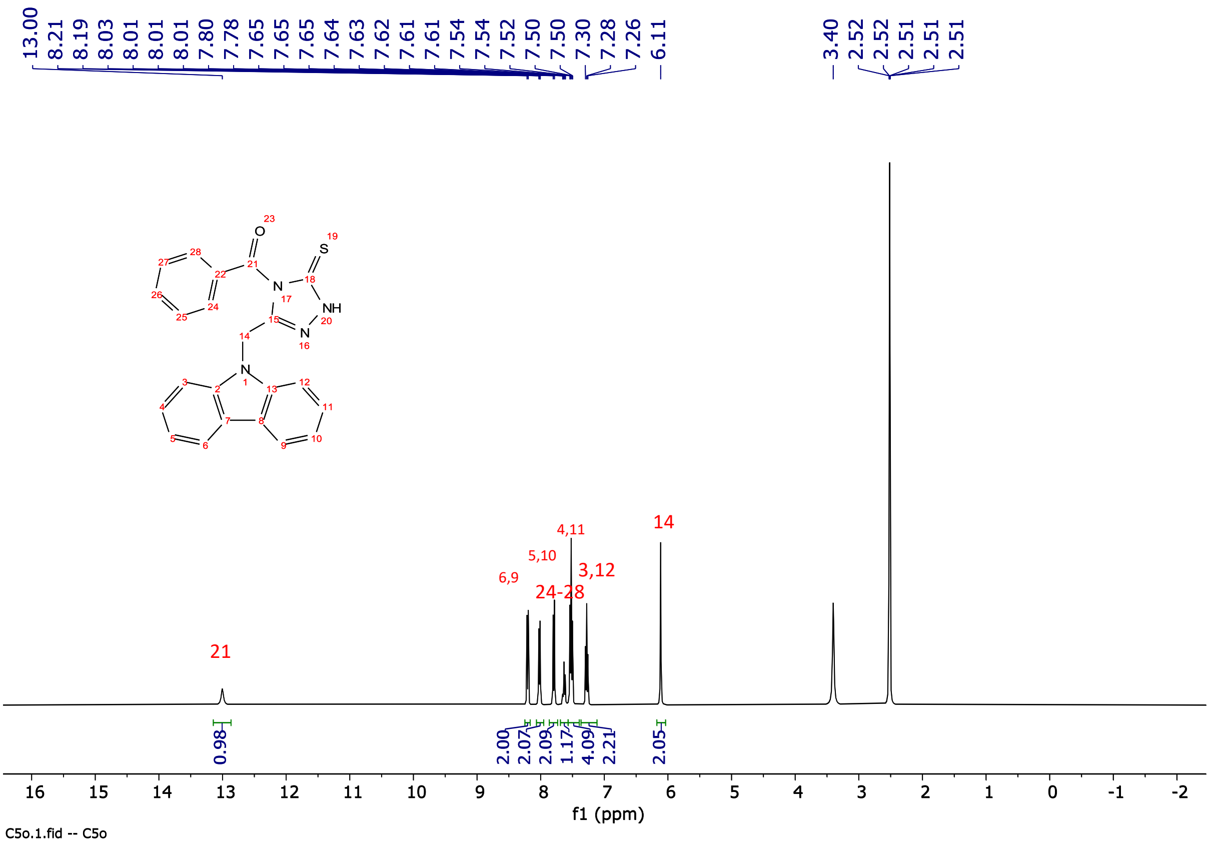

Figure S35: ^{1}H-NMR spectrum of Compound C5o

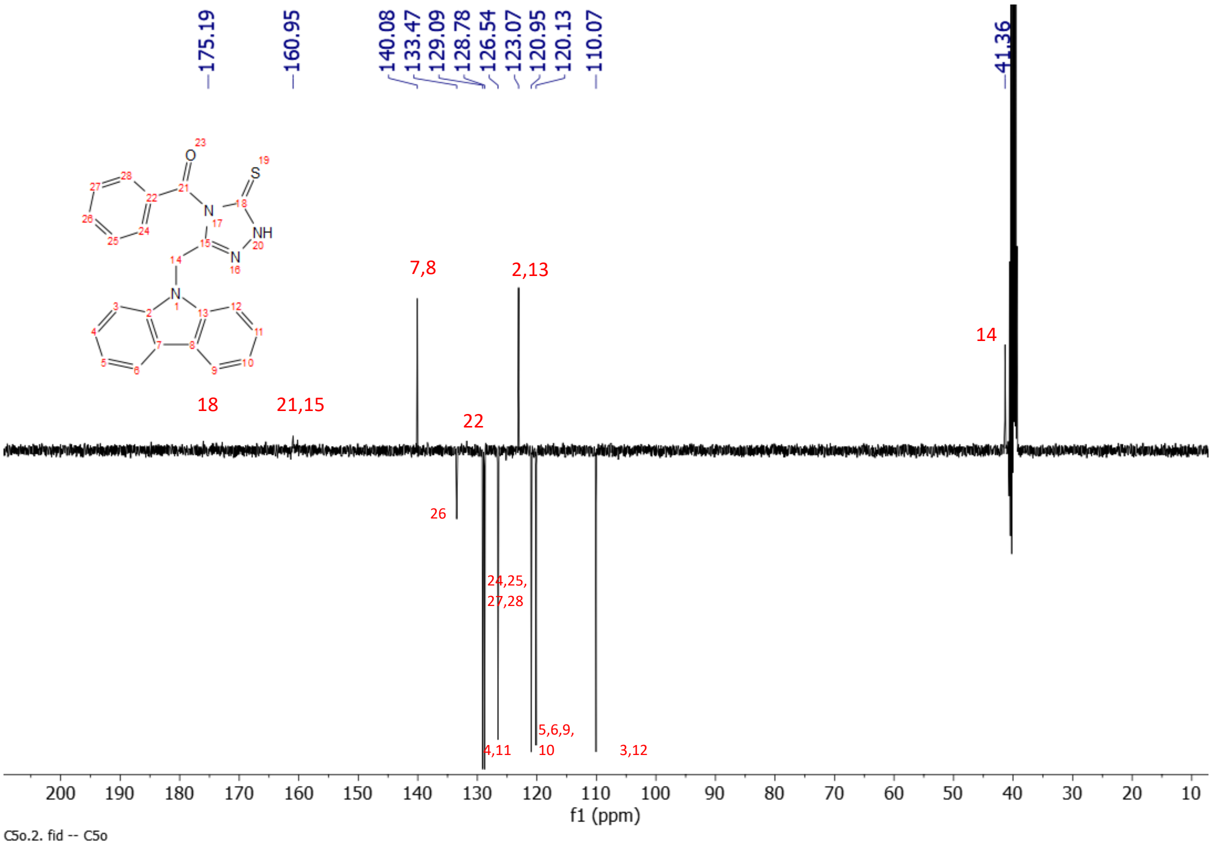

Figure S36: $^{13}C_{APT}$-NMR spectrum of Compound C5o

Spectrums of Compound C5p

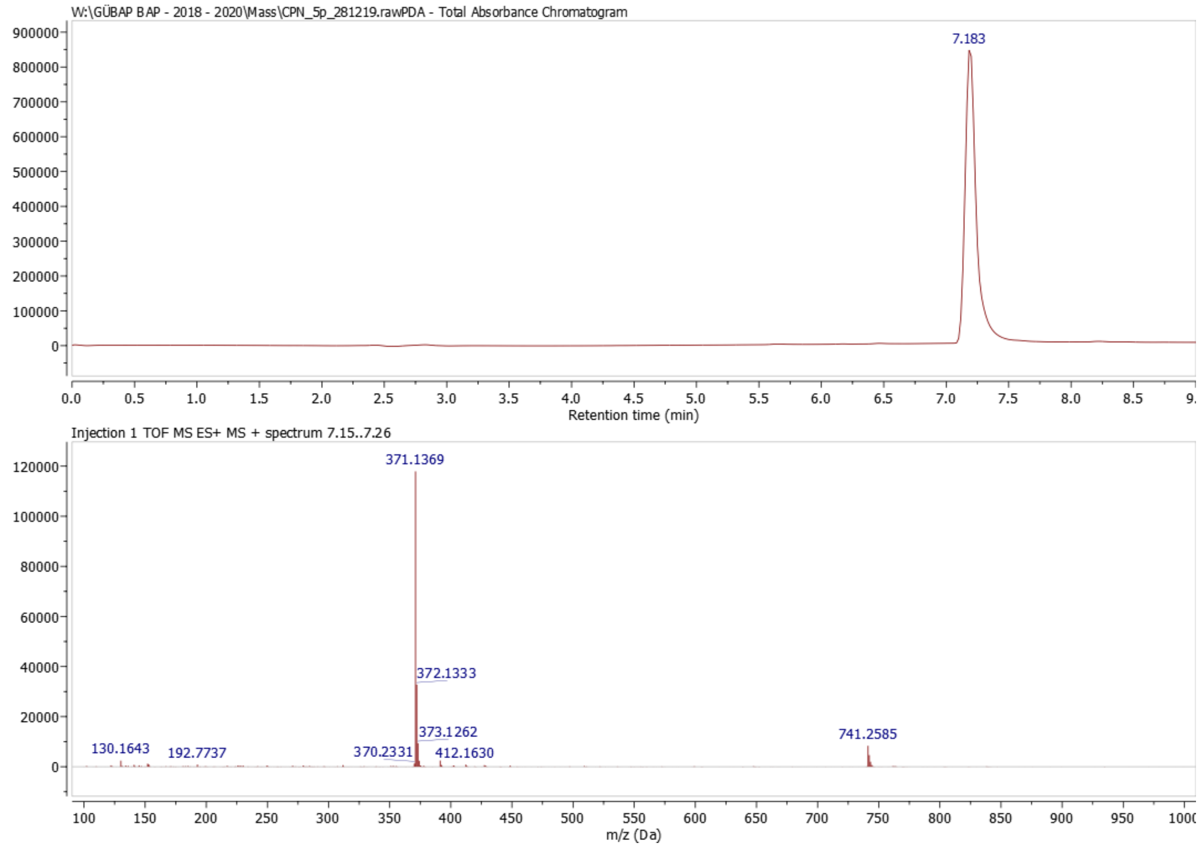

Figure S37: HRMS Spectrum of Compound C5p

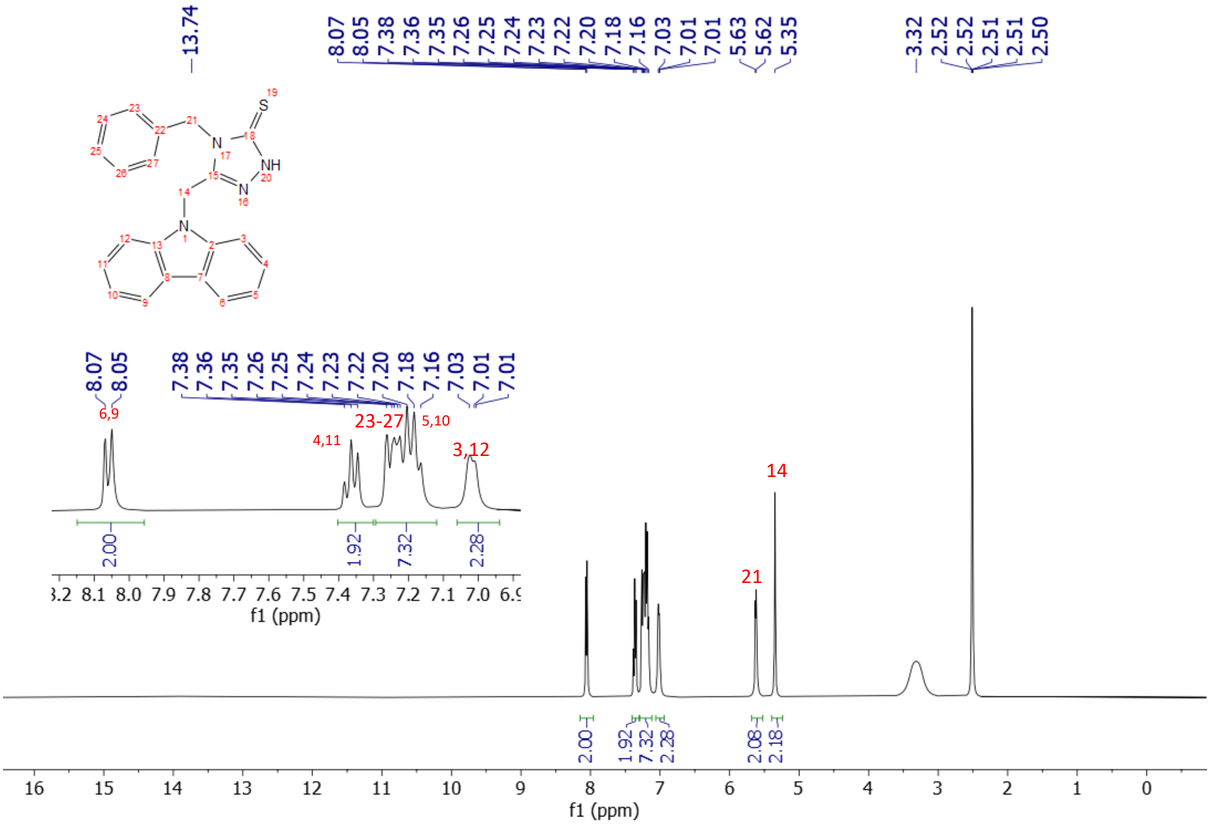

Figure S38: ^{1}H-NMR spectrum of Compound C5p

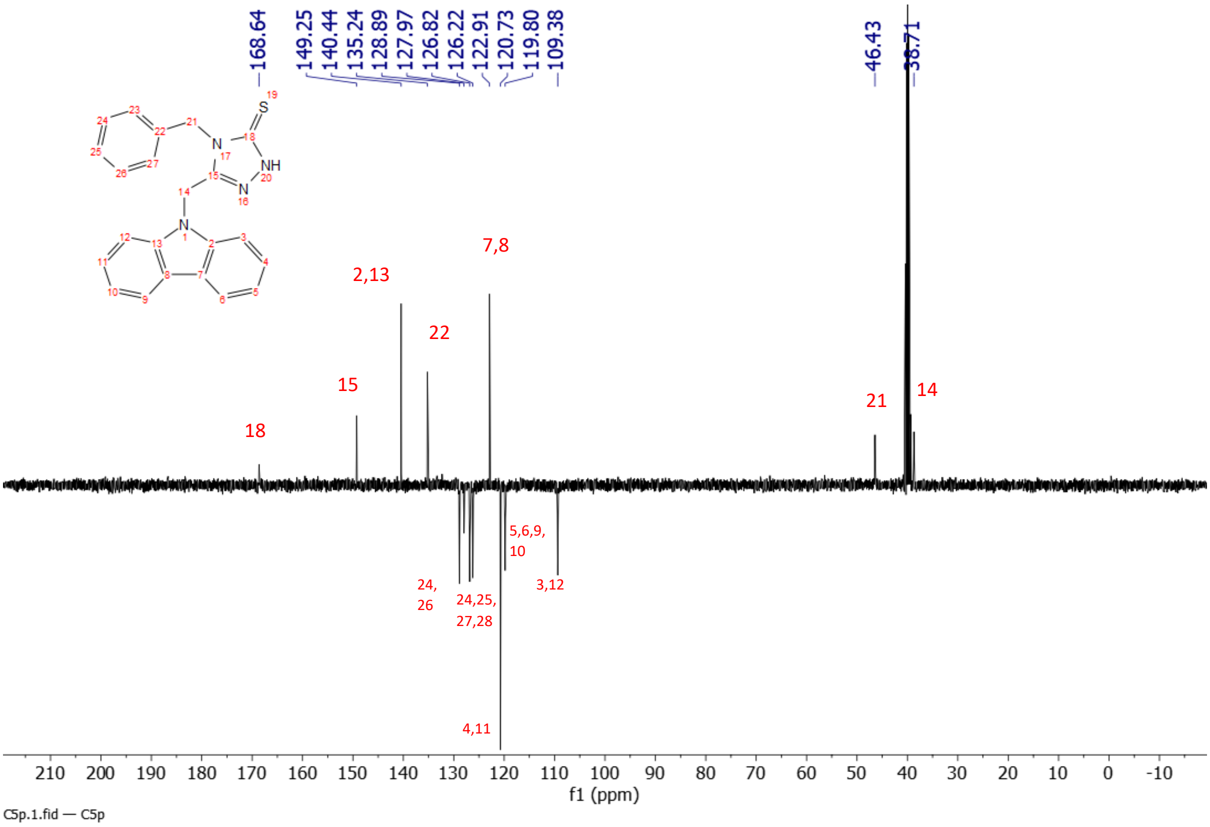

Figure S39: $^{13}C_{APT}$-NMR spectrum of Compound C5p

Spectrums of Compound C5r

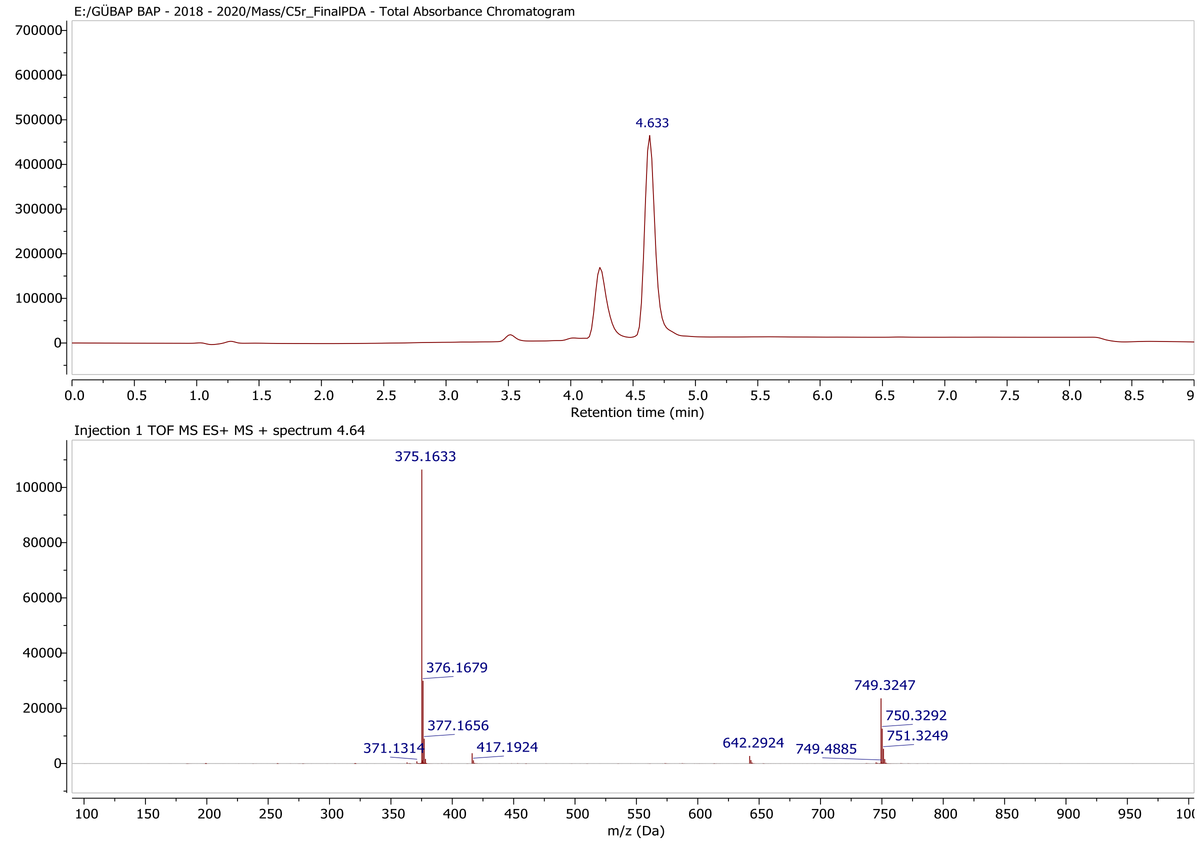

Figure S40: HRMS Spectrum of Compound C5r

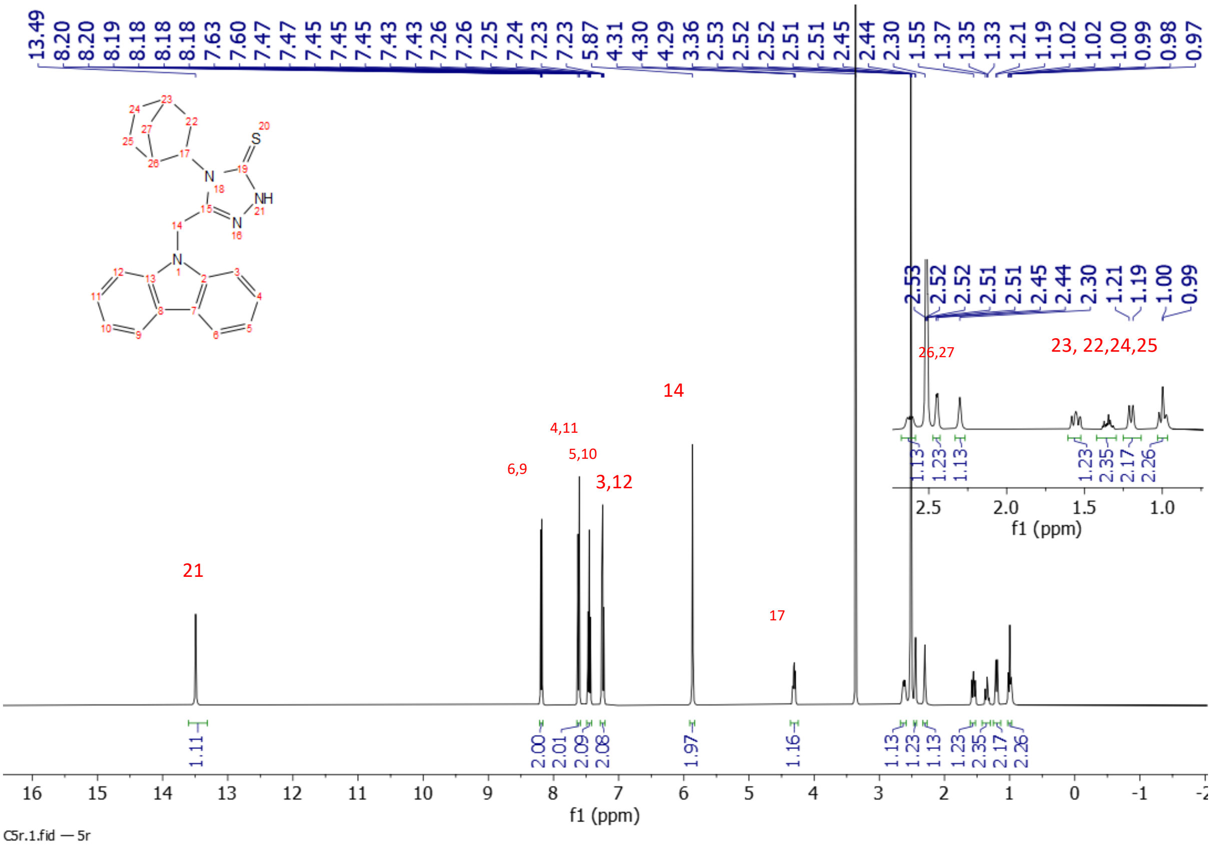

Figure S41: ¹H-NMR spectrum of Compound C5r

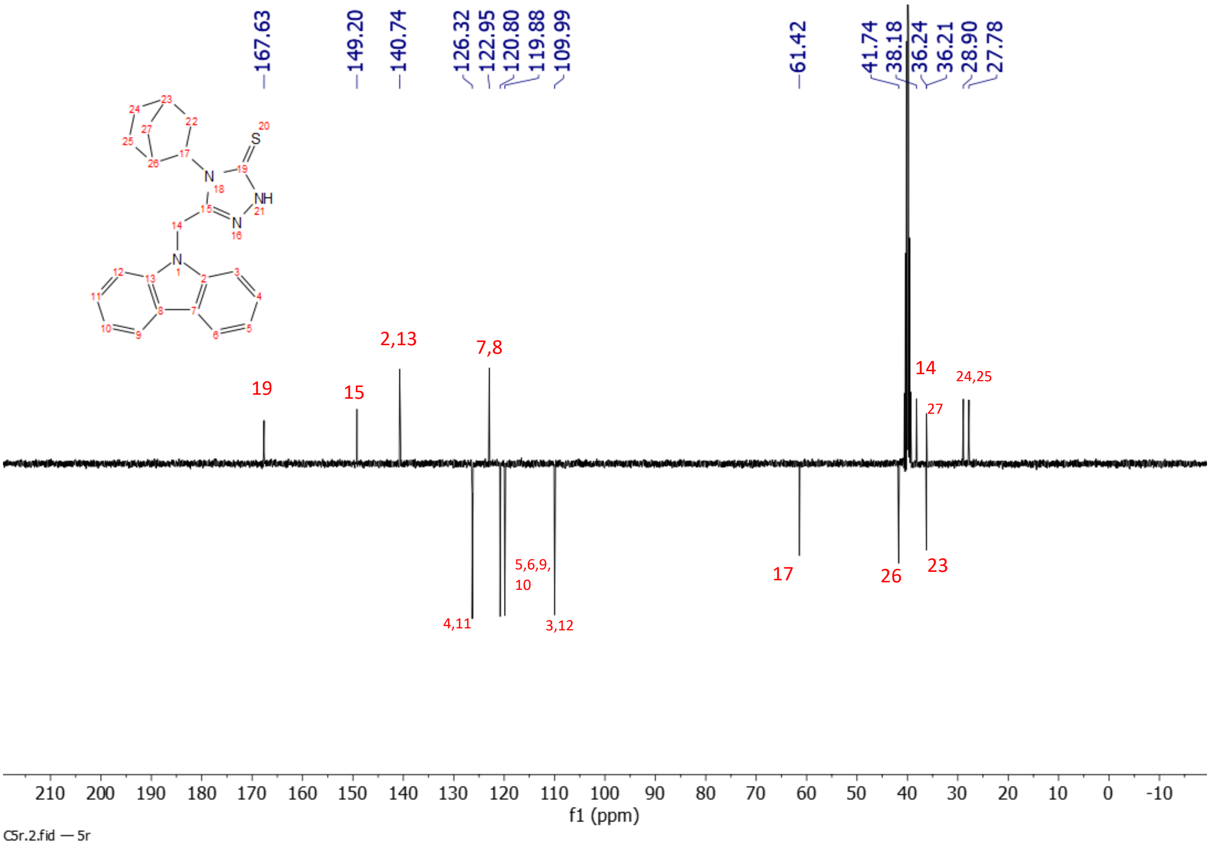

Figure S42: $^{13}C_{APT}$-NMR spectrum of Compound C5r

Spectrums of Compound C5s

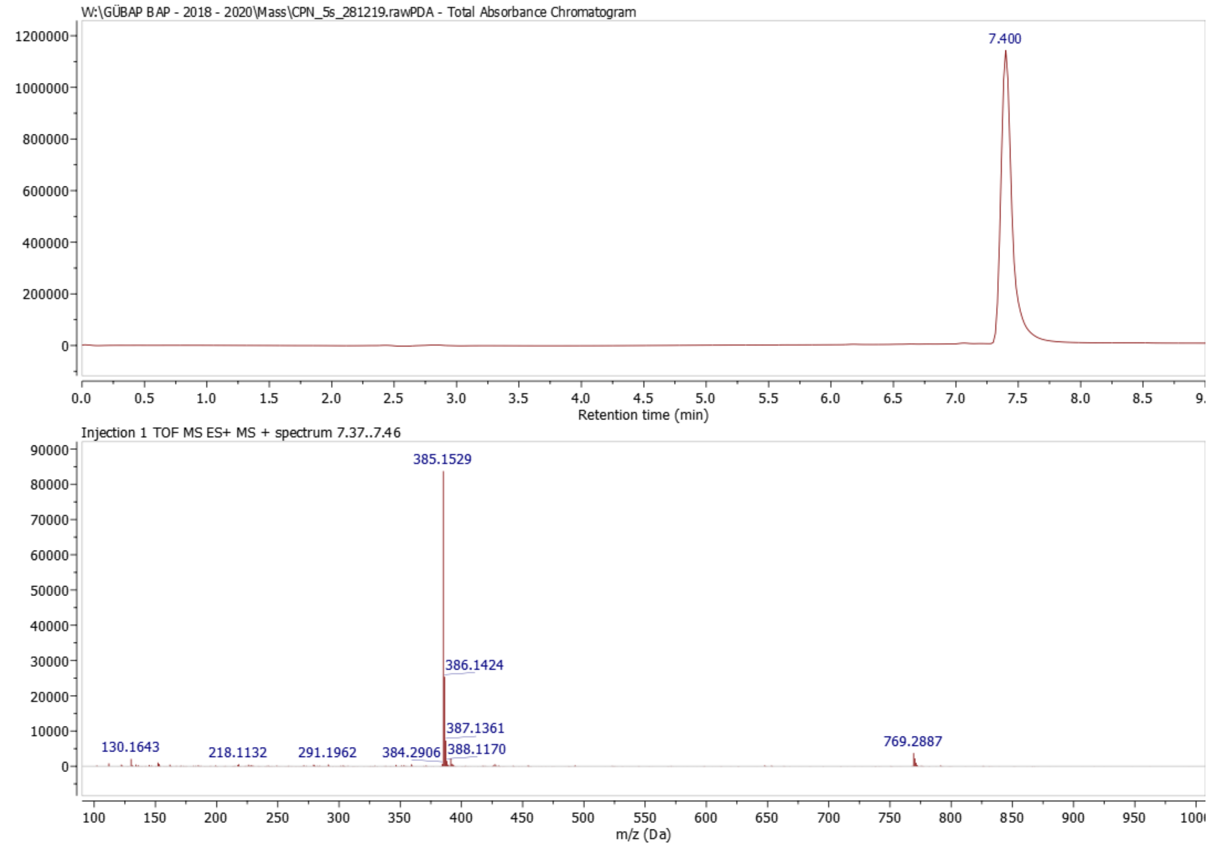

Figure S43: HRMS Spectrum of Compound C5s

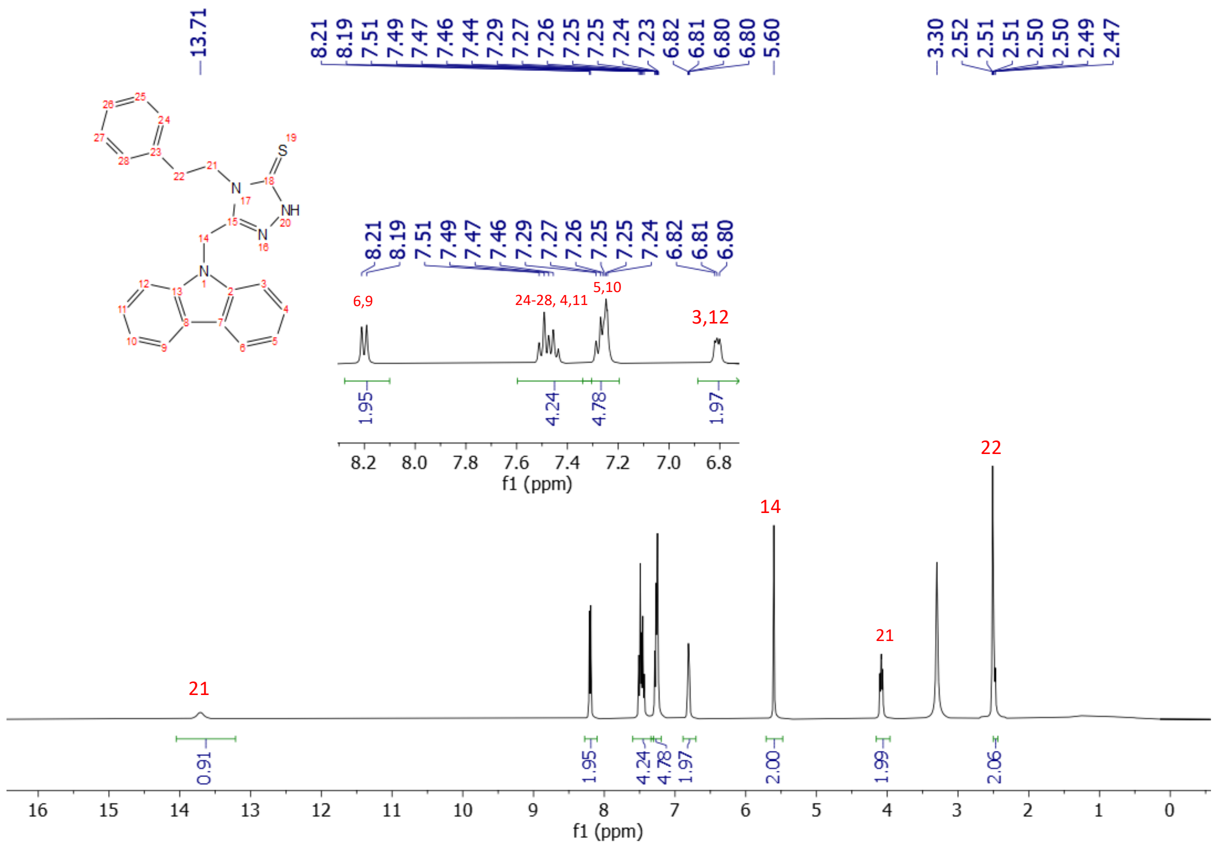

Figure S44: ^{1}H-NMR spectrum of Compound C5s

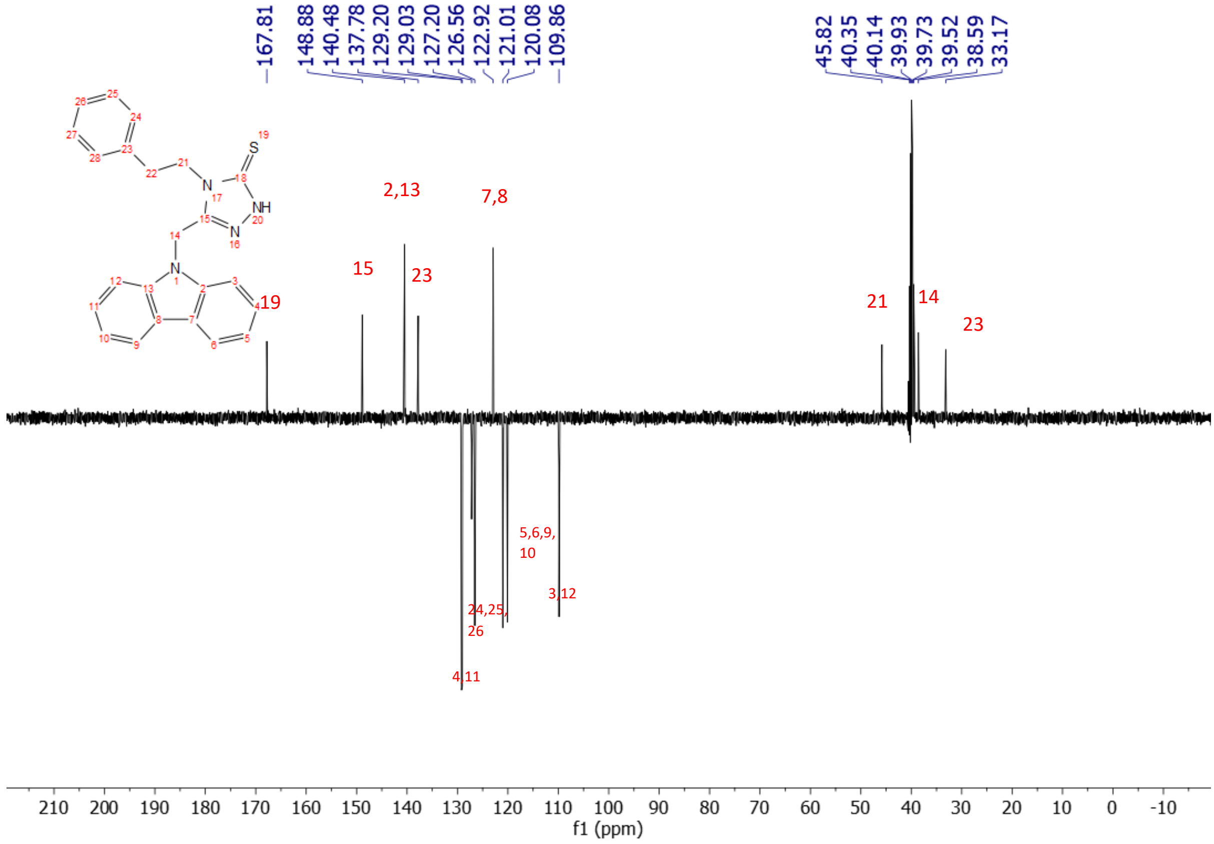

Figure S45: $^{13}C_{APT}$-NMR spectrum of Compound C5s

Spectrums of Compound C5t

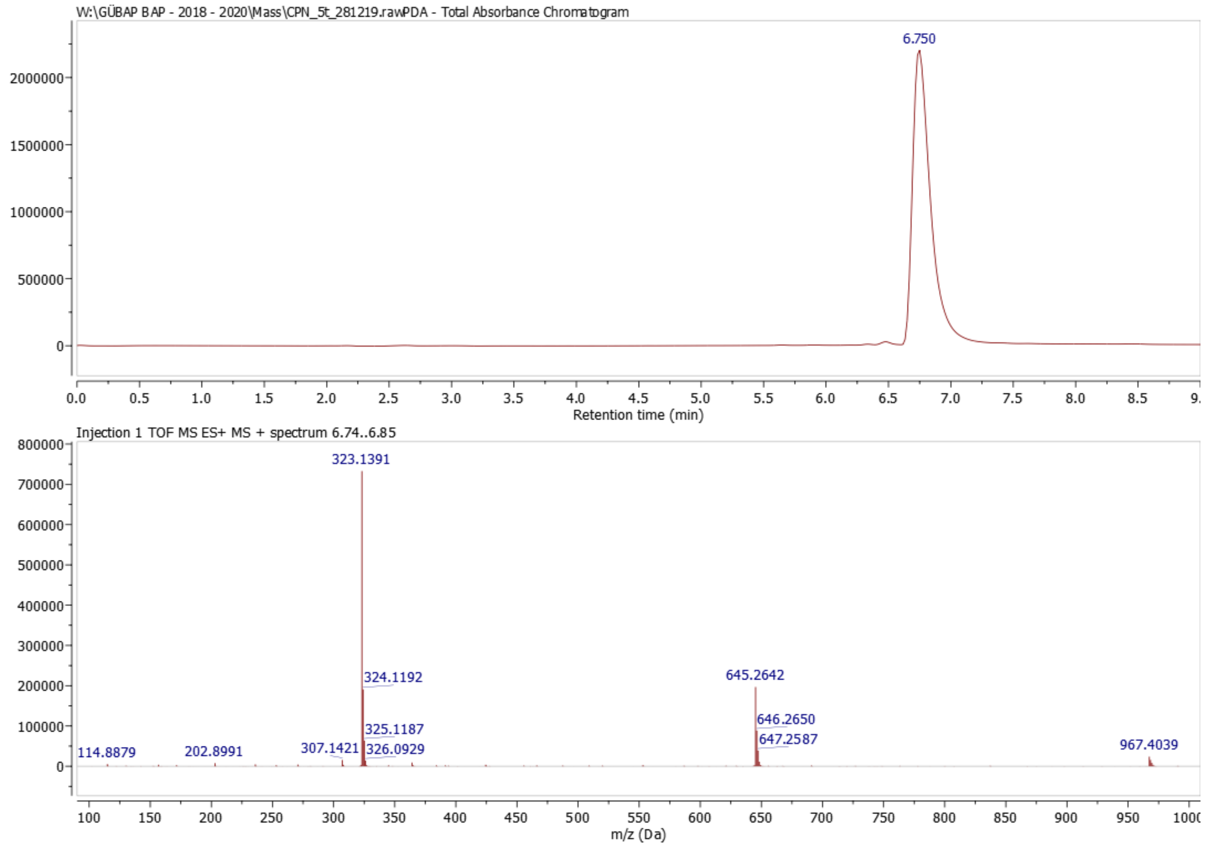

Figure S46: HRMS Spectrum of Compound C5t

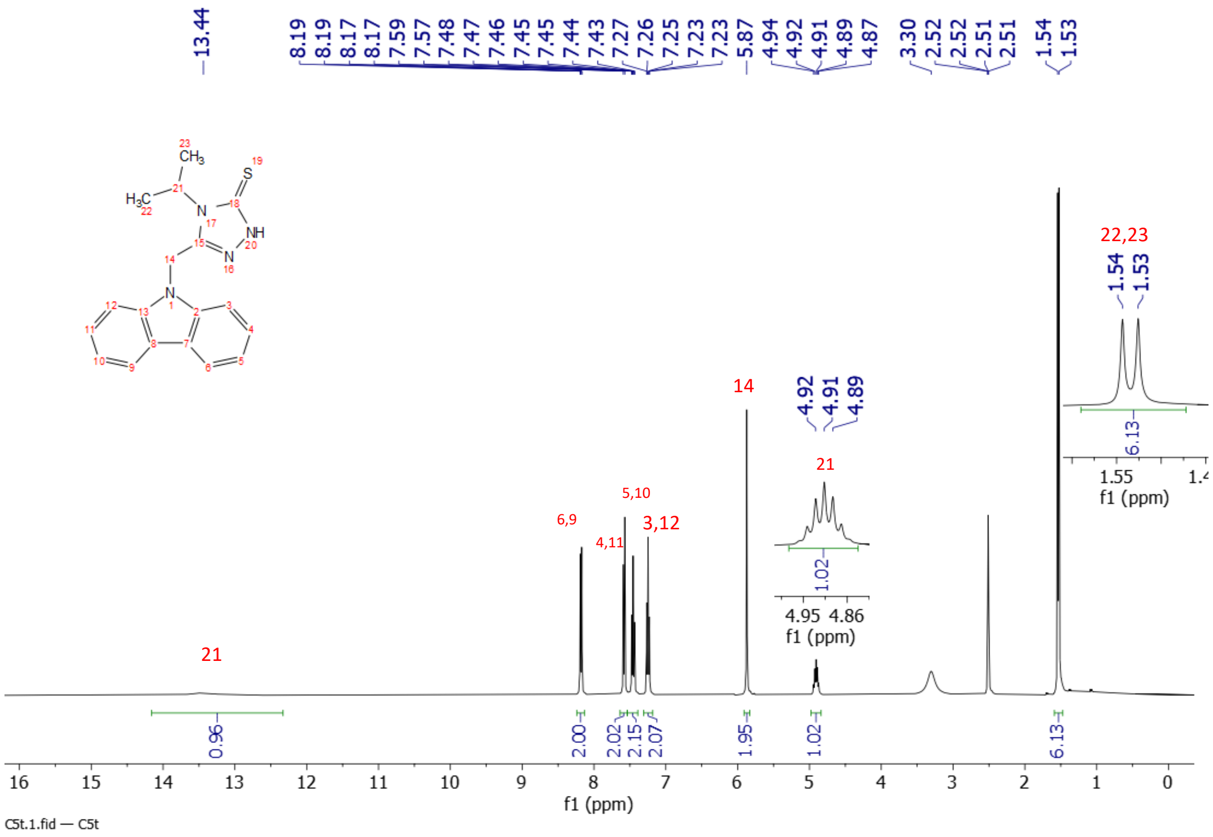

Figure S47: ^{1}H-NMR spectrum of Compound C5t

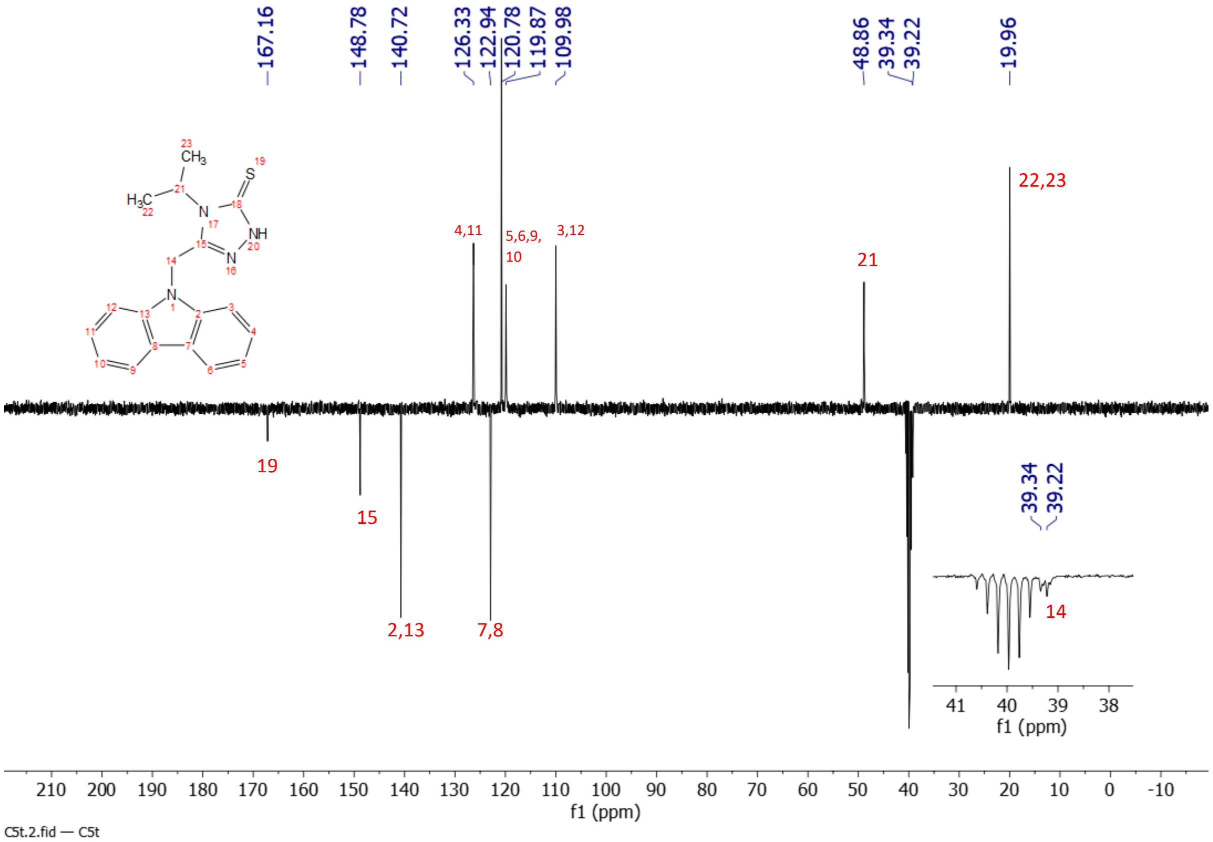

Figure S48: ^{13}C$_{APT}$-NMR spectrum of Compound C5t

Spectrums of Compound C5y

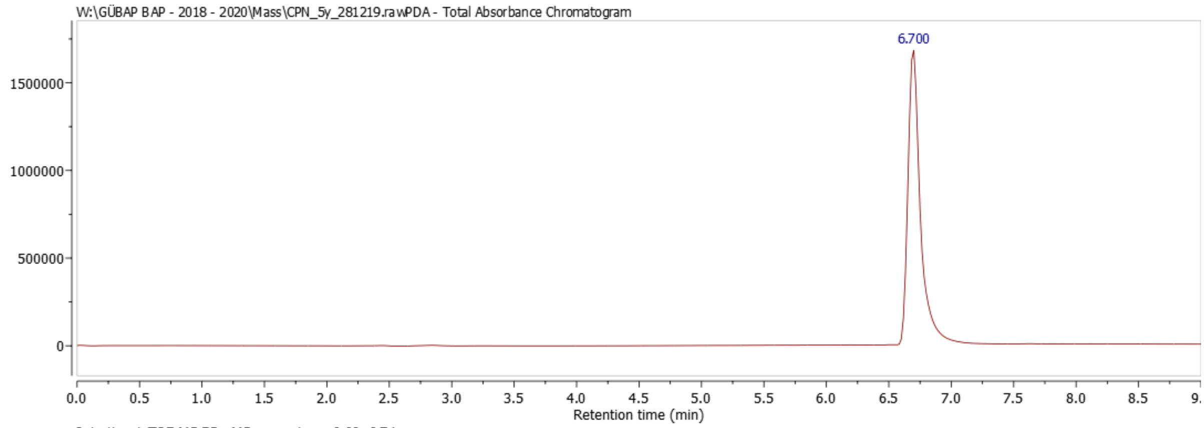

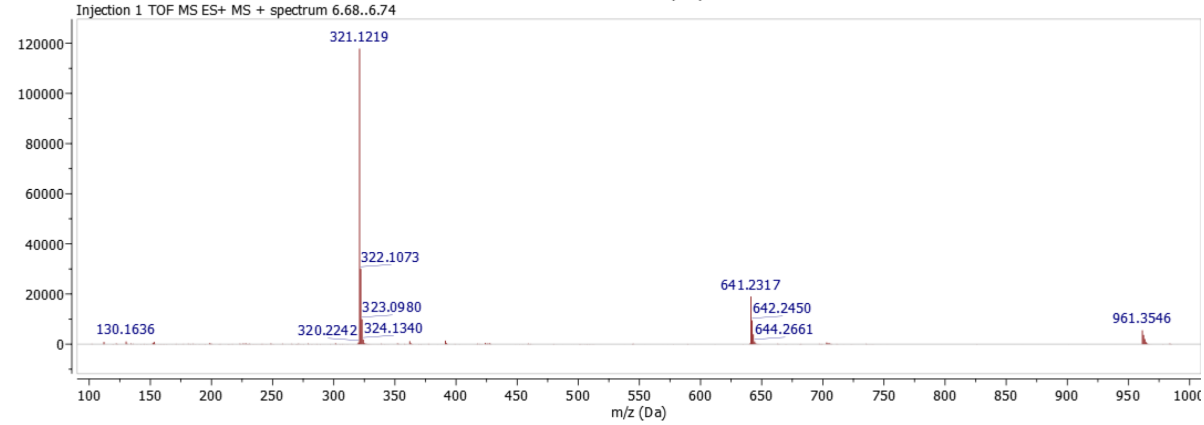

Figure S49: HRMS Spectrum of Compound C5y

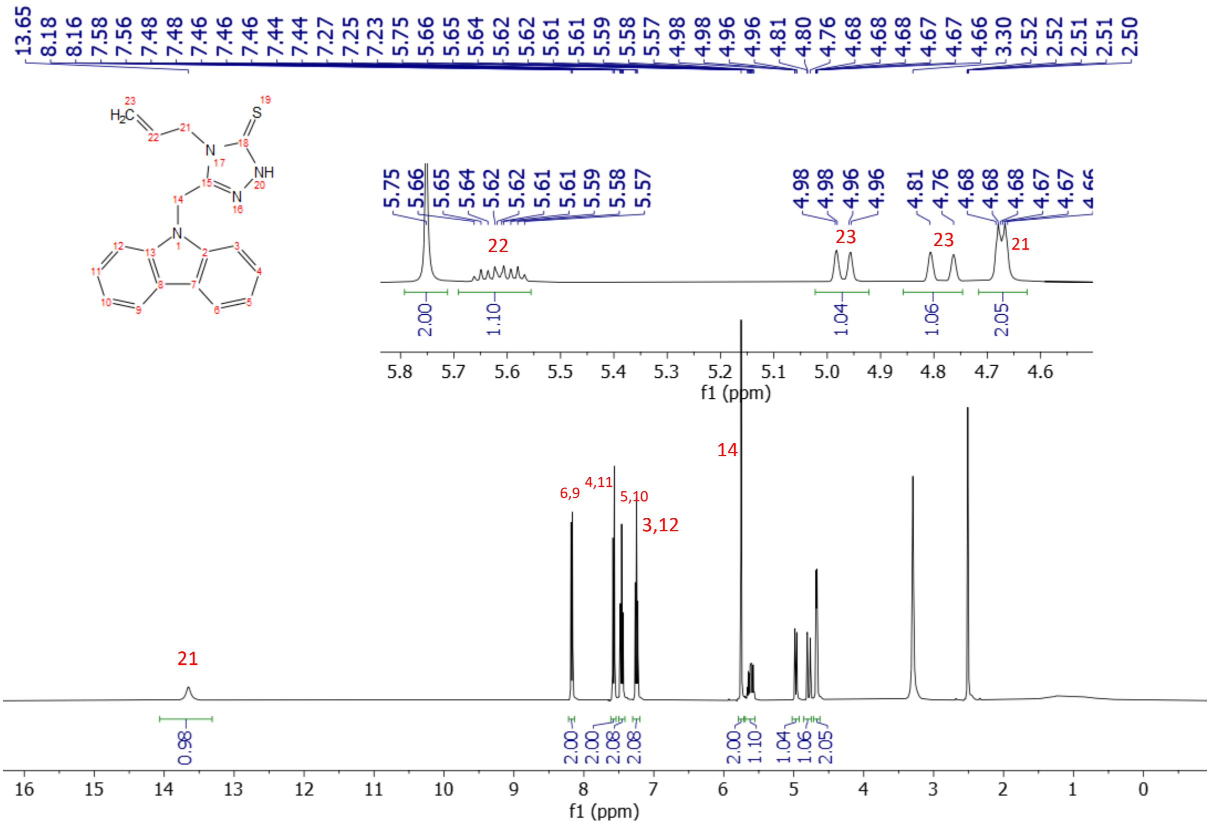

Figure S50: ^{1}H-NMR spectrum of Compound C5y

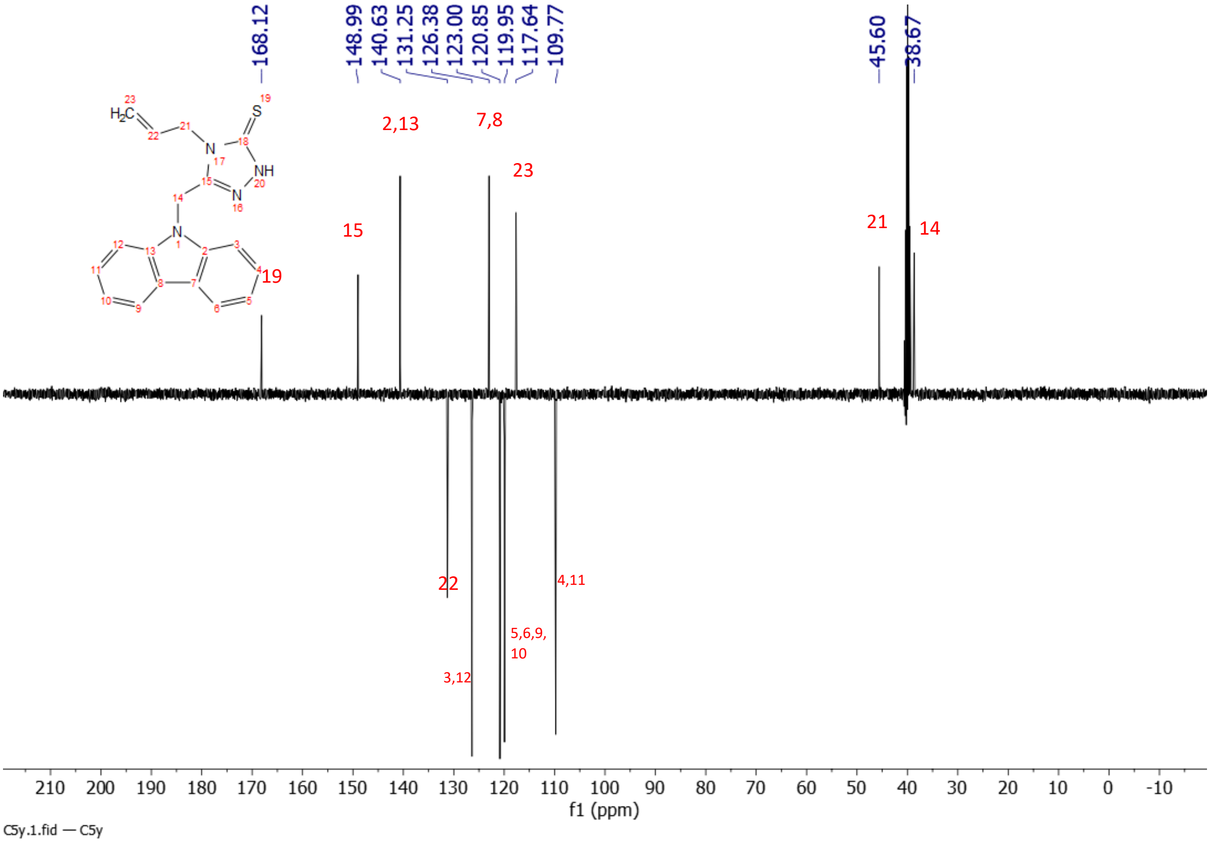

Figure S51: $^{13}C_{APT}$-NMR spectrum of Compound C5y

Spectrums of Compound C5z

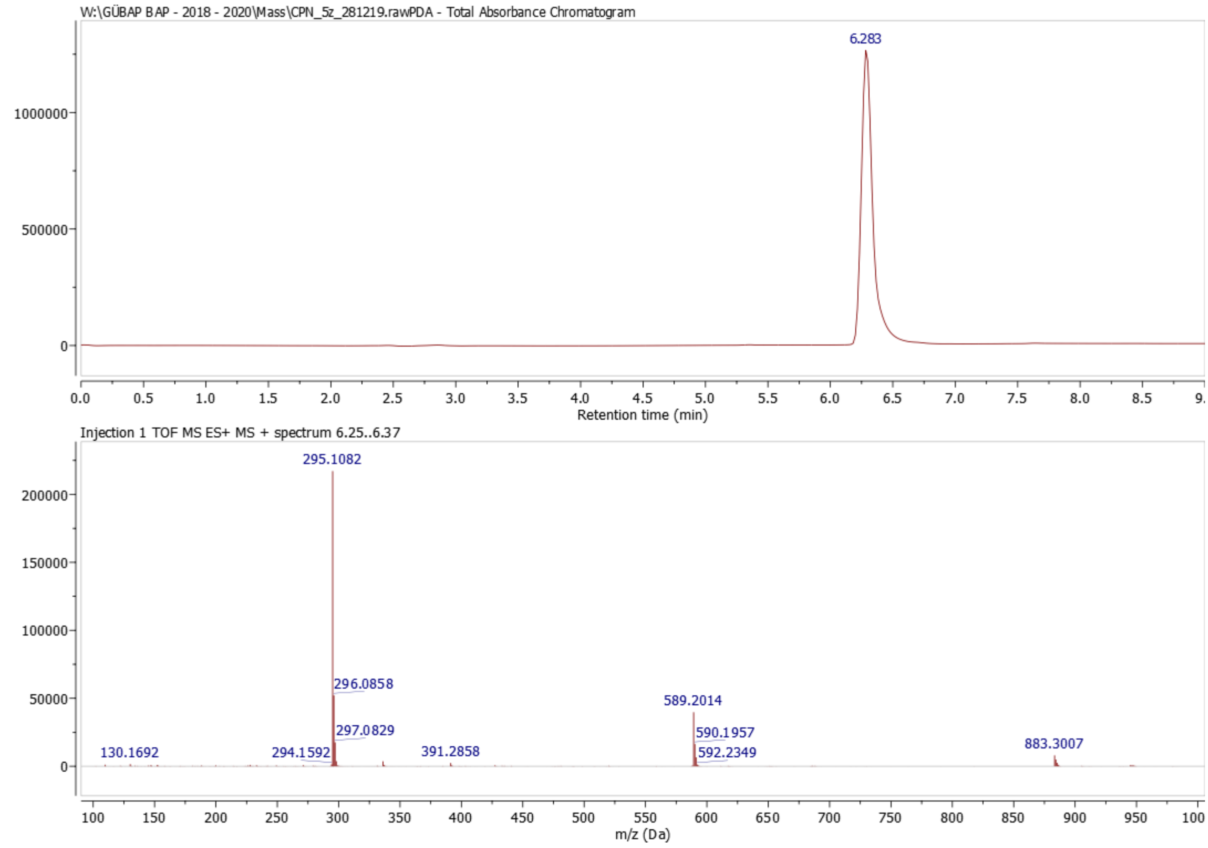

Figure S52: HRMS Spectrum of Compound C5z

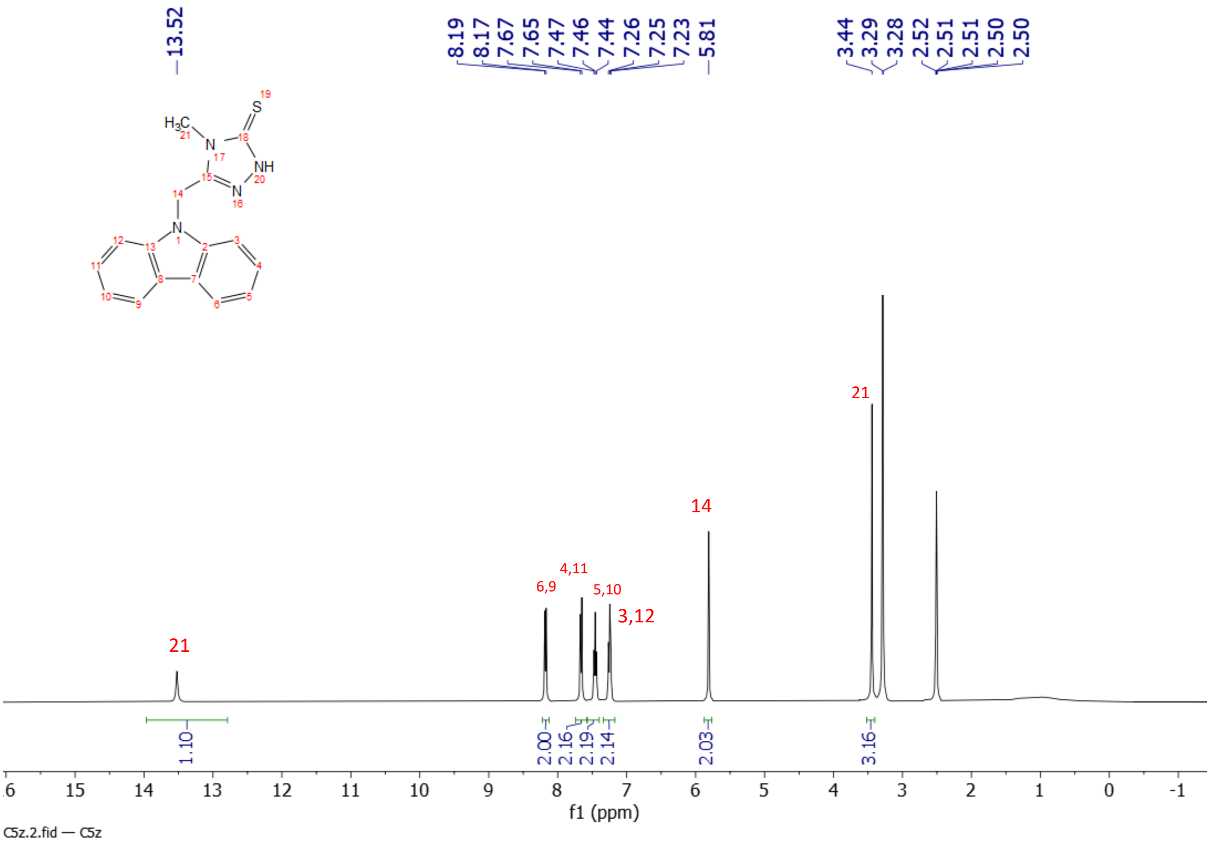

Figure S53: ¹H-NMR spectrum of Compound C5z

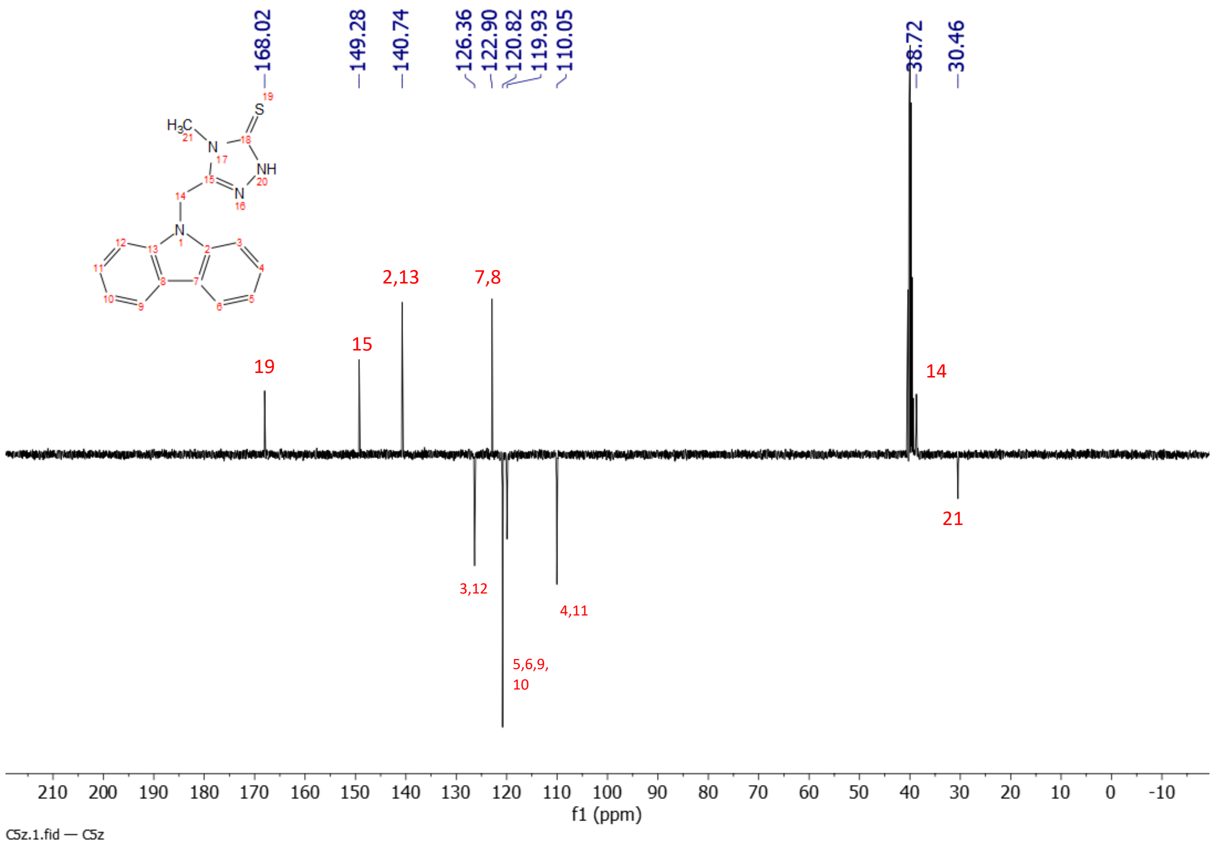

Figure S54: $^{13}C_{APT}$-NMR spectrum of Compound C5z

Table S1: ADME-T analysis of the **C5a-z** series.

**Compound**	MW	Human Oral Absorption (%)	QPlogPo/w	QPlogS	QPlogBB	TPSA (Å²)	QPlogKhsa	QPPCaco	Lipinski rule violation; drug-likeness
**C5a**	356.444	100	5.791	-6.49	0.084	74.44	1.097	3390.022	0;Yes
**C5b**	386.47	100	5.052	-6.108	-0.012	79.86	0.795	2900.215	0;Yes
**C5c**	407.532	100	4.07	-4.717	0.126	83.10	0.45	655.544	0;Yes
**C5d**	406.504	100	5.706	-6.679	0.061	70.63	1.086	2814.51	0;Yes
**C5e**	401.442	100	4.326	-6.216	-0.961	116.45	0.758	352.698	0;Yes
**C5f**	386.47	100	5.085	-6.217	-0.004	79.86	0.803	2900.216	0;Yes
**C5g**	435.34	100	5.611	-6.943	0.242	70.63	0.954	2902.366	0;Yes
**C5h**	390.889	100	5.532	-6.823	0.231	70.63	0.929	2901.383	0;Yes
**C5k**	424.443	100	6.007	-7.499	0.329	70.63	1.069	2900.216	1;Yes
**C5m**	362.492	100	5.046	-6.101	0.118	70.63	0.897	3373.343	0;Yes
**C5n**	370.471	100	5.342	-6.646	0.052	70.63	0.969	2900.216	0;Yes
**C5o**	384.455	100	5.216	-6.185	-0.357	87.70	0.923	1465.552	0;Yes
**C5p**	370.471	100	5.308	-5.774	0.083	70.63	0.817	4071.343	0;Yes
**C5q**	532.061	100	8.849	-10.374	-0.064	73.83	2.147	3353.082	2;NO
**C5r**	374.503	100	5.132	-6.287	0.092	70.63	0.936	3101.902	0;Yes
**C5s**	384.498	100	5.631	-6.251	-0.129	70.63	0.951	3095.675	0;Yes
**C5t**	322.427	100	4.259	-4.805	0.094	70.63	0.514	3403.54	0;Yes
**C5y**	320.411	100	4.364	-5.035	-0.035	70.63	0.506	2874.811	0;Yes
**C5z**	294.373	100	3.842	-4.811	0.086	70.63	0.376	2780.859	0;Yes
**Recommended values**	**130-725**	**>80% is high, <25% is low**	**-2.0-6.5**	**-6.0-0.5**	**-3 – 1.2**	**90-140**	**-1.5 – 1.5**	**<25 poor >500 great**	**Less than 2, yes**

Molecular weight (Mol._Mw), octanol/water partition coefficient (QPlogPo/w), aqueous solubility (QPlogS), brain/blood partition coefficient (QPlogBB), the ability to cross the blood-brain barrier (QPlogBB), the total polar surface area (TPSA), the binding affinity to human serum albumin (QPlogKhsa) and the Caco-2 cell permeability in nm/sec.
